# Randomly incorporated genomic N6‐methyldeoxyadenosine delays zygotic transcription initiation in a cnidarian

**DOI:** 10.15252/embj.2022112934

**Published:** 2023-07-04

**Authors:** Febrimarsa, Sebastian G Gornik, Sofia N Barreira, Miguel Salinas‐Saavedra, Christine E Schnitzler, Andreas D Baxevanis, Uri Frank

**Affiliations:** ^1^ Centre for Chromosome Biology, School of Biological and Chemical Sciences University of Galway Galway Republic of Ireland; ^2^ Computational and Statistical Genomics Branch, Division of Intramural Research National Human Genome Research Institute, National Institutes of Health Bethesda MD USA; ^3^ Whitney Laboratory for Marine Bioscience University of Florida St. Augustine FL USA; ^4^ Department of Biology University of Florida Gainesville FL USA; ^5^ Present address: Centre for Organismal Studies Heidelberg University Heidelberg Germany

**Keywords:** 6mA, cnidaria, DNA methylation, m6A, zygotic genome activation, Chromatin, Transcription & Genomics, Development

## Abstract

N6‐methyldeoxyadenosine (6mA) is a chemical alteration of DNA, observed across all realms of life. Although the functions of 6mA are well understood in bacteria and protists, its roles in animal genomes have been controversial. We show that 6mA randomly accumulates in early embryos of the cnidarian *Hydractinia symbiolongicarpus*, with a peak at the 16‐cell stage followed by clearance to background levels two cell cycles later, at the 64‐cell stage—the embryonic stage at which zygotic genome activation occurs in this animal. Knocking down *Alkbh1*, a putative initiator of animal 6mA clearance, resulted in higher levels of 6mA at the 64‐cell stage and a delay in the initiation of zygotic transcription. Our data are consistent with 6mA originating from recycled nucleotides of degraded m6A‐marked maternal RNA postfertilization. Therefore, while 6mA does not function as an epigenetic mark in *Hydractinia*, its random incorporation into the early embryonic genome inhibits transcription. In turn, Alkbh1 functions as a genomic 6mA “cleaner,” facilitating timely zygotic genome activation. Given the random nature of genomic 6mA accumulation and its ability to interfere with gene expression, defects in 6mA clearance may represent a hitherto unknown cause of various pathologies.

## Graphical abstract

N6‐methyladenosine‐decorated maternal RNA is the source of 6mA in the embryonic genome of the cnidarian *Hydractinia symbiolongicarpus*.

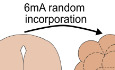

## Introduction

Methylation of adenine in DNA (6mA) and the functions it fulfills are well documented in bacteria (Geier & Modrich, 1979; Lahue *et al*, 1987; Slater *et al*, 1995; Haagmans & van Der Woude, 2000) and protists (Rae  & Steele, 1978; Fu *et al*, 2015; Chen *et al*, 2018a; Beh *et al*, 2019; Wang *et al*, 2019), but studies on this DNA modification in animals have revealed conflicting reports (Douvlataniotis *et al*, 2020; Bochtler & Fernandes, 2021; Kong *et al*, 2022). Low levels of 6mA were reported in the genomes of flies (Zhang *et al*, 2015; Yao *et al*, 2018; He *et al*, 2019; Bochtler & Fernandes, 2021), worms (Greer *et al*, 2015; O'Brown *et al*, 2019), fish (Liu *et al*, 2016a; O'Brown *et al*, 2019), and mammalian cells (Koziol *et al*, 2016; Wu *et al*, 2016; Xiao *et al*, 2018; Xie *et al*, 2018) and were shown to correlate with transposon transcripts level in flies and mouse cells (Zhang *et al*, 2015; Wu *et al*, 2016; Xie *et al*, 2018). However, some of these studies were challenged by others, attributing their findings to antibody artifacts (Abakir *et al*, 2020; Douvlataniotis *et al*, 2020) or to bacterial contamination (Schiffers *et al*, 2017; O'Brown *et al*, 2019; Kong *et al*, 2022).

To address this apparent discrepancy, we have studied 6mA during early embryogenesis of *Hydractinia symbiolongicarpus*, a member of the early‐diverging phylum Cnidaria. As a sister group to Bilateria, cnidarians may provide new insights into the evolution of animal traits. We report a peak in the level of 6mA in 16‐cell stage embryos. However, 6mA marks were randomly distributed in the genome, inconsistent with having an epigenetic function. We find that the clearance of 6mA before the 64‐cell stage by the dioxygenase Alkbh1 is necessary for timely zygotic genome activation (ZGA). We propose that 6mA is passively and randomly accumulated in the genome due to the rapid degradation of m6A‐marked maternal RNA, NTP‐dNTP conversion by ribonucleotide reductase, and random integration into the early embryonic genome.

## Results

### Dynamics and distribution of 6mA during embryogenesis

**Figure 1:**
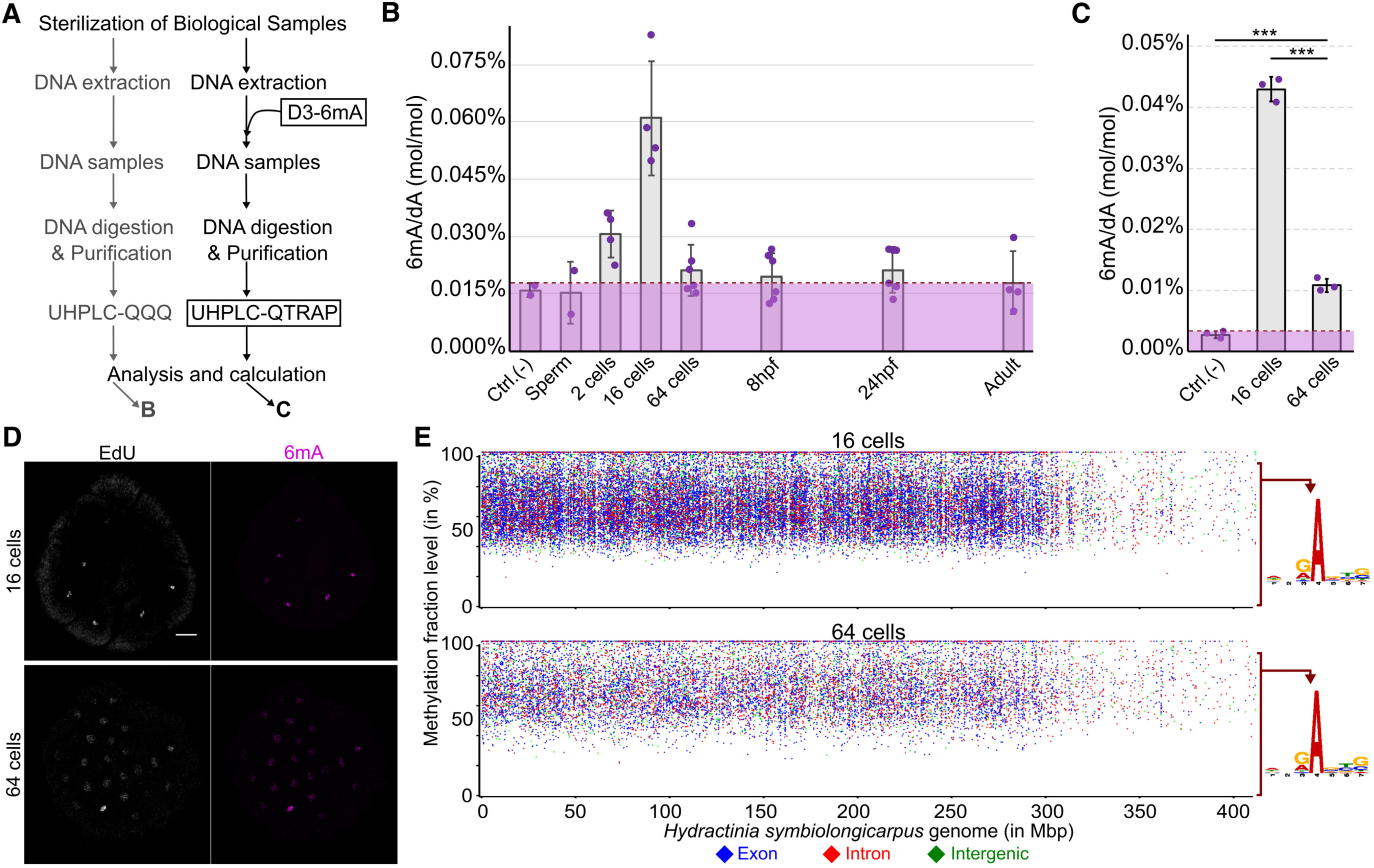
Dynamics and distribution of 6mA during *Hydractinia* early embryogenesis Schematic of the two independent experiments performed to measure 6mA/dA levels.6mA/dA levels (mol/mol) of seven stages of *Hydractinia* development.6mA/dA levels (mol/mol) of 16‐ and 64‐cells of *Hydractinia* embryos.Whole‐mount immunofluorescence of 6mA from 16‐ and 64‐cell stages of *Hydractinia*. Samples were RNase‐treated.Distribution of A sites that were detected to be methylated in the genomes of 16‐ and 64‐cell stage, plotted against the percentage of SMRT‐seq reads that showed methylation at each site. Consensus sequences of 6mA sites where the methylation level is between 0 and 95% are shown right to the graph, indicating that no motif can be deduced. Data information: In (B–C), purple area indicates the background level of 6mA contamination from the digestive enzymes used in the respective experiment. hpf: hours post fertilization. The number of biological replicates used for each sample is indicated by the amount of data points in the graph. *P < 0.05, **P < 0.01, ***P < 0.005, and n.s., not significant, unpaired two‐tailed Student's *t*‐test. In (D), the scale bare is equal to 20 μm. All error bars indicate standard deviation. Source data are available online for this figure.

**Figure EV1:**
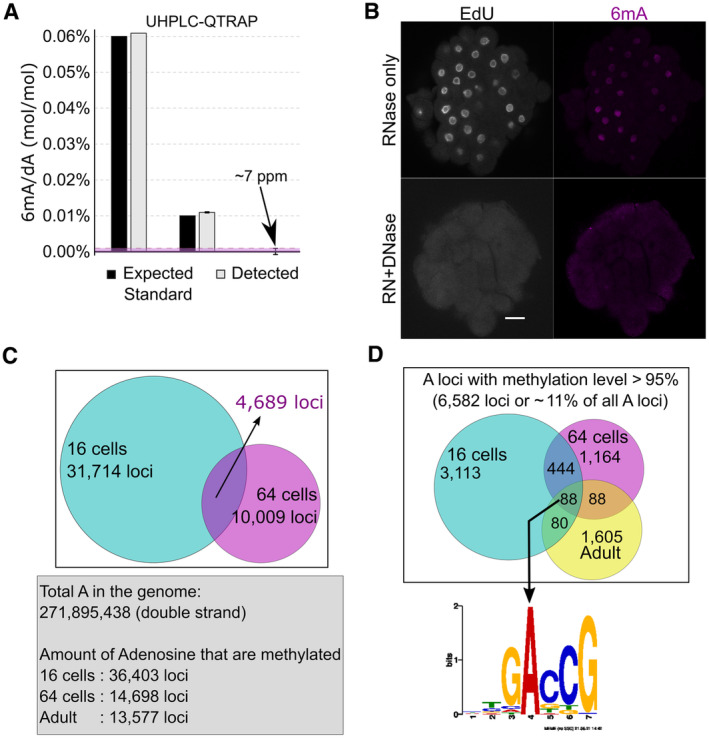
Detection and distribution of 6mA in the genome of *Hydractinia symbiolongicarpus* Detection of 6mA in technical replicates (*n*) of reference solutions (0%, *n* = 3; 0.01%, *n* = 2; 0.06%, *n* = 1) by UHPLC‐QTRAP. Error bars indicate standard deviation.DNase but not RNase treatment can abolish the signal of anti‐6mA immunofluorescence. Scale bars: 20 μm.Venn diagram displaying the overlapping methylated A sites between 16‐cell and 64‐cell genomes.Venn diagram displaying the overlapping A sites between three genome that are always methylated (> 95%) and the consensus sequence generated by MEME‐Chip of the 88 overlapping methylated A‐loci. Source data are available online for this figure.

To quantitatively assess 6mA levels in *Hydractinia*, we extracted genomic DNA from adult specimens and from different embryonic stages. The samples were then enzymatically digested and purified. Synthetic oligonucleotides containing 6mA were similarly treated and used as external standards for ultra‐high‐performance liquid chromatography coupled with triple quadrupole tandem mass spectrometry (UHPLC‐QQQ) (Fig 1A; pipeline on the left side). We found high 6mA/A background level from the negative control (Fig 1B, purple area with dashed line at the top), indicating high levels of 6mA contamination from the bacterial source of the enzymes used in this experiment. We found that the levels of 6mA were at background level in sperm and slightly above background at the two‐cell stage. 6mA increased gradually with a peak at the 16‐cell stage, rapidly decreasing to background level by the 64‐cell stage. These low levels were maintained to adulthood, being indistinguishable from the negative control (Fig 1B). Due to the high background level of 6mA/A detected in this first set of experiments, we reanalyzed the level of 6mA/dA in 16‐ and 64‐cell stage embryos by digesting their DNA using a different batch of enzymes and analyzing the samples with ultra‐high‐performance liquid chromatography coupled with quadrupole ion trap tandem mass spectrometry (UHPLC‐QTRAP). Stable isotope‐labeled [^3^D_1_]‐6mA was used as an internal standard for sample enrichment and quantitation (Fig 1A; pipeline on the right side). This method enabled us to detect 6mA/A levels of 0.01% as being distinct from the negative control (Figs 1C and EV1A) and confirmed the 6mA dynamics at the 16‐ and 64‐cell stages. Hence, 6mA levels are low in early embryos, high at the 16‐cell stage, and low again at the 64‐cell stage and later (Fig 1B and C).

To rule out the possibility of bacterial contamination with high amounts of 6mA, we used an anti‐6mA antibody for immunofluorescence (IF) in fixed embryos. The 6mA signal was visible in nuclei of *Hydractinia* cells (Fig 1D; Appendix Fig S1A) and could be abolished by DNase treatment, but not by RNase treatment (Fig EV1B); this observation is consistent with methylation of the animal's nuclear DNA.

Next, we performed single‐molecule real‐time sequencing (SMRT‐Seq) to investigate the distribution of 6mA in the genome of 16‐ and 64‐cell stage embryos and adults. The data of methylated A sites were filtered by a combination of interpulse duration (IPD) ratio > 3.0, read count > 10, and *P*‐value < 0.05, following a recently published guideline for multicellular eukaryotes (Zhu *et al*, 2018). Overall, the numbers of methylated A‐loci were consistent with the dynamics of the 6mA/A detected by UHPLC‐QTRAP, being high at the 16‐cell stage and low at the 64‐cell stage (Fig EV1C). However, over 90% of A‐loci were found to be inconsistently methylated across SMRT‐seq reads from any given developmental stage (16‐ and 64‐cell embryos, and adults; Fig 1E; Appendix Fig S1B and Dataset EV1), indicating heterogeneity in methylated A‐loci across cells that are expected to be uniform, particularly at the 16‐cell stage (Kraus *et al*, 2014). Only about 7% of the loci were methylated in 100% of the reads (Dataset EV1), and only 532 of the loci that were methylated in over 95% were shared between the 16‐ and 64‐cell stages (Fig EV1D). Finally, no motif representing the sequence context of all 6mA loci could be generated (Fig 1E; Appendix Fig S2). The motif generated from the 88 loci that were methylated in over 95% of the reads across all developmental stages examined was 5′‐GACCG‐3′ (Fig EV1D). This motif does not include an ApT context, suggesting that 6mA is not heritable in *Hydractinia* (Fig EV1D; Appendix Fig S2). Based on the above data, we concluded that 6mA marks are randomly distributed in the embryonic genome.

### *Alkbh1* acts as a 6mA eraser in *Hydractinia* embryos

**Figure 2:**
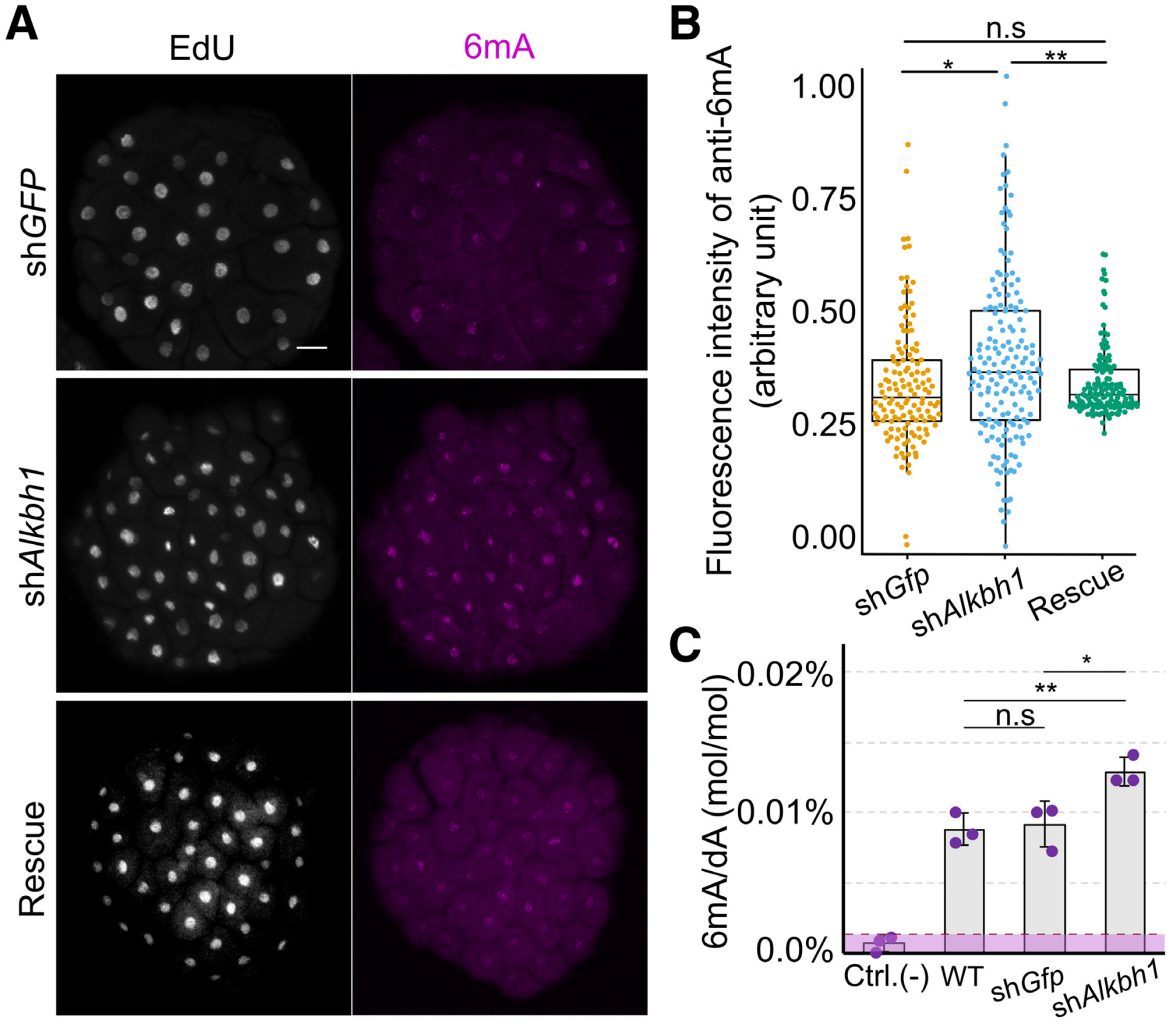
Alkbh1 removes genomic 6mA in *Hydractinia* embryos Whole‐mount immunofluorescence of anti‐6mA in 64‐cell embryos upon injection of shGFP (as control), shAlkbh1, and rescue (see text).Relative quantification of anti‐6mA signals from immunofluorescence images (see Materials and Methods). Fluorescence intensity was normalized to the highest and lowest measured area in shGFP (*n* = 135), shAlkbh1 (*n* = 168), and rescue (*n* = 140), where *n* = nuclei numbers. Central band shows the mean, the boxes show lower and upper quartiles, and whiskers show minimum and maximum data values.Quantification of *shAlkbh1*‐electroporated embryos showing significantly higher level of 6mA/dA (*P* < 0.05) compared with *shGFP* electroporated embryos and to wild type embryos at 64–cell stage. Data information: In (A), the scale bare is equal to 20 μm. In (B–C), *P < 0.05, **P < 0.01, ***P < 0.005, and n.s., not significant, unpaired two‐tailed Student's *t*‐test. All error bars indicate standard deviation. Source data are available online for this figure.

ALKBH1 has been reported to function as a 6mA demethylation initiator enzyme in animals (Wu *et al*, 2016; Tian *et al*, 2020). The *Hydractinia* genome encodes a single *Alkbh1* homolog (Appendix Fig S3 and the associated source data) that we tested to deduce its potential role in 6mA clearance. For this, we designed a specific shRNA targeting *Alkbh1* (*shAlkbh1*; Appendix Fig S4A and Appendix Table S1) and injected it into zygotes. Embryos injected with a shRNA targeting *GFP* (*shGFP*) were used as a negative control (Fig 2A). Confocal imaging of anti‐6mA immunofluorescence in 64‐cell embryos showed that, in *shAlkbh1* injected embryos, 6mA signals were higher when compared with those from *shGFP*‐injected ones (Fig 2A and B). Co‐injection of *shAlkbh1* and *Alkbh1* mRNA carrying four silent mutations (rendering it resistant to the *shAlkbh1*) partially rescued the 6mA signal (Fig 2A and B). To confirm these results, we electroporated *shAlkbh1* into zygotes, extracted genomic DNA at the 64‐cell stage, and then analyzed the 6mA content by UHPLC‐QTRAP mass spectrometry with [^3^D_1_]‐6mA as internal standard. We found a significantly higher level of 6mA in *shAlkbh1* electroporated embryos as compared to *shGFP* electroporated ones at the 64‐cell stage (Fig 2C), consistent with what was observed in the above‐described IF studies. These results confirm that Alkbh1 acts in erasing 6mA from the genome of early *Hydractinia* embryos.

### Zygotic genome activation follows 6mA clearance

**Figure 3:**
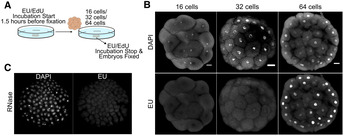
Zygotic Genome Activation at the 64‐cell stage of *Hydractinia* embryos EU/EdU incorporation experiment setup.High EU incorporation in 64‐cell but undetectable in 16‐cell embryos of *Hydractinia*.RNase treatment abolishes the EU signal. Data information: In (B), scale bar is equal to 20 μm. Source data are available online for this figure.

In many animals, early embryos rely on maternal RNAs, activating their own genomes only at later developmental stages. Given the dynamic levels of 6mA in early embryos, we hypothesized that 6mA regulates the activation of the *Hydractinia* zygotic genome. To determine the stage at which zygotic transcription is activated, we used EU incorporation assays to visualize nascent RNA (Fig 3A and Appendix Fig S4B) and established that a major transcriptional wave commences at the 64‐cell stage, with little or no EU incorporation observed in earlier stages (Fig 3B). Therefore, it appears that a major wave of ZGA occurs immediately following the clearance of 6mA from the embryonic genome (Figs 1B and C, and 3B).

### Alkbh1 knockdown delays zygotic genome activation

**Figure 4:**
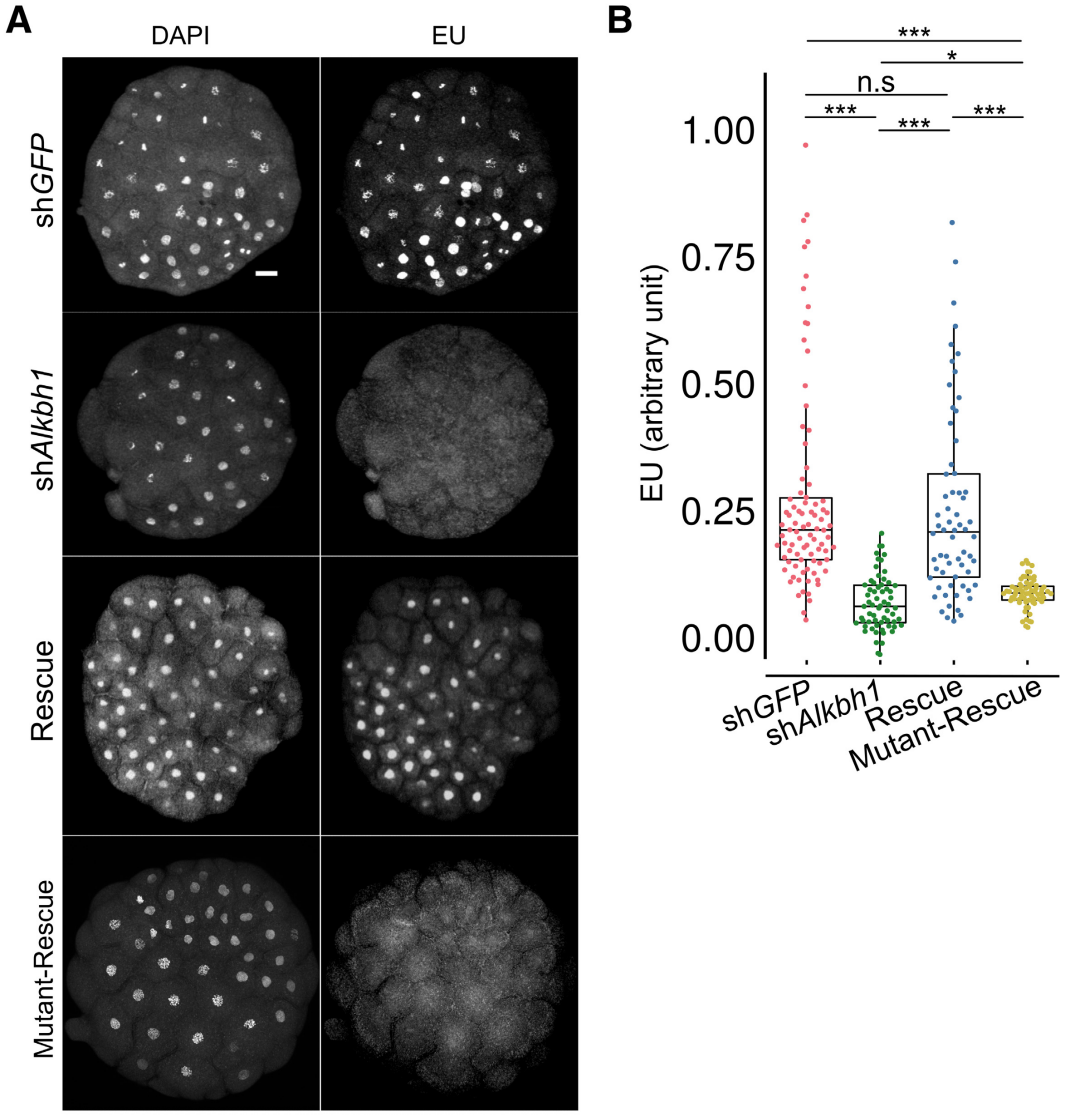
Knockdown of *Alkbh1* delays zygotic genome activation in *Hydractinia* Whole‐mount image of EU incorporation signals at 64 cells upon injection with *shGFP*, *shAlkbh1*, rescue, and mutant‐rescue solution (see text).Relative quantification of EU signals (see Materials and Methods). Fluorescence intensity was normalized to the highest and lowest measured area in *shGFP* (*n* = 86), *shAlkbh1* (*n* = 63), rescue (*n* = 64), and mutant‐rescue (*n* = 66) where *n* = nuclei numbers. Central band shows the mean, the boxes show lower and upper quartiles, and whiskers show minimum and maximum data values. Data information: In (A), scale bar is equal to 20 μm. In (B), *P < 0.05, **P < 0.01, ***P < 0.005, and n.s., not significant, unpaired two‐tailed Student's *t*‐test. Source data are available online for this figure.

**Figure EV2:**
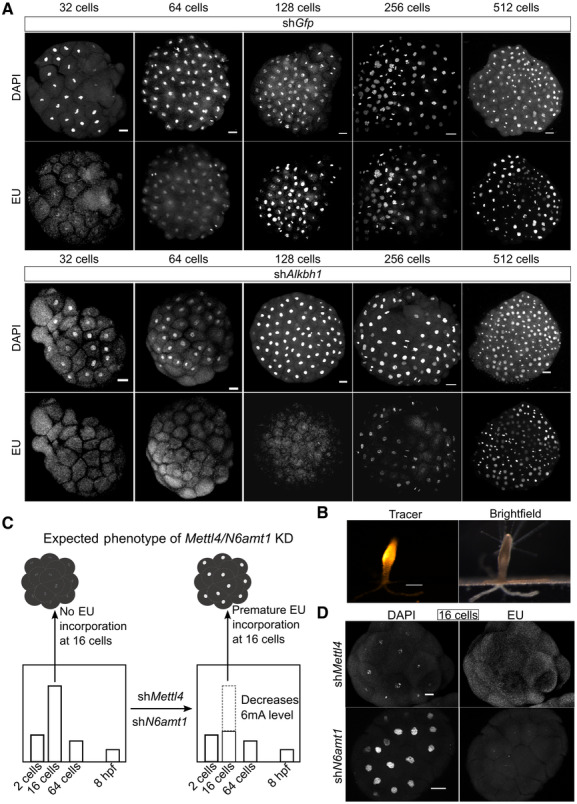
Alkbh1‐KD not lethal and putative methyltransferases‐KD Alkbh1 knockdown does not inhibit EU incorporation in 256 and 512‐cell embryos.sh*Alkbh1* injected embryo develops into a normal polyp.Experiment setup. Knockdown of *Metll4/N6amt1* would be expected to result in premature ZGA if these enzymes were acting as 6mA methyltransferases.
*Mettl4* and *N6amt1* knockdown does result in premature ZGA, suggesting that they do not act as 6mA methyltransferases. Data information: Scale bars: 20 μm. Source data are available online for this figure.

The occurrence of a major wave of ZGA immediately following 6mA clearance at the 64‐cell stage prompted us to explore a possible functional link between these two phenomena. To examine this potential link, we injected *shAlkbh1* into zygotes to target *Alkbh1* mRNA and impede 6mA clearance. We then assessed zygotic transcription at the 64‐cell stage by EU incorporation. We found that lowering Alkbh1 activity and the resulting elevated level of 6mA at the 64‐cell stage (Fig 2) caused major wave ZGA to be delayed by three cell cycles, commencing at the 256/512‐cell stage instead of at the 64‐cell stage as in untreated and *shGFP*‐injected embryos (Figs 4 and EV2A). Injecting shRNA‐resistant *Alkbh1* mRNA rescued the phenotype, while mutated, catalytically inactive *Alkbh1* mRNA was ineffective (Fig 4A and B). This suggests that the catalytic activity of Alkbh1 is required for 6mA clearance (Fig 4C).

The late ZGA suggests that 6mA interferes with transcription, consistent with a previous study showing that genomic 6mA causes transcriptional pausing by stalling RNA polymerase II (Wang *et al*, 2017). The late recommencement of zygotic transcription in *Alkbh1‐*knockdown embryos could have been enabled by 6mA dilution after DNA replication, assuming that 6mA incorporation was limited to occurring primarily in single‐to 16‐cell embryos. Delayed ZGA in *Alkbh1‐*knockdown embryos caused no visible long‐term defects; the embryos developed normally to planula larvae and successfully metamorphosed to primary polyps (Fig EV2B).

### The source of 6mA in the embryonic genome

To address how 6mA is incorporated into the *Hydractinia* genome between the 2‐ and 16‐cell stages, we initially focused on *Mettl4 and N6amt1*, homologs of both of which have been proposed to function as 6mA methyltransferases in other animals (Greer *et al*, 2015; Xiao *et al*, 2018). The *Hydractinia* genome encodes one copy of each of the genes (Appendix Fig S5). If one of these genes (*N6amt1* or *Mettl4*) functioned as a 6mA methyltransferase, their downregulation would be expected to cause premature ZGA due to the absence of 6mA at the 16‐cell stage (Fig EV2C). However, downregulation of *Metll4* and *N6amt1* using shRNA did not result in premature ZGA at 16‐cell embryos (Fig EV2D). Of note, *Hydractinia* and other animals' N6AMT1 proteins contain no clear nuclear localization signal (Table EV1). The likely inability of N6amt1 to act on nuclear DNA is inconsistent with a role as 6mA methyltransferases. Therefore, we propose that these genes do not act as 6mA methyltransferases, consistent with the other reports (Yang *et al*, 2004; Ratel *et al*, 2006; Xie *et al*, 2018; Woodcock *et al*, 2019; Chen *et al*, 2020; Gu *et al*, 2020; Liu *et al*, 2021; Luo *et al*, 2022).

**Figure 5:**
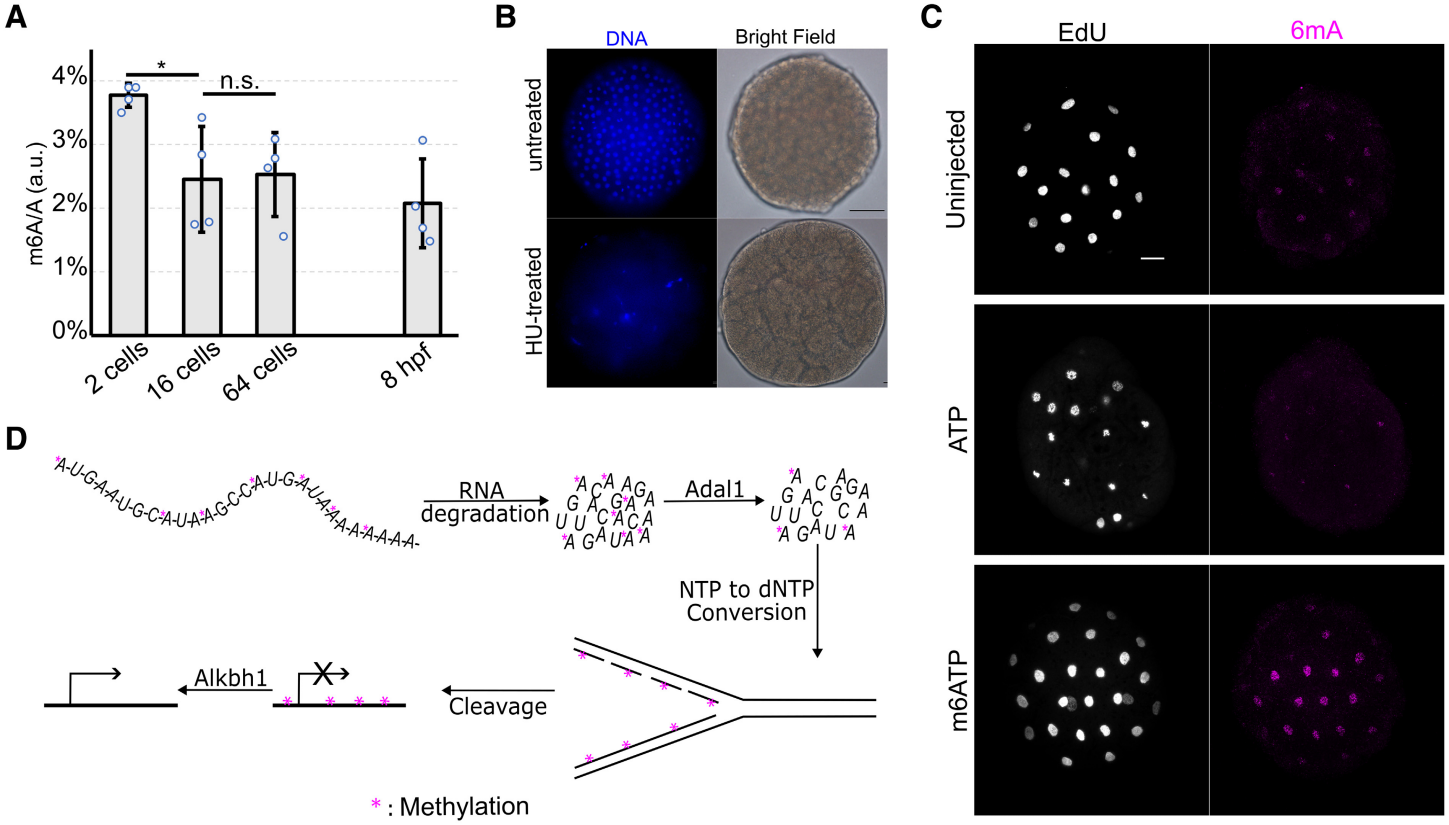
Maternal RNA recycling hypothesis and the evidence supporting it Rapid decline of m6A‐marked maternal RNA occurs between the 2‐ to 16‐cell stages, analyzed by UHPLC‐QQQ of m6A/A (% in arbitrary unit) from four *Hydractinia* developmental stages.Replication stall at 8–16 nuclei following hydroxyurea treatment. The control shows a normal number of nuclei at the same developmental stage as treated one.Whole‐mount immunofluorescence of anti‐6mA in 32‐cell embryos upon injection of ATP and m6ATP at 20 mM (see text). The uninjected control shows a normal level of anti‐6mA at 32‐cell embryos.Model displaying the random incorporation of 6mA into the zygotic genome through the RNA recycling hypothesis. Methylated adenines are removed by Alkbh1 at the DNA level and by Adal1 at the nucleotide level to allow timely zygotic transcription. Data information: In (A), *P < 0.05, **P < 0.01, ***P < 0.005, and n.s., not significant, unpaired two‐tailed Student's *t*‐test. The number of biological replicates used for each sample is indicated by the amount of data points represented in the graph. Error bars indicate standard deviation. In (B and C), scale bar is equal to 20 μm. Source data are available online for this figure.

**Figure EV3:**
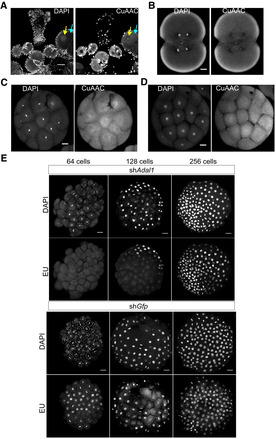
Transfer of nucleotides from maternal RNA to zygotic DNA and *Adal*‐KD EU incorporation into nascent maternal RNA by a gravid female shown by CuAAC‐Alexa 488 reaction in the cytosol (cyan arrow) and nucleolus but not in the nucleus (yellow arrow).Cytosolic maternal RNA at 2/4‐cell stage embryo.CuAAC‐Alexa 488 reaction stains the zygotic DNA in a 16‐cell stage embryo.Negative control displays no staining in a 16‐cell stage embryo.Embryos injected with sh*Adal1* display no EU incorporation at 64‐cell embryos, low at 128‐cell, and high incorporation at 256‐cell embryos. Data information: Scale bar in (A): 50 μm. Scale bar in (B–E): 20 μm. Source data are available online for this figure.

A possible alternative source for methylated adenosine is m6A‐marked RNA. In animals, maternal transcripts are degraded prior to ZGA (Varnum & Wormington, 1990; Chen *et al*, 2019), with m6A acting as a degradation mark (Ivanova *et al*, 2017; Zhao *et al*, 2017). Methylated adenine from degraded maternal RNA could be recycled through conversion to dNTP by ribonucleotide reductase (RNR), fueling methylated DNA synthesis during embryonic cleavage. To address this possible scenario, we have performed HPLC–MS/MS experiments and found that m6A‐marked RNA levels are indeed rapidly decreased (~ 35%) between the 2‐cell and the 16‐cell stages in *Hydractinia* embryos (Fig 5A). Next, we continuously inhibited RNR by hydroxyurea, starting with zygotes. This treatment resulted in stalled replication at the 8‐cell stage (Fig 5B), indicating the depletion of maternally provided dNTPs and the requirement for NTP‐dNTP conversion prior to this stage. These two pieces of evidence may indicate that rapid degradation of m6A‐marked RNAs provides methylated A nucleotides that can be converted into dNTPs by RNR before the 8‐cell stage to allow DNA replication. However, the decreasing levels of m6A‐marked RNAs in early embryos could also be due to demethylation rather than degradation. Thus, we labeled gravid females with EU, allowed them to spawn, and fertilized the eggs. The resulting embryos had the signal in their nuclei at the 16‐cell stage (Fig EV3A–D), consistent with maternal RNA being recycled and incorporated into the embryonic genome. Next, we injected m6ATP into zygotes and observed higher level of 6mA in the nuclei of 32‐cell embryos compared with ATP‐injected or ATP‐untreated embryos (Fig 5C), consistent with studies done in mammalian cells, showing that m6A ribonucleotides can be converted to 6mA deoxyribonucleotides and incorporated into the genome through replication that is conserved in eukaryotes (Musheev *et al*, 2020; Liu *et al*, 2021). Finally, we found that knocking down the *Hydractinia Adal1*, homologs of which convert m6A ribonucleotides into inosine in plants and mammals (Chen *et al*, 2018b; Chen *et al*, 2023), also delayed EU incorporation beyond the 64‐cell stage (Fig EV3E). Since Adal1 only acts on monomeric m6A, this experiment provides further evidence for the RNA degradation scenario over demethylation.

Taken together, we conclude that m6A‐marked RNAs are degraded into nucleotide form. Some but not all m6As are cleared by Adal1. Methylated and unmethylated NTPs are converted to dNTPs by RNR and incorporated into the genome during rapid cleavage. Genomic 6mA is cleared by Alkbh1 before the 64‐cell stage to allow transcription (Fig 5D).

## Discussion

6mA is a random and passively incorporated DNA modification that interferes with transcription but does not function epigenetically. Our data are consistent with m6A from degraded RNA being a source of methylated nucleotides for the zygotic genome (Fig 5D). The combined act of removal of 6mA from the genome by Alkbh1 and of m6A from the nucleotide pools by Adal1 during and after the 16‐cell stage facilitates timely ZGA in *Hydractinia* embryos (Fig 5D).

An inverse correlation between zygotic transcription and 6mA during early embryogenesis can be inferred from studies on zebrafish and *Drosophila* (reviewed in Bochtler & Fernandes, 2021). In these animals, high rates of m6A‐marked RNA degradation are followed by high peaks of genomic 6mA that is cleared before ZGA (Zhao *et al*, 2017; Wu *et al*, 2022; Zhang *et al*, 2022), suggesting that our findings in *Hydractinia* also apply to other animals.

Rapid degradation of accumulated m6A‐marked RNA serves as a metabolic “cheap” source of nucleotides for rapid successive cell divisions in embryogenesis. This mechanism, however, generates a by‐product of 6mA in the genomes of these cells. Since genomic 6mA interferes with RNA polymerase activity, “cleaning” mechanisms by Adal1 on nucleoside level and Alkbh1 on the DNA level may have evolved to allow timely zygotic transcription in animal embryos.

In single‐cell eukaryotes, 6mA is found in a palindromic ApT context (Fu *et al*, 2015; Mondo *et al*, 2017; Wang *et al*, 2017; Chen *et al*, 2018a), strongly associated with transcription start sites in active genes, indicating a heritable epigenetic function. Bacterial 6mA has a different role, but is also found in an ApT context (Lahue *et al*, 1987). This contrasts with the random distribution of 6mA in the genome of *Hydractinia* embryos. Furthermore, 6mA methyltransferases found in bacteria, ciliates, and oomycetes are distinct from the putative animal 6mA methyltransferases (Haagmans & van Der Woude, 2000; Chen *et al*, 2018a; Beh *et al*, 2019; Wang *et al*, 2019). Therefore, a role for 6mA as epigenetic mark in protists and bacteria is probably a specific trait in these lineages that is not shared with animals.

## Materials and Methods

### Animal husbandry and embryos collection

Clones of Hydractinia, male (291–10) and female (295–8, 295–6) strains, were grown as described previously (Frank *et al*, 2020). Zygotes were collected and immediately cleaned with sterile‐filtered seawater. For manipulation and injection purposes, the zygotes were incubated in ice‐cold condition to delay cleavages (Chrysostomou *et al*, 2022).

### DNA extraction

DNA was extracted from *Hydractinia* embryos and adult specimens (see Appendix Table S2) by proteinase‐K (200 μg) incubation at 55°C for 2 h in buffered SDS solution (NaCl 0.1 M, Tris 10 mM, EDTA 25 mM, SDS 0.5%). One volume of Phenol‐Chloroform (1:1, v/v) was used to separate the DNA from the proteins. The DNA was then precipitated by sodium acetate–ethanol (0.1 and 3 v, respectively), washed with 70% ethanol, and dissolved in nuclease‐free water (adapted from Sambrook & Russell, 2001). Following RNaseA (ThermoScientific #EN0531) and RNaseT1 (ThermoScientific #EN0541) treatment, the DNA was further purified using a standard column‐based purification protocol (Escobar & Hunt, 2017). The purified DNA was then assessed by UV–Vis spectrophotometer, Qubit dsDNA‐BR (ThermoScientific # Q32850) and Qubit RNA‐HS assay (ThermoScientific # Q32852). Only DNA solutions with undetectable levels of RNA by Qubit RNA‐HS assay were used.

### UHPLC‐QQQ and UHPLC‐QTRAP for determination of 6mA levels

A total of 2 μg of DNA (all standards and samples) were prepared for digestion. For UHPLC‐QTRAP, one picomole of ^3^D_1_‐6mA was added to the solutions as internal standard. External standards were prepared from serial dilution of modified oligonucleotide (5′‐^**6m**^ **A**TCGATCG‐3′) solutions. A total of 2 μg variable standard solutions were prepared from the calculated combination of the above‐modified oligonucleotide and an unmodified oligonucleotide (5′‐GGGCAGTACACAGACTATGTTG‐3′) solutions. DNA solutions were then denatured at 100°C for 5 min, chilled on ice for 2 min, and digested following a protocol described before (Greer *et al*, 2015). After centrifugal ultrafiltration (MW cutoff 3 kDa, Amicon, Millipore #UFC500396), the nucleotide solutions were assessed by Nanodrop and Qubit dsDNA‐HS assay. The total amount of DNA is expected to be equal by Nanodrop measurement before and after digestion. QUBIT dsDNA‐HS was used to confirm zero dsDNA in the solutions. The digested DNA solutions (samples and standards) were then injected in 2 μl of volume into an Agilent 1100 HPLC system coupled to a triple quadrupole (QQQ) 6460 mass spectrometer (Agilent Technologies Ltd, Cork, Ireland), or injected in 6 μl volume into and an Agilent 1260 HPLC system coupled to an SciEx 4500 QTrap. Analyte separation by liquid chromatography was carried out using reverse‐phase Zorbax SB‐C18 column (2.1 mm width × 50 mm length; 1.8 μm particles), flow rate 250 μl/min using mobile phase A (0.1% formic acid solutions in water) and mobile phase B (0.1% formic acid in acetonitrile). To detect the analytes, the QQQ and the QTRAP modes were set to positive electrospray ionization and selective multiple reaction monitoring (MRM). Nucleosides were identified using the nucleoside precursor (parent) ion to product (daughter) ion mass transitions; dC (228.1/112.1), dA (252.1/136.1), 6mA (266.1/150.1) and ^3^D_1_‐6mA (269.1/153.1). Mol of dA and 6mA from the QQQ was interpolated from the standard curve rendered from serial dilution of digested external standards. To address potential ionization suppression effects of dA in our QTRAP setup, 4 picomol spiked‐in dA into blank (water) and biological samples of 64‐cell embryos were performed. We found no statistically significant differences (*t*‐test *P*‐value = 0.87) in the level spiked‐in dA detected either from blank or from samples, indicating no significant ionization suppression (Appendix Fig S1C). The mol 6mA from QTRAP was calculated following the previously reported guideline using the direct comparison to the ^3^D_1_‐6mA internal standards (Traube *et al*, 2019). The 6mA/dA ratio was calculated as the mol of 6mA per total mol of deoxyadenosine (dA + 6mA).

### Dot‐blot

Dot‐blotting was performed on 200 ng of RNA‐free genomic DNA solutions and standard solutions from unmodified and modified oligonucleotides (0% and 0.1% 6mA/dA) as described (Greer *et al*, 2015) on Amersham Hybond‐N+ membrane (GE #RPN119B) using anti‐6mA antibody (Synaptic System #202003).

### EU incorporation

Embryos were rinsed in filtered seawater and incubated in 1 mM EU (Jena Bioscience #CLK‐N002) for 45 min before being fixed in PFA + Ac solution (paraformaldehyde 4 and 0.5% freshly added glacial acetic acid (Fernández & Fuentes, 2013) on a rocker at room temperature for 1 h. The embryos were then rinsed in 200 mM glycine for 15 min and then permeabilized by 1× PBS and 0.5% Triton X‐100 (PTx) (3 × 15 min). The embryos were then rinsed in 1 ml of 2 M HCl for 45 min to denature the DNA as antigen retrieval step. The HCl was neutralized, and embryos were washed with 1 ml 100 mM Tris–HCl pH 8.0 for 2 × 15 min. The embryos were then rinsed in 1 ml block‐i1 solution (3% BSA (MP Biomedicals #11444296) and 0.25% Triton X‐100 (MP Biomedicals #11471632) in 1× PBS) overnight at 4°C on a rocker, followed by CuAAC reaction. Nucleotide penetration into the 16‐cell stage embryo was verified by EdU (Appendix Fig S4B).

### CuAAC reaction

Ethynyl groups in EU/EdU act as the alkyne, which can react with fluorophore‐tagged azide through The Cu(I)‐catalyzed alkyne‐azide chemistry (CuAAC) reaction (Presolski *et al*, 2011). The CuAAC solutions (Jena Bioscience #CLK‐074) were prepared freshly (Alexafluor488‐picolylazides 2 μM, CuSO_4_ 1 mM, THPTA 5 mM, and Na‐Ascorbate 100 mM, in sodium phosphate buffer).

Next, embryos in the block‐i1 solution brought back to room temperature. The block‐i1 solution was then replaced with 500 μl CuAAC solutions and incubated on the rocker for at least 45 min in the dark at room temperature followed by two PTx washes (15 min each). The DNA was then stained with DAPI and the embryos mounted for imaging.

### Whole‐mount immunofluorescence

Embryos were incubated in 10 μM EdU (Jena Bioscience # CLK‐N001) ~ 45 min before fixation with PAGA‐T (20% PEG 6000 [Sigma #81260], 4% Glycerol [Sigma #G5516], 2.5% Acetic Acid, 56% Ethanol in 100 mM Tris–HCl pH 6.0 [Invitrogen # 15568025; Zanini *et al*, 2012] for 1 h at 4°C). The fixed embryos were then washed with 1:3 mixture of PAGA‐T and PTx. Permeabilization was done by an additional wash of the fixed embryos with PTx for 15 min on a rocker at room temperature for three times.

Samples were then treated with 1:50 RNase solution (mixture of RNaseA, T1 and H [20 mg/ml, 1,000 and 10 U/μl, respectively]) and/or DNase (2 U/μl, NEB #M0303) at 37°C overnight. After one PBS wash, the embryos were rinsed in 1 ml of HCl 2M for 45 min to denature the DNA as antigen retrieval step. The HCl was neutralized, and embryos were washed with 1 ml 100 mM Tris–HCl pH 8.0 for 2 × 15 min. The embryos were then rinsed in 1 ml block‐i1 solution for 1.5 h at room temperature on a rocker.

Next, the block‐i1 solution was replaced with 500 μl CuAAC solutions (described above) and then incubated on the rocker for at least 45 min in the dark and room temperature followed by two PTx washes. The fixed embryos were rinsed in 1 ml block‐i1 solution (3% BSA in PTx) overnight at 4°C before replaced with 200 μl of the Rabbit anti‐6mA antibody solutions (diluted 1:8000 in block‐i1, Synaptic Systems #202003) for 1 h at room temperature. Then, the fixed embryos were washed in 1× PBS for 2 × 15 min and then rinsed in 400 μl block‐i2 solution (5% goat serum [ThermoFisher #16210064] and 3% BSA in PTx) for 2 h at room temperature. Then, embryos were soaked in anti‐rabbit Alexafluor 594 antibody (1:2,000 in block‐i2) for 1 h at room temperature. Next, the embryos were washed three times with PBS and mounted for confocal microscope imaging.

### Image preparation and quantification

The mounted embryos were imaged by a confocal laser scanning microscope (Olympus FV1000). Positive and negative control samples were used to calibrate the confocal setup. This setup was used when images taken from sample slides on the same day of image acquisition.

Images were imported to ImageJ software (Schneider *et al*, 2012). Nuclei were the region of interest (ROI); thus, we used the threshold approach to select nuclear regions from the DAPI channel as the ROI. These ROIs were then used to measure the mean fluorescence intensity (MFI) and corrected to the background ROI following the standard quantitation method (Shihan *et al*, 2021).

To compare the images, we normalized all MFI of the images to be compared by defining the highest MFI in the population as 1 and the lowest MFI value as 0; thus, normalized MFI values were calculated using the following equation:

$$\text{normalized}\;\mathrm{MFI}=\frac{\text{sample}\;\mathrm{MFI}-\text{lowest}\;\mathrm{MFI}}{\text{highest}\;\mathrm{MFI}-\text{lowest}\;\mathrm{MFI}}$$

The normalized MFI was visualized using the online software at https://huygens.science.uva.nl/PlotsOfData/ (Postma & Goedhart, 2019).

### SMRT‐seq

Raw PacBio reads from adult polyps were provided by the NIH Intramural Sequencing Center (NISC) in fastq, bax.h5, and bash.h5 format. These files were converted to BAM format using bax2bam (SMRT Analysis; https://www.pacb.com/support/software‐downloads/). Raw PacBio reads for 16‐cell and 64‐cell samples were provided in BAM format. BAM files for all three samples were aligned to the assembled genome with pbalign (https://www.pacb.com/support/software-downloads/) in base modification identification mode, with the command‐line version using default parameters and BAM formatted output. IpdSummary of SMRT Analysis (https://www.pacb.com/support/software‐downloads/) was used to identify 6mA (using default options, with *P*‐value 0.001, methyl fraction calculation, 6mA identification, and GFF output). The GFF output was then imported to Geneious for manual analysis. We achieved the recommended coverage (Zhu *et al*, 2018) in all datasets (16‐cell, 64‐cell, and adult polyps at 73×, 117×, and 120×, respectively).

Afterwards, 6mAs were filtered to remove those with IPD ratio below 3.0 (Zhu *et al*, 2018). Analysis of methylation motifs was performed with two different strategies. First, possible motifs were determined with MotifMaker using default options (SMRT Analysis; https://www.pacb.com/support/software‐downloads/). To further confirm the lack of motif identification, all 6mA loci were separated into 20 groups based on their percent occurrence (in 5% intervals), and the regions 3 bp upstream and downstream of each 6mA were extracted. MEME‐ChIP (Machanick & Bailey, 2011) was then used to identify consensus sequence in each group.

### RNA extraction and m6A detection

Total RNAs were extracted from embryos of 2–4 cell, 16–32 cell, 64–128 cell stages, and 24‐h postfertilization using TRIzol solution (ThermoScientific #15596026) followed by RNA binding onto columns (EpochLifeScience #1940) and on‐column DNA digestion (Qiagen #79254). RNA was then eluted with nuclease‐free water, assessed with a Qubit RNA‐HS assay and electrophoresed along with RNA loading dyes (ThermoScientific #R0641) in denaturing formaldehyde agarose gel before visualization under UV illumination. After RNase A/T1 overnight digestion and ultrafiltration, purified high‐quality ribonucleosides were used to detect m6A using UHPLC‐QQQ with MRM of A (268.1/152.1) and m6A (282.1/166.1).

### Multiple sequence alignment (MSA) and phylogenetic tree inferences

Sequences of Alkbh1 (Uniprot ID: P0CB42), N6AMT1 (Q9Y5N5), Alkbh4 (Q8MNT9), and Mettl4 (Q09956) were used as queries to retrieve orthologous sequences from a *Hydractinia symbiolongicarpus* transcriptome using tblastn. We retrieved the sequences of the respective homologs from each species from the UniProt database (https://www.uniprot.org) and the Ensembl omics database (https://metazoa.ensembl.org/), which were then imported into Geneious Prime 2019.0.4. We retrieved the homologous sequences of *Mnemiopsis leidyi* (NHGRI), *Hydra vulgaris* (NHGRI), *Hydractinia echinata* (NHGRI), *Saccoglossus kowalevskii* (OIST), and *Acropora digitifera* (OIST) from their specific respective database. Sequences were aligned in Geneious using MAFFT with the E‐INS‐i algorithm, a JTT PAM100 scoring matrix, and a gap penalty of 1.53 (Katoh & Standley, 2013).

The phylogenetic trees were built as a combination of three independent inferences from multiple sequence alignments. First, a phylogenetic tree was built by RAxML 8.2.11 (Stamatakis, 2014) using the GAMMA LG protein model (default), rapid bootstrapping (10,000 replicates) and searching for best‐scoring maximum likelihood tree algorithm. Second, a Bayesian phylogenetic tree was produced using MrBayes v.3.2.2 (Ronquist *et al*, 2012). The program was run using a fixed WAG substitution model (recommended by MrBayes trial with the respective MSA with 500 generations and sampled every 50^th^ generation) with gamma‐distributed rate variation across sites (“lset rates = gamma”) with four chains for 4 million generations. The run was sampled every 500^th^ generation and analyzed with a 20% burn‐in. These two methods of phylogenetic tree inference are available in Geneious. The consensus tree from maximum likelihood analysis was then exported and manually edited in InkScape to mark the nodes with support values as annotated from the two different methods of phylogenetic inference with greyscale dots.

### Localization signal

Sequences from *Hydractinia symbiolongicarpus* and *Homo sapiens* homologous proteins were analyzed for nuclear localization signals by cNLS Mapper (Kosugi *et al*, 2009), by NLSdb (Bernhofer *et al*, 2018) and for protein sorting in general by Wolf Psort (Horton *et al*, 2007). The results retrieved and imported to Microsoft Excel for data visualization and presented as Table EV1.

### *Alkbh1* knockdown and rescue experiment

Short hairpin RNA was designed according to a previous report (DuBuc *et al*, 2020). T7 IVT kit was used to synthesize mRNA to confirm the efficacies of sh*Alkbh1* by adding the endogenous target of *Alkbh1* sequences at the 3′ of *mScarlet* coding sequence. Rescue *Alkbh1* mRNA was designed by introducing four silent mutations, T861C, A864G, C865T, and A867G to render it unrecognizable by sh*Alkbh1*. Catalytically inactive rescue *Alkbh1* mRNA was designed by introducing four mutations, C631G, A632C, A638C, and C639A to render H211A and D213A as reported previously (Liu *et al*, 2016b; Tian *et al*, 2020) (Appendix Table S1).

### Microinjection

Fertilized eggs were transferred to a Petri dish coated with a 200‐micron Nitex mesh screen. Zygotes are 180–200 microns and settled in the holes. Cells were injected, prior to first cleavage, using a Narishige IM 300 microinjection system. To delay cleavage, zygotes were stored on ice prior to injection. ATP and m6ATP were injected at 20 mM.

### Electroporation

Zygotes were rigorously cleaned with filtered‐sterile seawater and then electroporated to insert sh*Alkbh1* into the cell following the previously described protocol (Quiroga‐Artigas *et al*, 2020) with Ficoll replaced by 1.54 M Mannitol. Next, zygotes were immediately transferred into a large volume of filtered‐sterile seawater in glass Petri dish and left at room temperature for 1 h before further cleaning and then used for DNA extraction, DNA digestion, and UHPLC‐QTRAP protocols as described above.

### Hydroxyurea treatment

Cleaned 2‐cell stage embryos were incubated in seawater with 10 mM Hydroxyurea (HU) and collected at the 256/512‐cell stage, while the negative control embryos were incubated only in seawater. Both the HU‐treated embryos and negative control were soaked in Hoechst‐33,342 (diluted 1:2,000 in seawater) for 15 min and then mounted for image acquisition on an epifluorescence microscope.

## Author contributions

**Febrimarsa:** Conceptualization; data curation; formal analysis; funding acquisition; validation; investigation; visualization; methodology; writing – original draft; writing – review and editing. **Sebastian Gornik:** Conceptualization; formal analysis; funding acquisition. **Sofia, N Barreira:** Data curation; formal analysis; visualization. **Miguel Salinas‐Saavedra:** Methodology. **Christine Schnitzler:** Resources; data curation; funding acquisition. **Andreas Baxevanis:** Resources; data curation. **Uri Frank:** Conceptualization; formal analysis; supervision; funding acquisition; investigation; writing – original draft; project administration; writing – review and editing.

## Disclosure and competing interests statement

The authors declare that they have no conflict of interest.

## Supporting information



Appendix

Expanded View Figures PDF

Table EV1

Dataset EV1

Source Data for Expanded View

PDF+

Source Data for Figure 1

Source Data for Figure 2

Source Data for Figure 3

Source Data for Figure 4

Source Data for Figure 5

## Data Availability

The data generated in the course of this study are publicly available through the *Hydractinia* Genome Project Portal (https://research.nhgri.nih.gov/hydractinia). Corresponding data are archived in the NCBI Sequence Read Archive (SRA) under BioProject PRJNA807936 (https://www.ncbi.nlm.nih.gov/bioproject/PRJNA807936).
