# Supplementary material for: Randomly incorporated genomic N6‐methyldeoxyadenosine delays zygotic transcription initiation in a cnidarian
Source: EMBO J. 2023 Jul 4;42(15):e112934. doi: 10.15252/embj.2022112934 (PMC10390872; doi:10.15252/embj.2022112934)
Supplement: Supplementary file 1 — Appendix [file EMBJ-42-e112934-s010.pdf]

Appendix Figure S1 2

Appendix Figure S2 3

Appendix Figure S3 4

Appendix Figure S4 5

Appendix Figure S5 6

Appendix Table S1. Details of key nucleoside/tide and amino acid sequences used. 7

Appendix Table S2. Samples-Lysis Buffer Ratio 8

Appendix Table S3. List of species and their abbreviation used in phylogenetic trees. 9

Appendix Figure S1

A

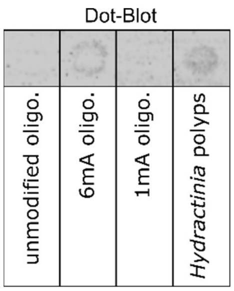

B

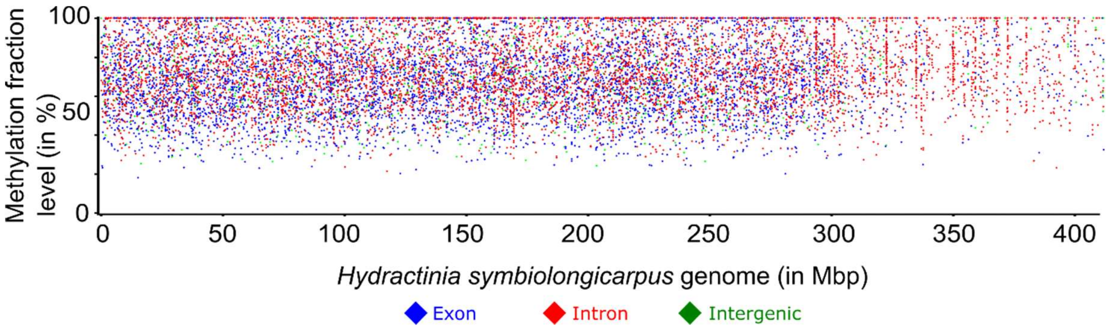

C

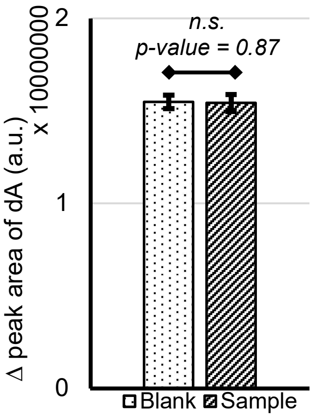

Appendix Figure S1. Detection and distribution of 6mA in the genome of *Hydractinia symbiolongicarpus*.

A. Anti-6mA specificity assay by dot-blot. Each spot contains 200 ng DNA. 6mA/1mA oligos were prepared at 0.1% of modified-A/dA.
B. Distribution of A sites that were detected to be methylated in the genomes of adult specimens, plotted against the percentage of SMRT-seq reads that showed methylation at each site.
C. Equal add-in dA level in blanks and sample solution indicates none to minimal ionisation suppression of dA detection in the pipeline of UHPLC-QTRAP.

Appendix Figure S2

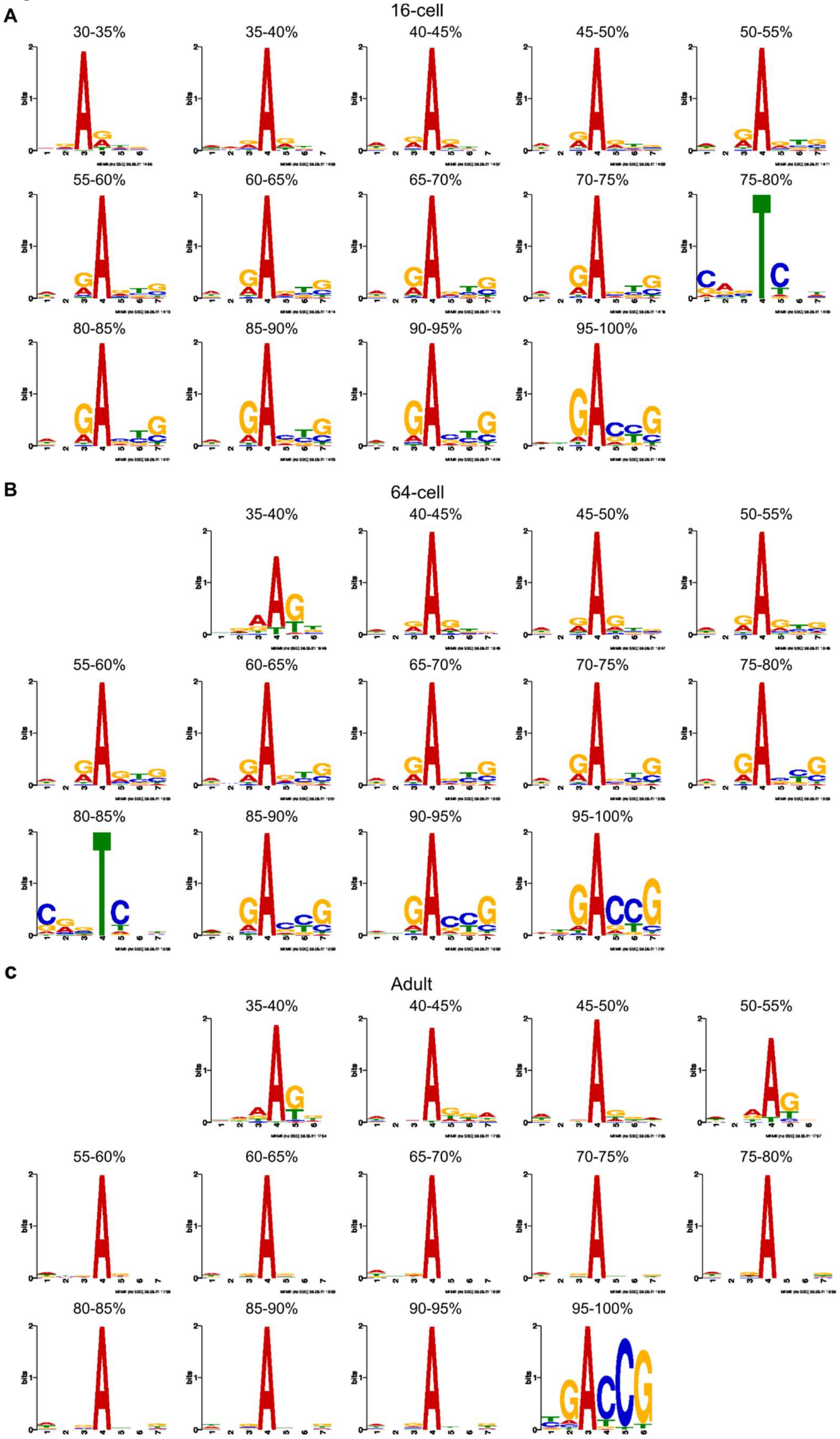

Appendix Figure S2. The consensus sequence generated by MEME-Chip of the methylated A loci in their respective methylation fraction.

Appendix Figure S3

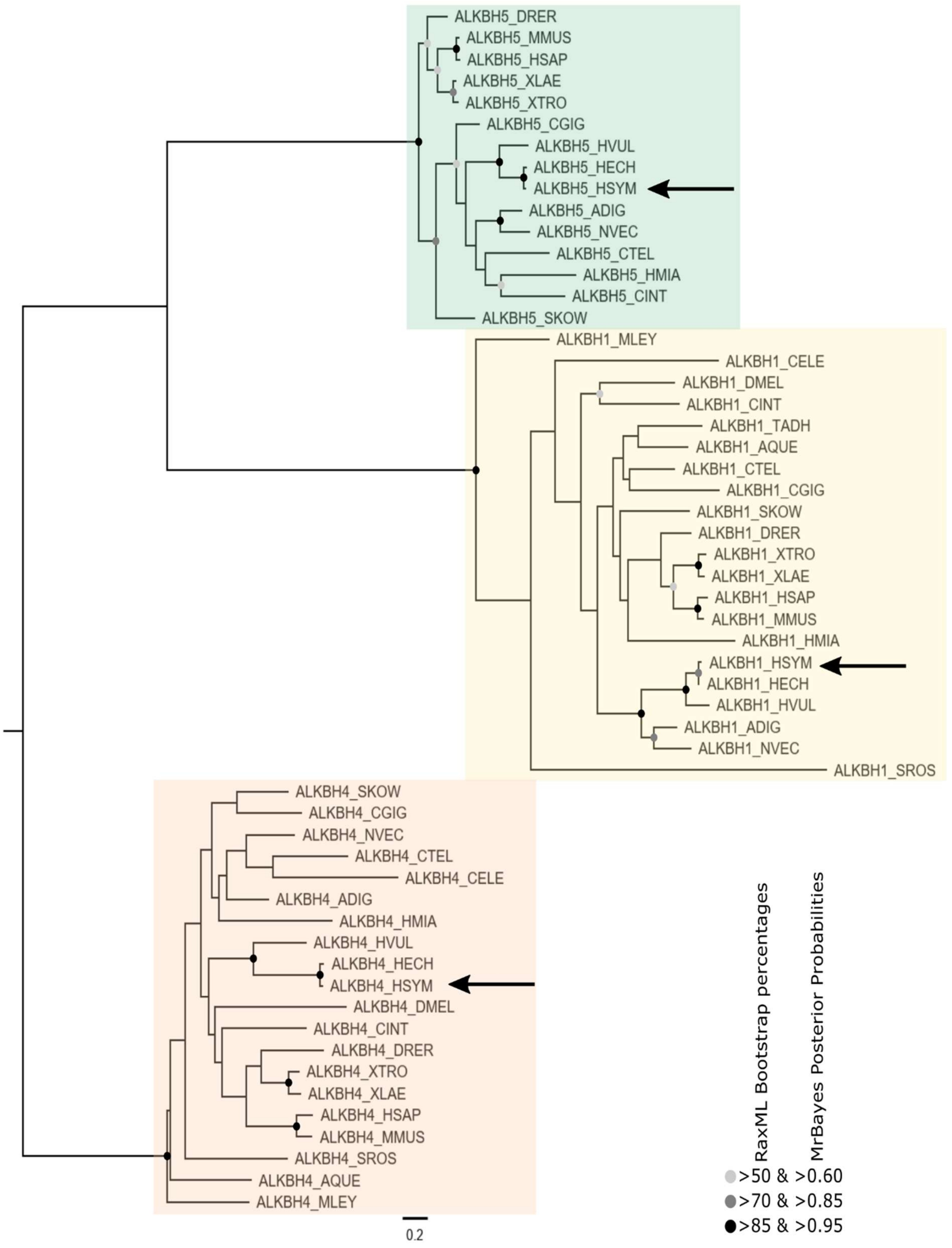

Appendix Figure S3. Molecular phylogeny of Alkbh proteins.
The trees represent a maximum likelihood phylogeny. Nodes supported by maximum likelihood bootstrap percentage and Bayesian inference posterior probability values are marked with greyscale circles as annotated. Alkbh homologs of *Hydractinia* are pointed by arrows. The abbreviation of the species are described in Appendix Table S3.

Appendix Figure S4

A

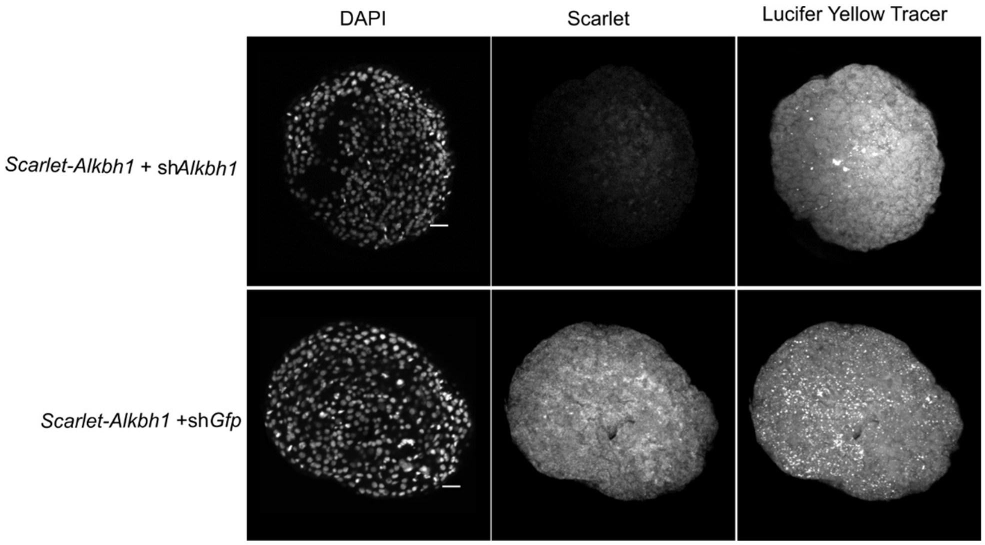

B

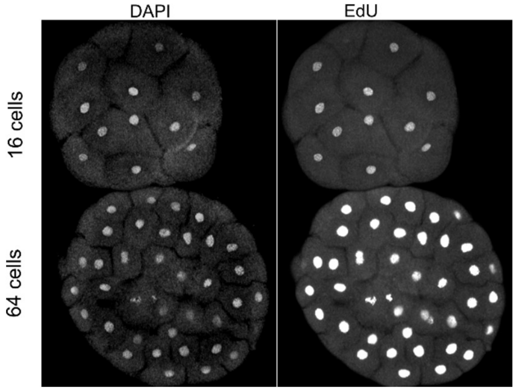

Appendix Figure S4. Image of control experiments.

A. Synthetic mRNA encoding mScarlet fluorescence protein followed by the target sequence of *Hydractinia* sh*Alkbh1* were co-injected (1 µg/µl) with sh*Alkbh1* and *shGFP* (each 500 ng/µl). Strong signals of mScarlett in *shGFP* co-injection but not on *shAlkbh1* is indicative of successful knockdown effect by *shAlkbh1* at 15 hpf embryos.
B. EdU is incorporated in 16- and 64-cell embryos as control experiment over EU incorporation at 16-cell embryos.

Data information: scale bars: 20 µm.

Appendix Figure S5

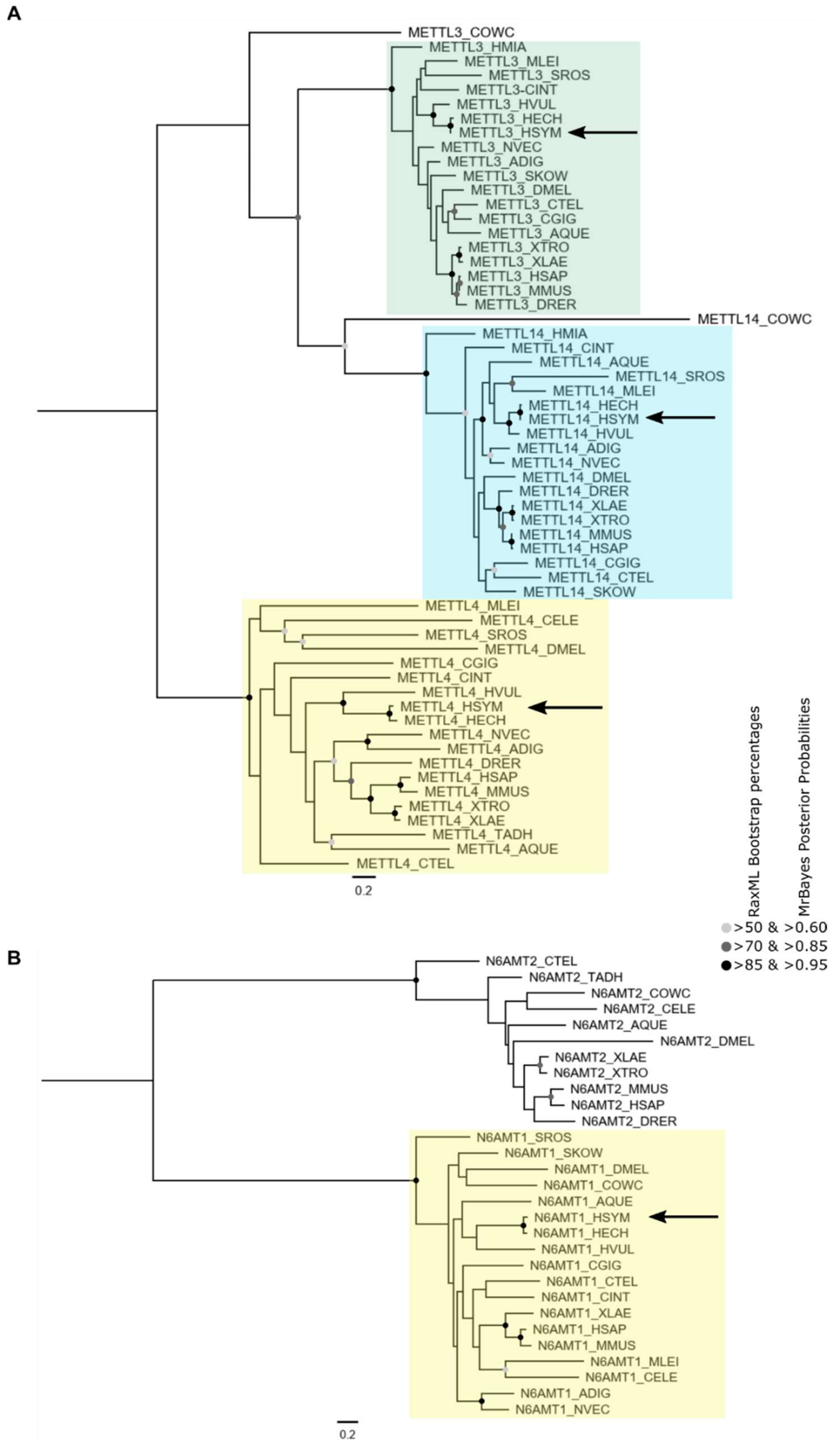

Appendix Figure S5. Molecular phylogeny of Mettl4 and N6amt proteins.
The trees represent a maximum likelihood phylogeny. The nodes with strong supports from maximum likelihood bootstrap percentages and Bayesian inference posterior probability are marked with a greyscale circle as annotated. Mettl4 and N6amt1 homologs of *Hydractinia* are pointed with arrows. The abbreviation of the species are described in Appendix Table S3.

Appendix Table S1. Details of key nucleoside/tide and amino acid sequences used.

Name	Sequences	
sh*Gfp*	GGAUGACG**C**GA**U**CU**G**CAAGACAAUUUACUUGUCUUGUAGUUCCCGUCAUC*UU*	
sh*Mettl4*	GAGAACUCUG**CU**A**G**GUACUCAAUUUACUUGAGUACGUAACAGAGUUCUC*UU*	
sh*N6amt1*	GCUUCAUAUG**G**CA**G**U**G**UUCAAAUUUACUUUGAAGAGUGGCAUAUGAAGC*UU*	
sh*Alkbh1*	GGCUCAUGUGCAGUAGUCACUAUUUACUAGUGACUAGUGGACAUGAGCC*UU*	
Endogenous target of *Alkbh1*	..GGCTCATGTCCACTAGTCACT..	
4-point mutation on rescue mRNA	..GGCTCATG**C**CC**GT**T**G**GTCACT..	
Catalytic Domain of *Alkbh1*	..GGCCATACTGACCAC..	[GHTDH]
Mutation on Catalytically inactive *Alkbh1*	..GGC**GC**TACTG**CA**CAC..	[G**A**T**A**H]

Mismatches/mutation, loop, *UU dinucleotide tail*, [amino acid sequence]

Appendix Table S2. Samples-Lysis Buffer Ratio

Samples	**Rough Estimation of specimens used for DNA extraction per sample**	**Unit**	**Volume of Lysis Buffer Sol. (µl)**
Sperm	5	Ejaculations off Male Polyps	200
2-cell	1000000	Embyros	500
16-cell	220000	Embyros	500
64-cell	60000	Embyros	300
8 hpf	12000	Embyros	200
24 hpf	12000	Embyros	200
Adult Specimen (polyps)	200	Polyps	200

Appendix Table S3. List of species and their abbreviation used in phylogenetic trees.

**Represented Phyla**	**Species**	**Abbrev.**
Choanoflagellata	*Salpingoeca rosetta*	SROS
Choanoflagellata	*Capsaspora owczarzaki*	COWC
Placozoa	*Trichoplax adhaerens*	TADH
Porifera	*Amphimedon queenslandica*	AQUE
Cnidaria: Anthozoa	*Nematostella vectensis*	NVEC
Cnidaria: Anthozoa	*Acropora digitifera*	ADIG
Cnidaria: Hydrozoa	*Hydra vulgaris*	HVUL
Cnidaria: Hydrozoa	*Hydractinia echinata*	HECH
Cnidaria: Hydrozoa	*Hydractinia symbiolongicarpus*	HSYM
Ctenophora	*Mnemiopsis leidyi*	MLEI
Xenacoelomorpha	*Hofstenia miamia*	HMIA
Ecdysozoa: Arthropoda	*Drosophila melanogaster*	DMEL
Ecdysozoa: Nematoda	*Caenorhabditis elegans*	CELE
Lophotrochozoa	*Capitella teleta*	CTEL
Lophotrochozoa	*Crassostrea gigas*	CGIG
Hemichordata	*Saccoglossus kowalevskii*	SKOW
Chordata: Tunicata	*Ciona intestinalis*	CINT
Chordata: Teleostei	*Danio rerio*	DRER
Chordata: Amphibia	*Xenopus laevis*	XLAE
Chordata: Amphibia	*Xenopus tropicalis*	XTRO
Chordata: Mammalia	*Mus musculus*	MMUS
Chordata: Mammalia	*Homo sapiens*	HSAP
