# Supplementary material for: Randomly incorporated genomic N6‐methyldeoxyadenosine delays zygotic transcription initiation in a cnidarian
Source: EMBO J. 2023 Jul 4;42(15):e112934. doi: 10.15252/embj.2022112934 (PMC10390872; doi:10.15252/embj.2022112934)
Supplement: Supplementary file 2 — Expanded View Figures PDF [file EMBJ-42-e112934-s011.pdf]

Expanded View Figures

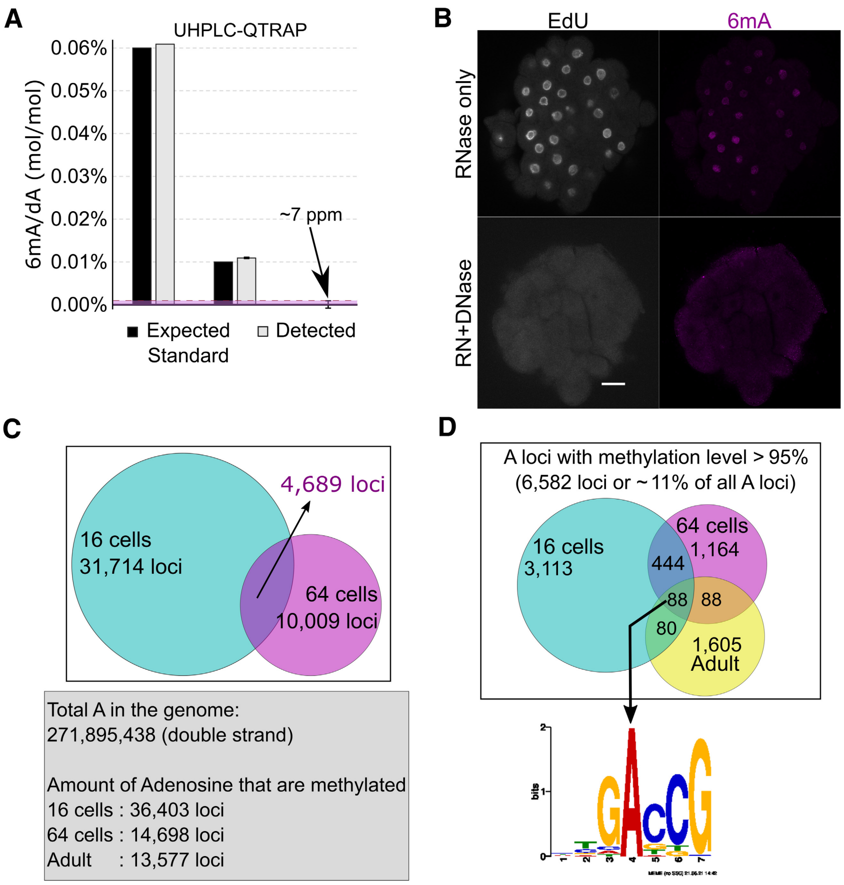

Figure EV1. Detection and distribution of 6mA in the genome of *Hydractinia symbiolongicarpus*.

A Detection of 6mA in technical replicates (n) of reference solutions (0%, $n = 3$; 0.01%, $n = 2$; 0.06%, $n = 1$) by UHPLC-QTRAP. Error bars indicate standard deviation.

B DNase but not RNase treatment can abolish the signal of anti-6mA immunofluorescence. Scale bars: 20 μm.

C Venn diagram displaying the overlapping methylated A sites between 16-cell and 64-cell genomes.

D Venn diagram displaying the overlapping A sites between three genome that are always methylated (> 95%) and the consensus sequence generated by MEME-Chip of the 88 overlapping methylated A-loci.

Source data are available online for this figure.

 © 2023 The Authors

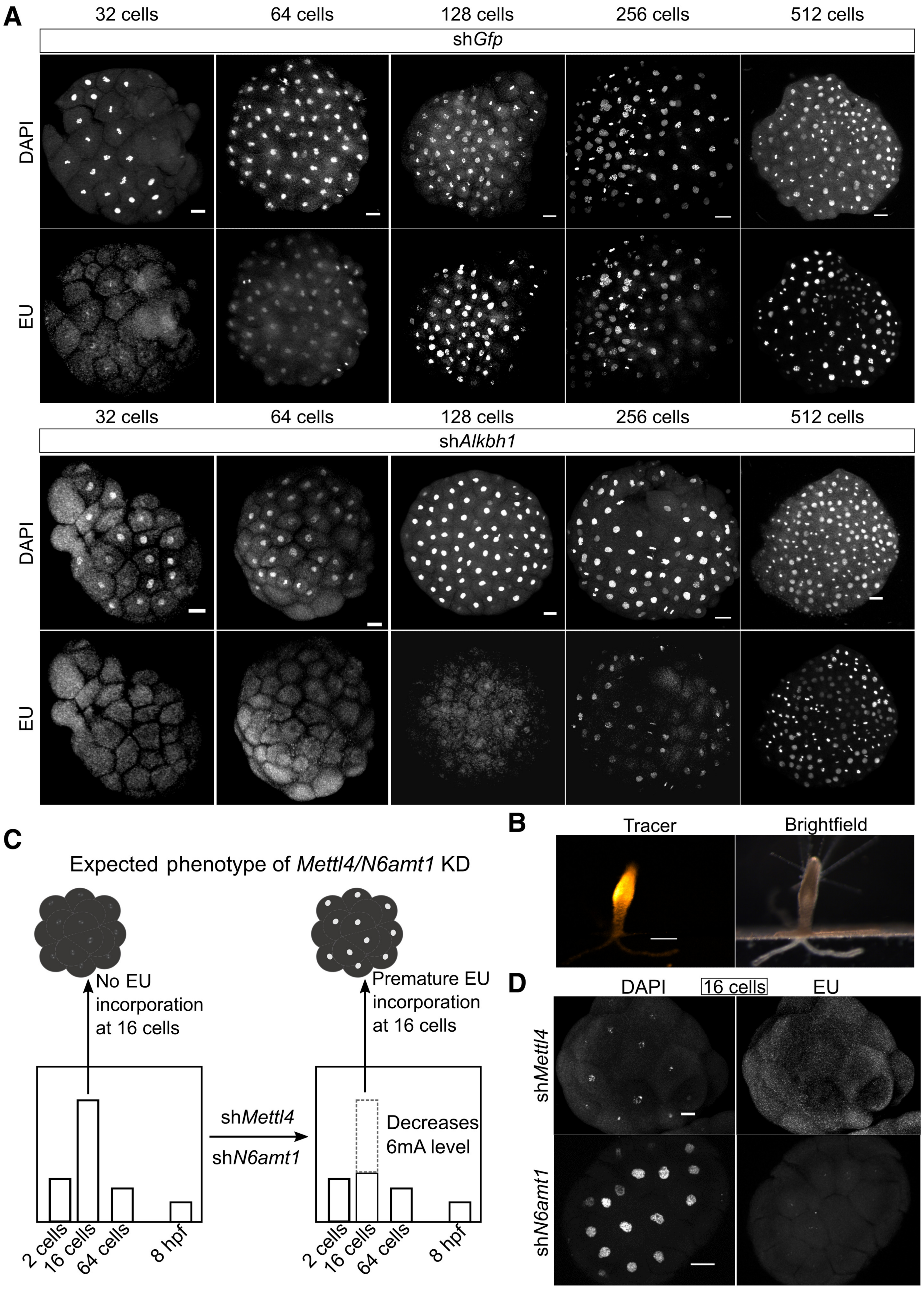

Figure EV2. Alkbh1-KD not lethal and putative methyltransferases-KD.

A Alkbh1 knockdown does not inhibit EU incorporation in 256 and 512-cell embryos.

B sh*Alkbh1* injected embryo develops into a normal polyp.

C Experiment setup. Knockdown of *Metll4/N6amt1* would be expected to result in premature ZGA if these enzymes were acting as 6mA methyltransferases.

D *Mettl4* and *N6amt1* knockdown does result in premature ZGA, suggesting that they do not act as 6mA methyltransferases.

Data information: Scale bars: 20 µm.

Source data are available online for this figure.

© 2023 The Authors

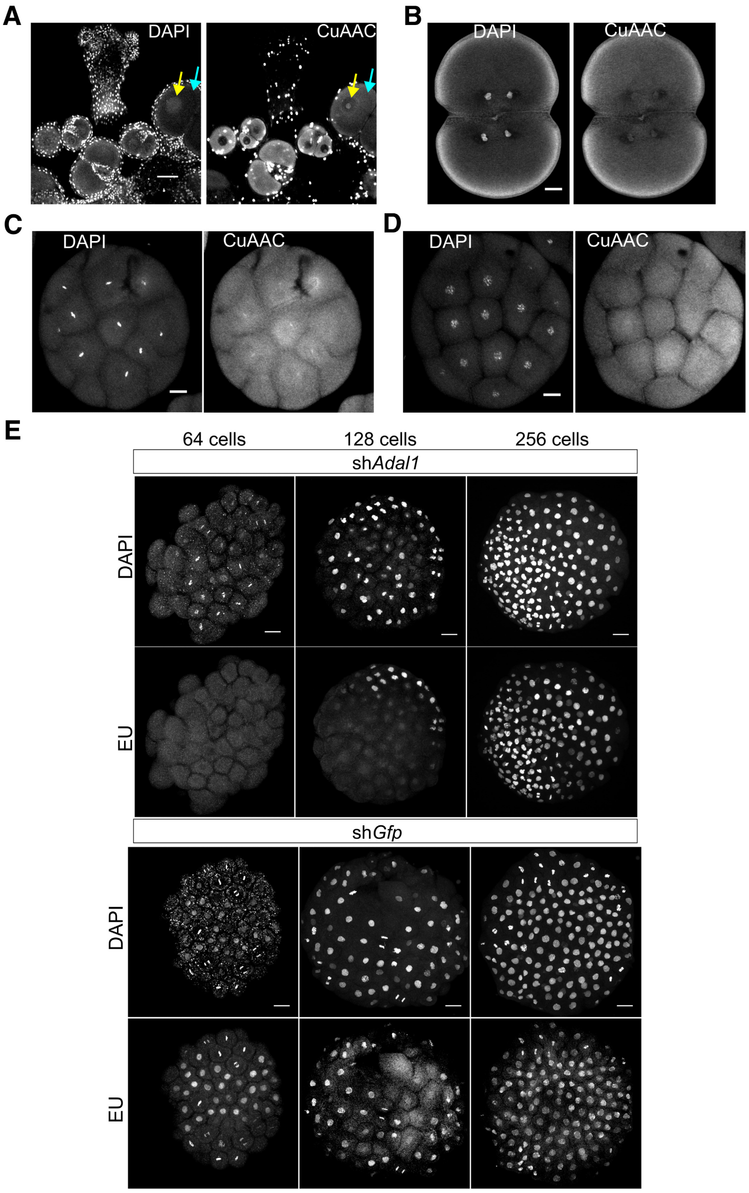

Figure EV3. Transfer of nucleotides from maternal RNA to zygotic DNA and *Adal*-KD.

A EU incorporation into nascent maternal RNA by a gravid female shown by CuAAC-Alexa 488 reaction in the cytosol (cyan arrow) and nucleolus but not in the nucleus (yellow arrow).

B Cytosolic maternal RNA at 2/4-cell stage embryo.

C CuAAC-Alexa 488 reaction stains the zygotic DNA in a 16-cell stage embryo.

D Negative control displays no staining in a 16-cell stage embryo.

E Embryos injected with sh*Adal1* display no EU incorporation at 64-cell embryos, low at 128-cell, and high incorporation at 256-cell embryos.

Data information: Scale bar in (A): 50 μm. Scale bar in (B–E): 20 μm.
Source data are available online for this figure.

 © 2023 The Authors
