# Supplementary figures and images for: Randomly incorporated genomic N6‐methyldeoxyadenosine delays zygotic transcription initiation in a cnidarian
Source: EMBO J. 2023 Jul 4;42(15):e112934. doi: 10.15252/embj.2022112934 (PMC10390872; doi:10.15252/embj.2022112934)

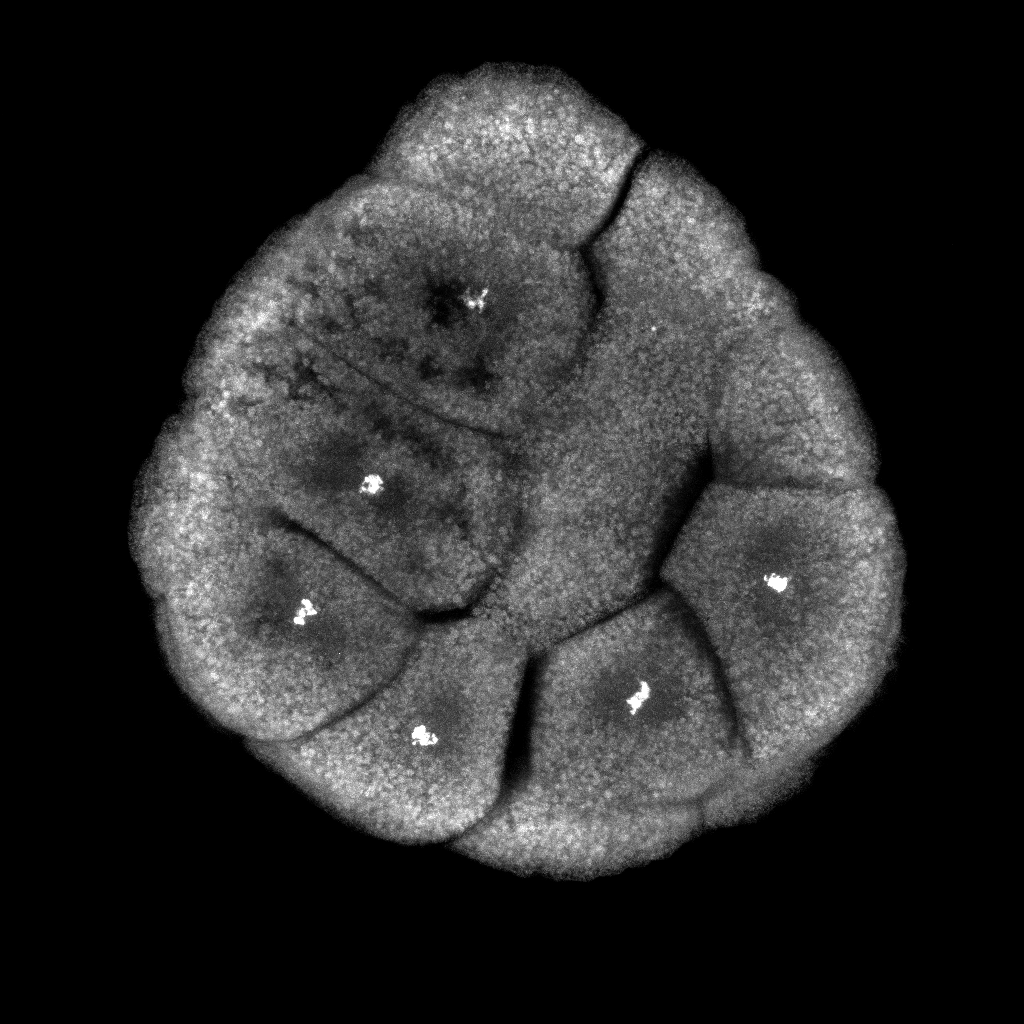

Supplement: Supplementary file 7 — Source Data for Figure 1 [file EMBJ-42-e112934-s004.zip › Fig.1/1D/MAX_C1-EdU-16c.tif]

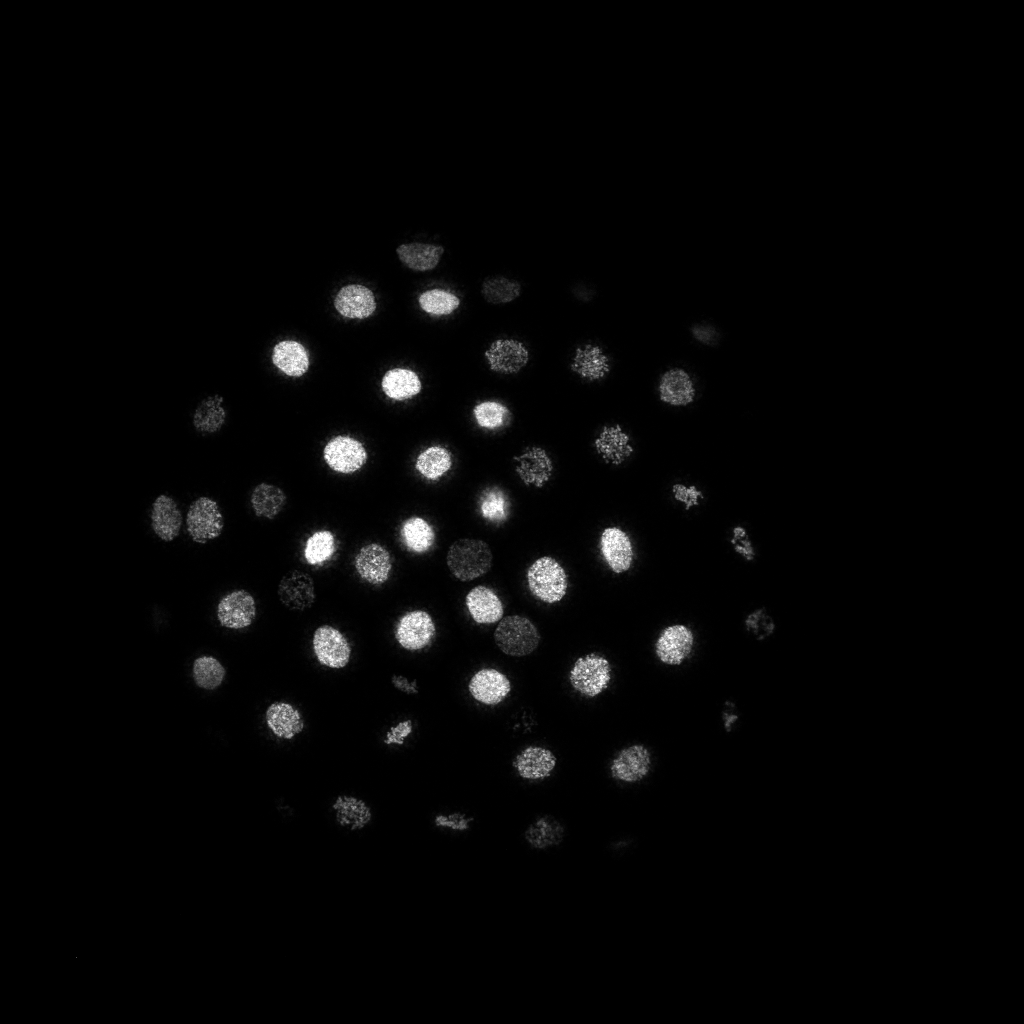

Supplement: Supplementary file 7 — Source Data for Figure 1 [file EMBJ-42-e112934-s004.zip › Fig.1/1D/MAX_C1-EdU-64c.tif]

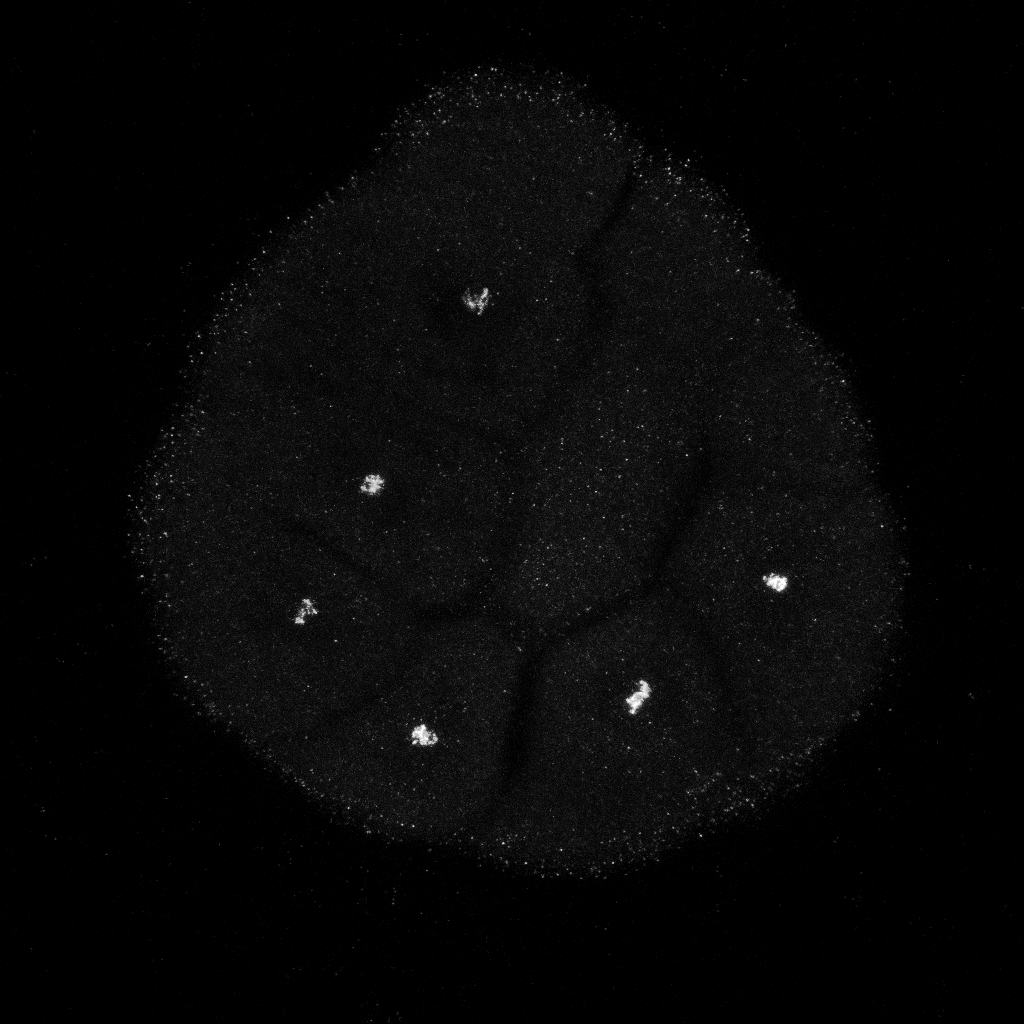

Supplement: Supplementary file 7 — Source Data for Figure 1 [file EMBJ-42-e112934-s004.zip › Fig.1/1D/MAX_C2-6mA-16c.tif]

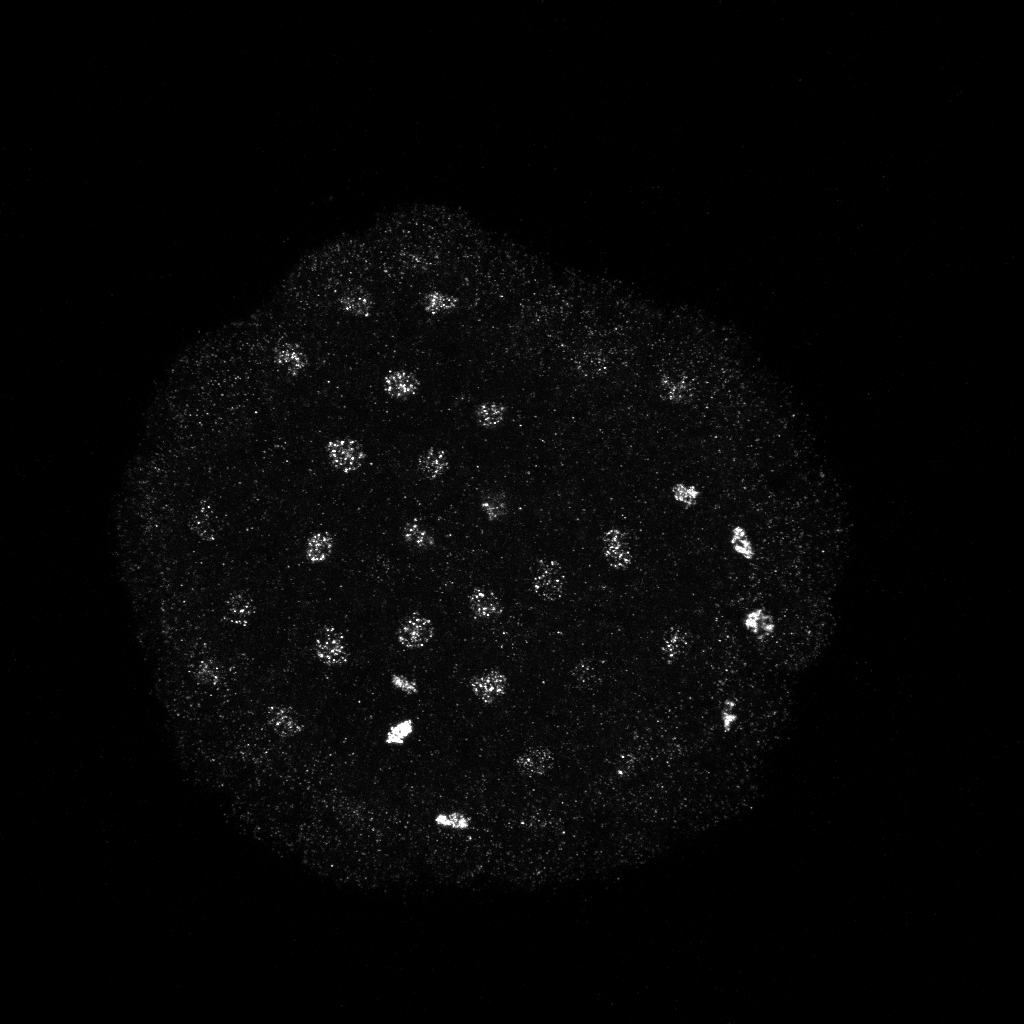

Supplement: Supplementary file 7 — Source Data for Figure 1 [file EMBJ-42-e112934-s004.zip › Fig.1/1D/MAX_C2-6mA-64c.tif]

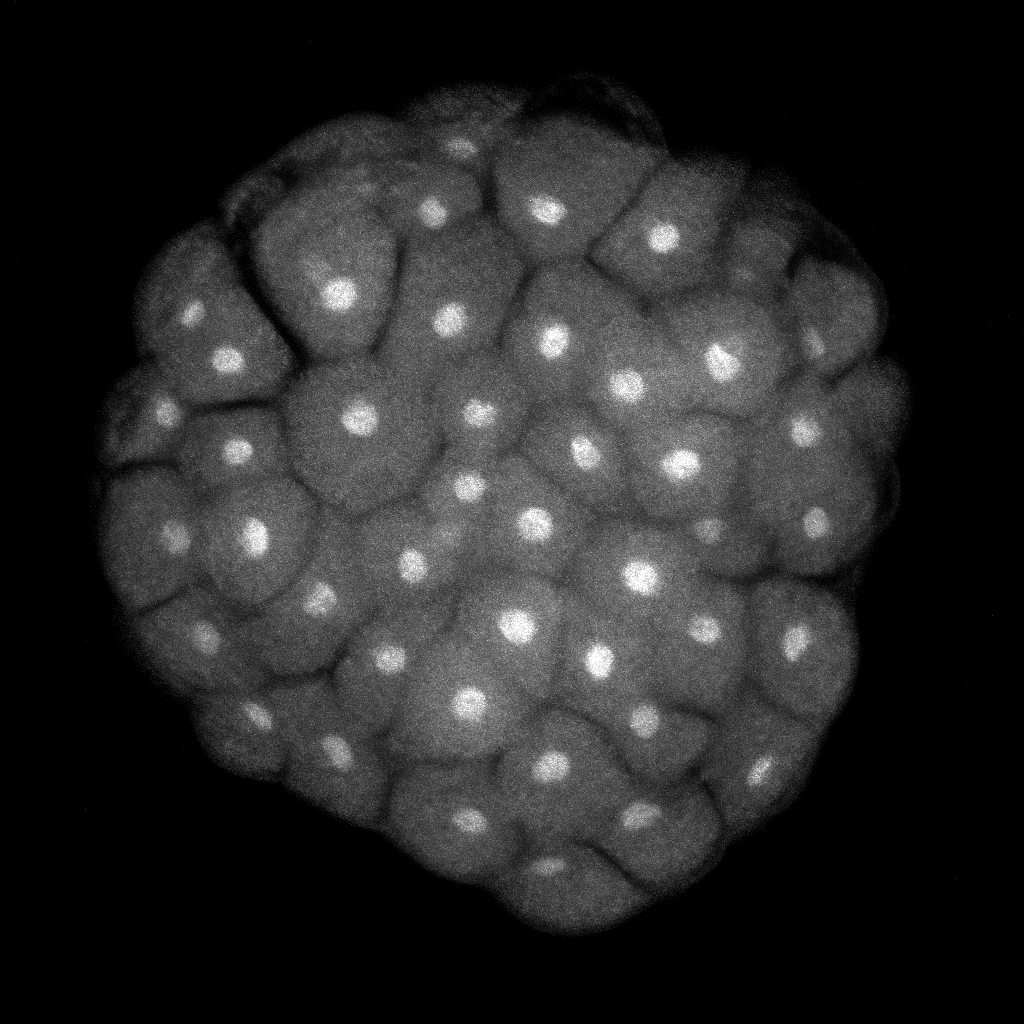

Supplement: Supplementary file 8 — Source Data for Figure 2 [file EMBJ-42-e112934-s001.zip › Fig.2/2A/Rescue/R1/MAX_C1-EdU-Rescue1.tif]

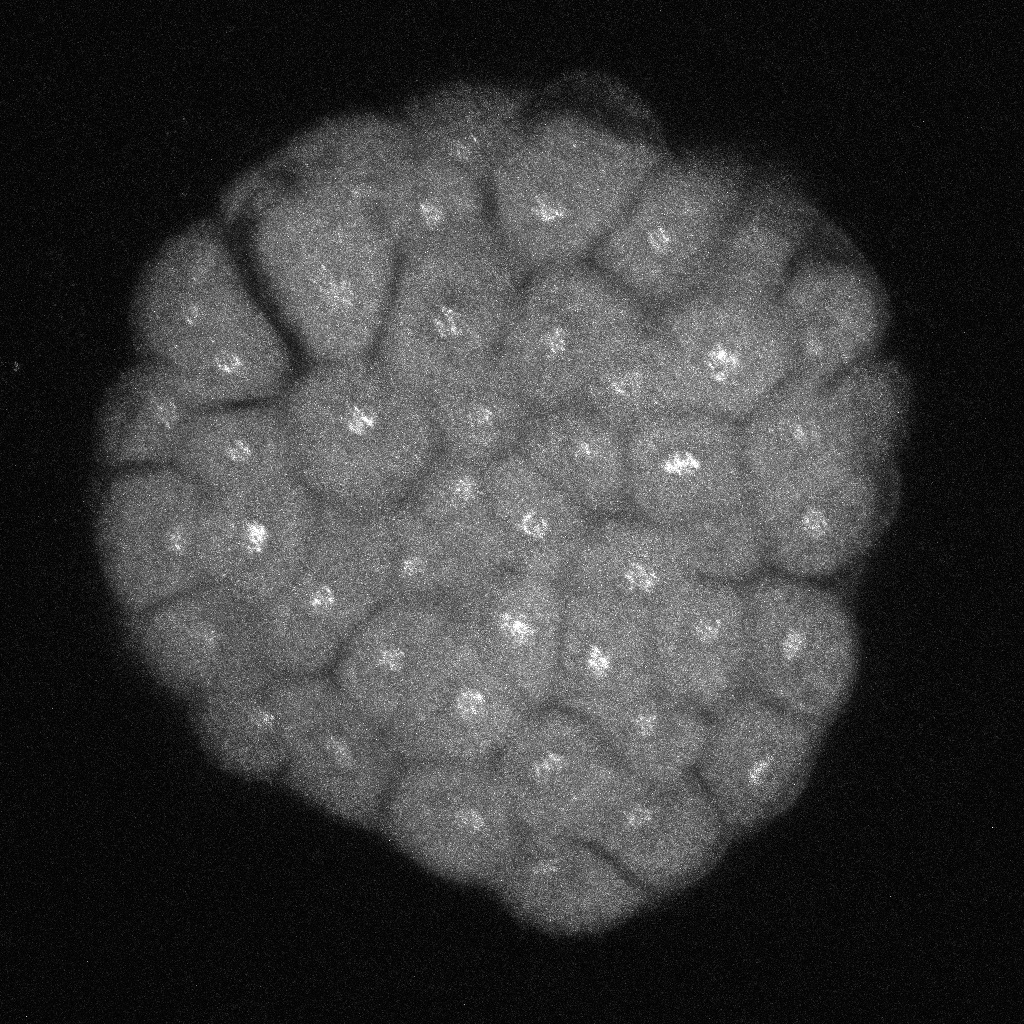

Supplement: Supplementary file 8 — Source Data for Figure 2 [file EMBJ-42-e112934-s001.zip › Fig.2/2A/Rescue/R1/MAX_C2-6mA-Rescue1.tif]

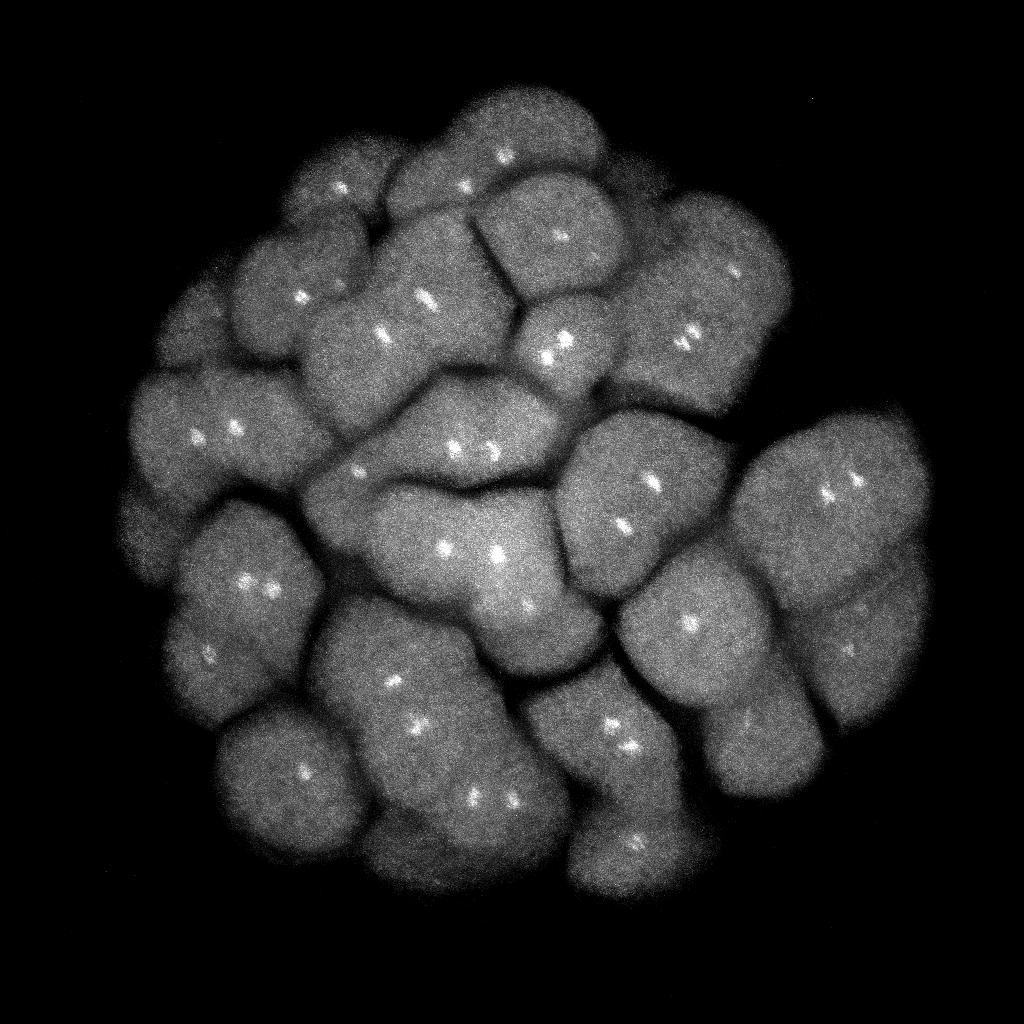

Supplement: Supplementary file 8 — Source Data for Figure 2 [file EMBJ-42-e112934-s001.zip › Fig.2/2A/Rescue/R2/MAX_C1-EdU-Rescue2.tif]

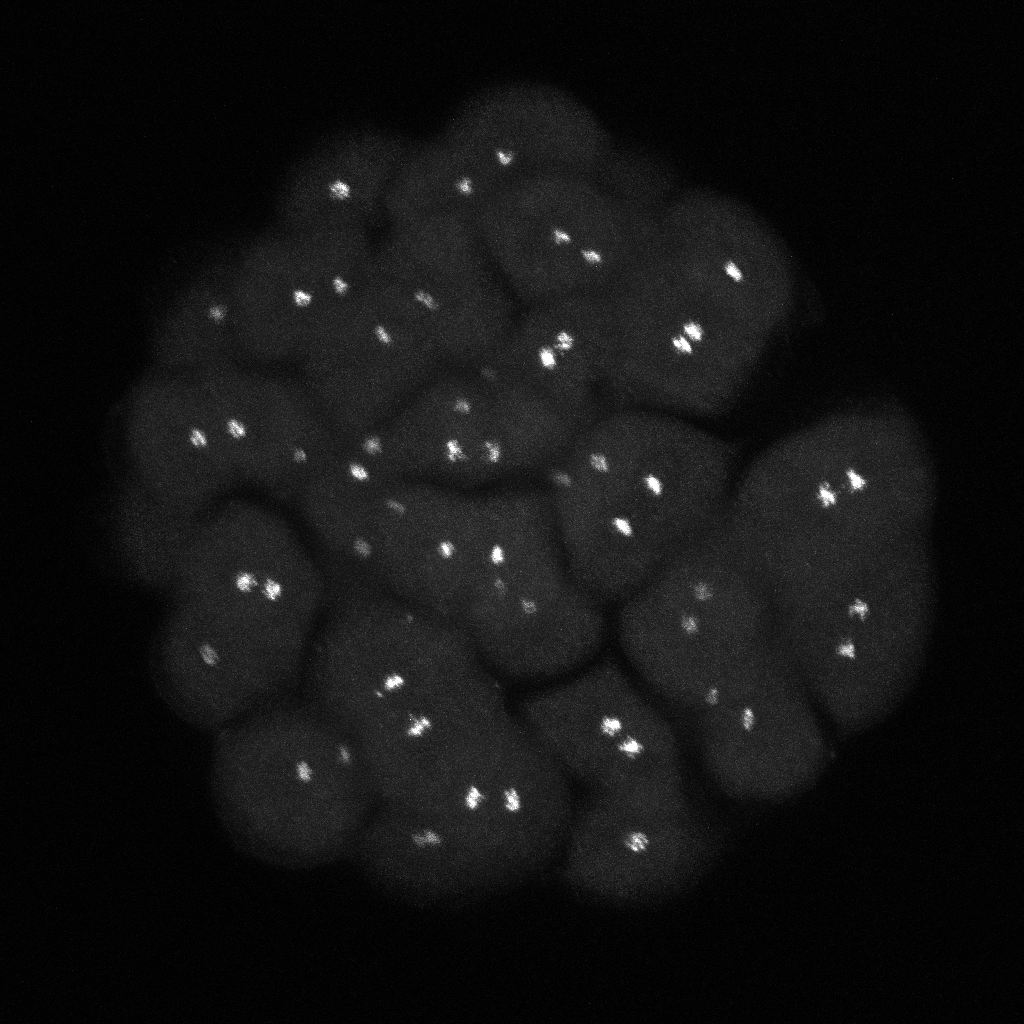

Supplement: Supplementary file 8 — Source Data for Figure 2 [file EMBJ-42-e112934-s001.zip › Fig.2/2A/Rescue/R2/MAX_C2-6mA-Rescue2.tif]

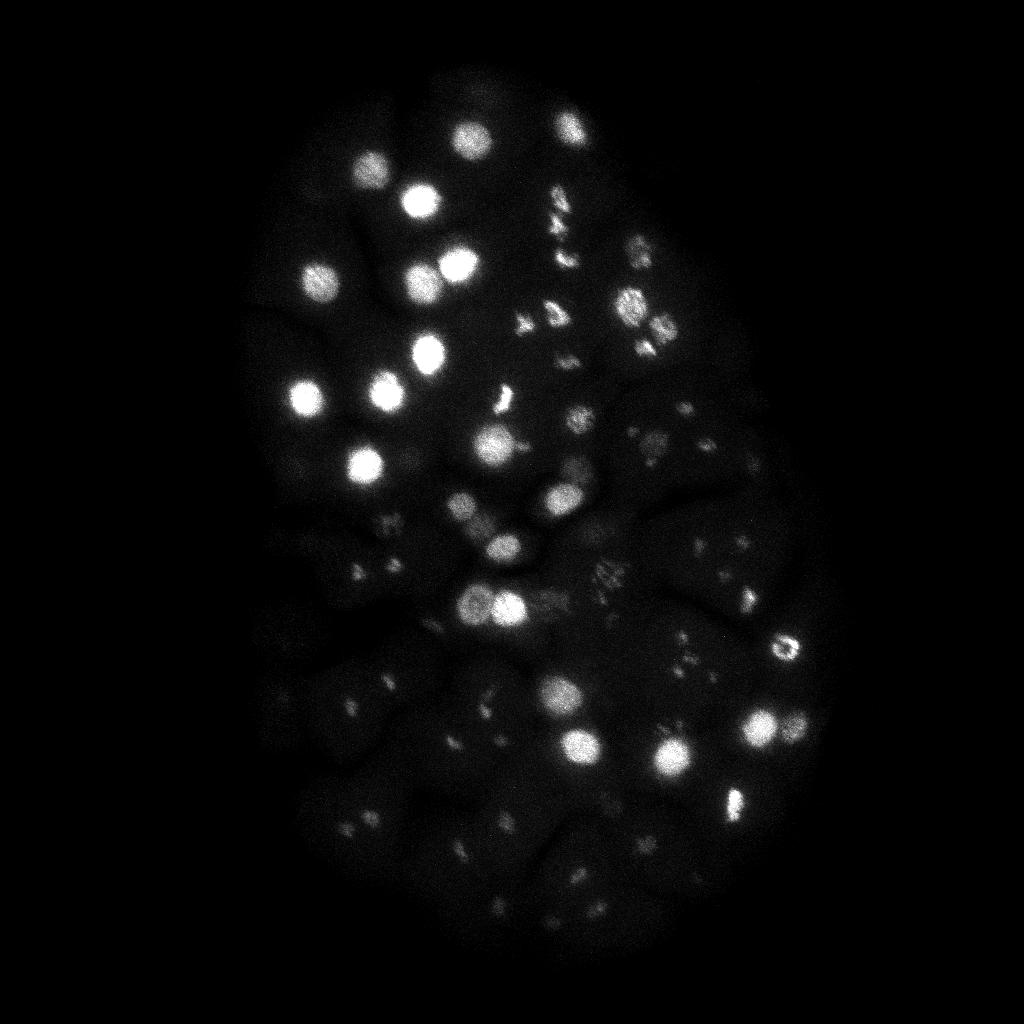

Supplement: Supplementary file 8 — Source Data for Figure 2 [file EMBJ-42-e112934-s001.zip › Fig.2/2A/Rescue/R3/MAX_C1-EdU-Rescue3.tif]

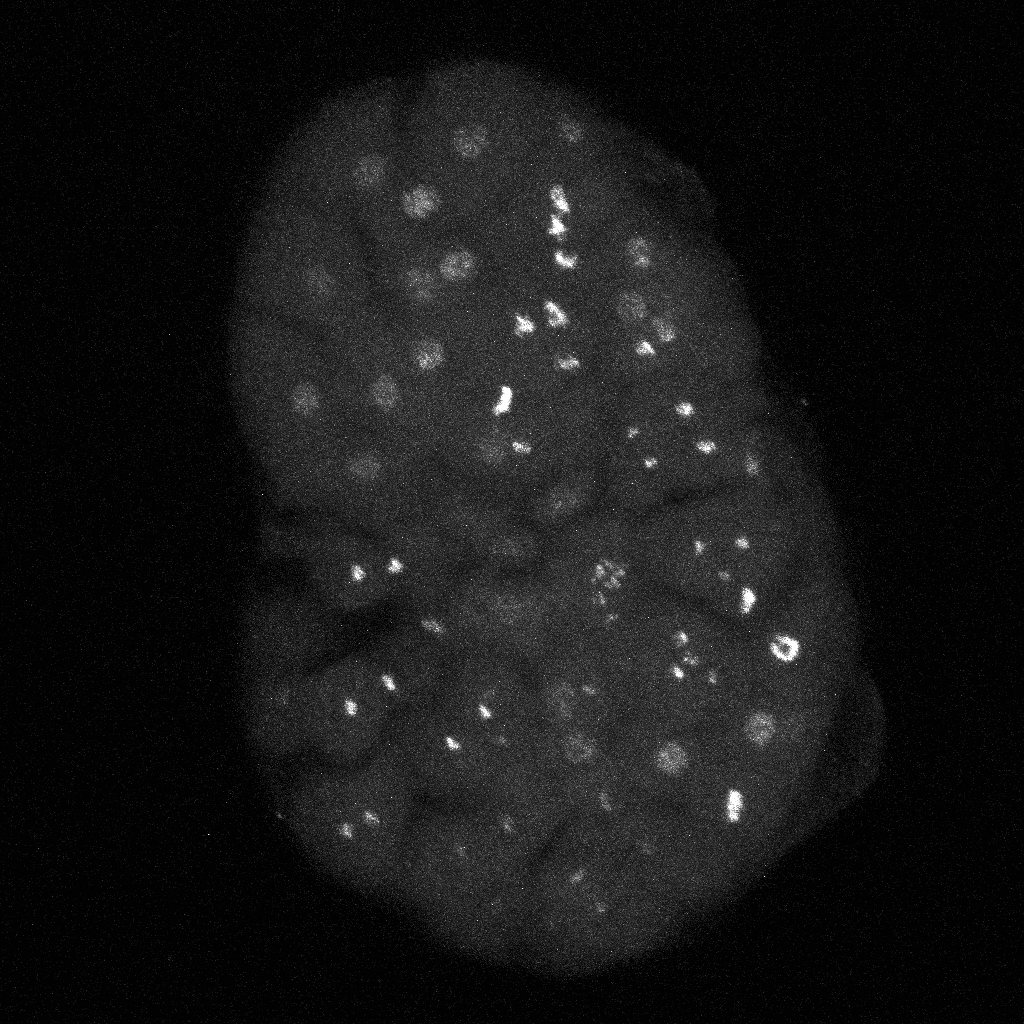

Supplement: Supplementary file 8 — Source Data for Figure 2 [file EMBJ-42-e112934-s001.zip › Fig.2/2A/Rescue/R3/MAX_C2-6mA-Rescue3.tif]

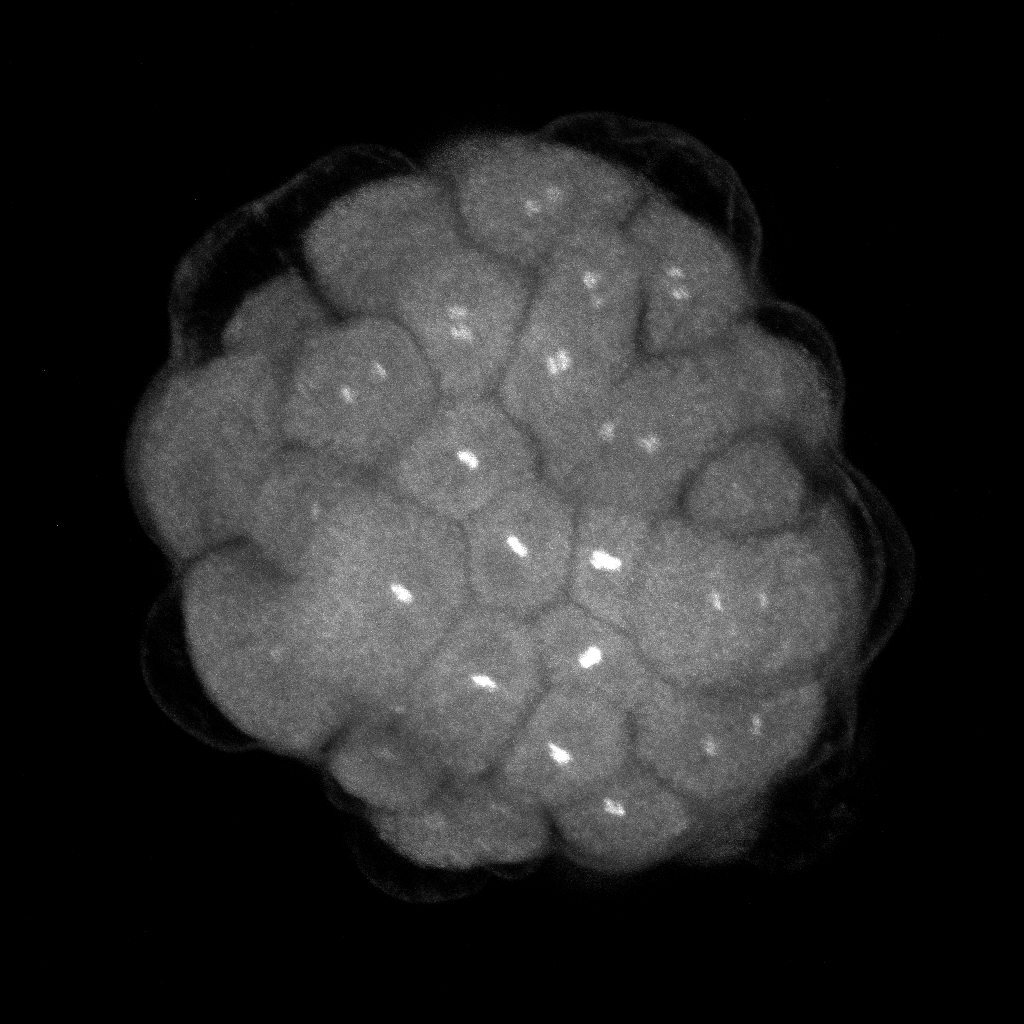

Supplement: Supplementary file 8 — Source Data for Figure 2 [file EMBJ-42-e112934-s001.zip › Fig.2/2A/Rescue/R4/MAX_C1-EdU-Rescue4.tif]

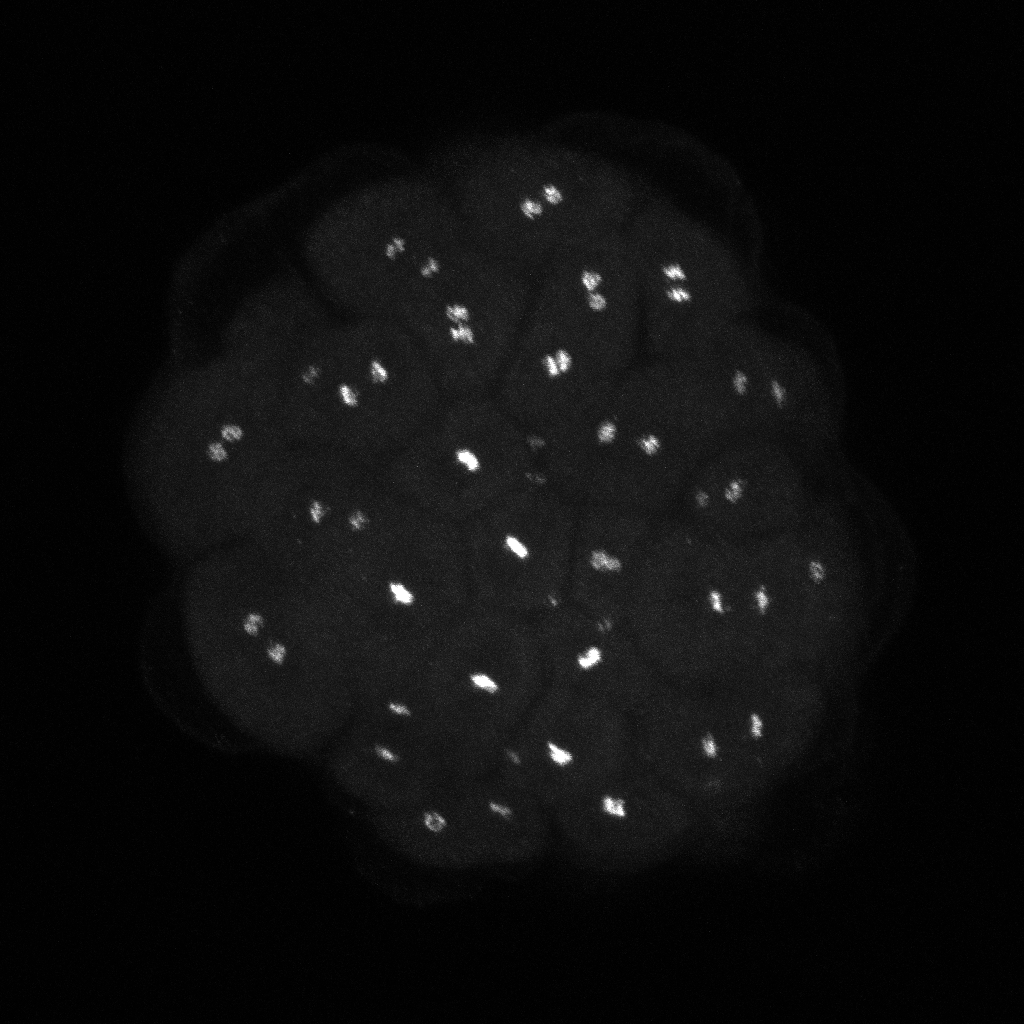

Supplement: Supplementary file 8 — Source Data for Figure 2 [file EMBJ-42-e112934-s001.zip › Fig.2/2A/Rescue/R4/MAX_C2-6mA-Rescue4.tif]

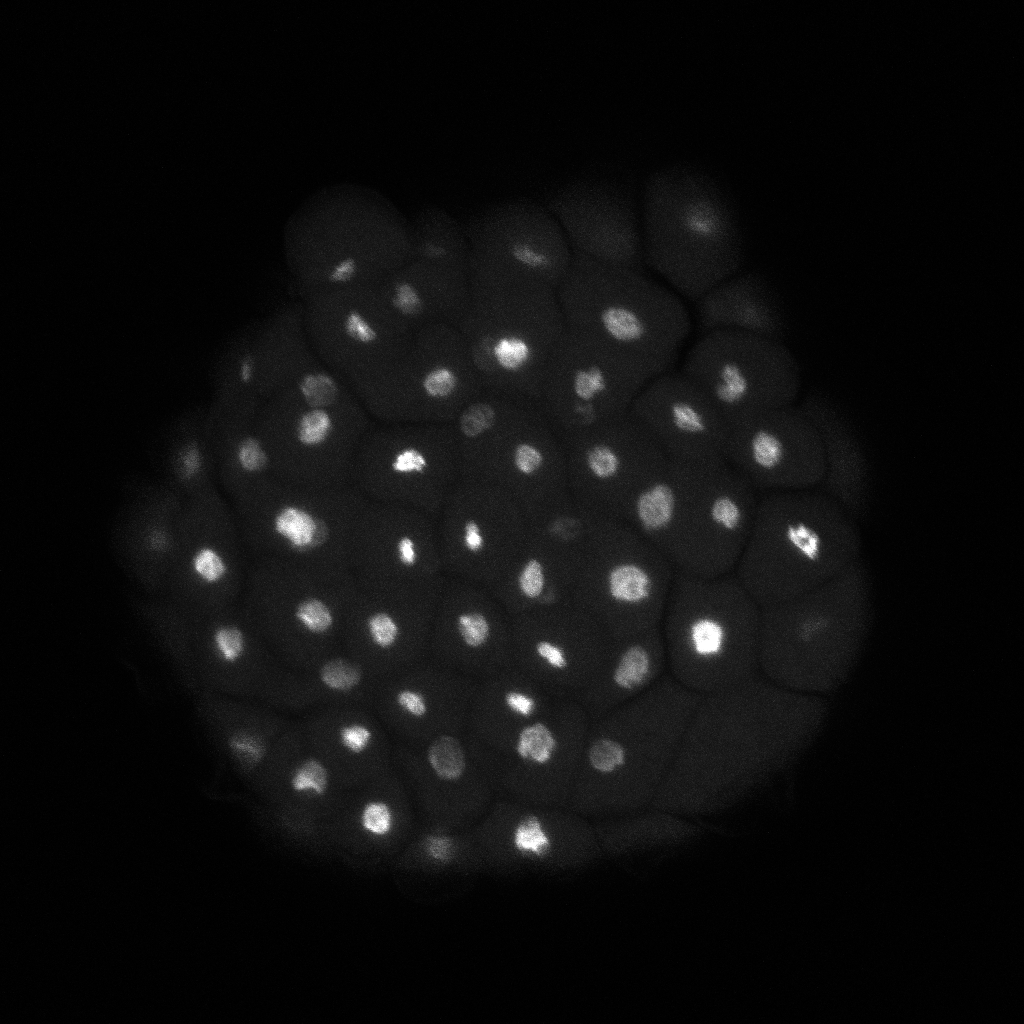

Supplement: Supplementary file 8 — Source Data for Figure 2 [file EMBJ-42-e112934-s001.zip › Fig.2/2A/shAlkbh1/R1/MAX_C1-EdU-001.tif]

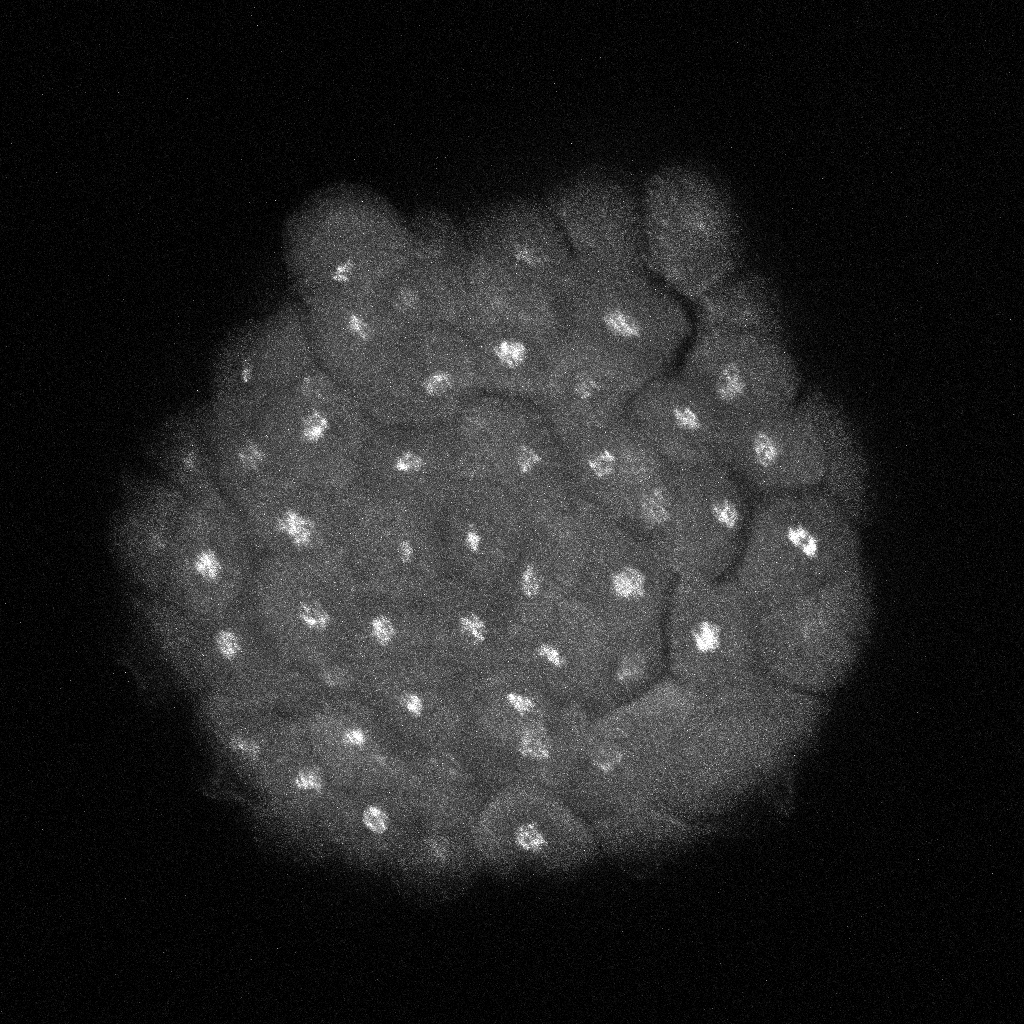

Supplement: Supplementary file 8 — Source Data for Figure 2 [file EMBJ-42-e112934-s001.zip › Fig.2/2A/shAlkbh1/R1/MAX_C2-6mA-001.tif]

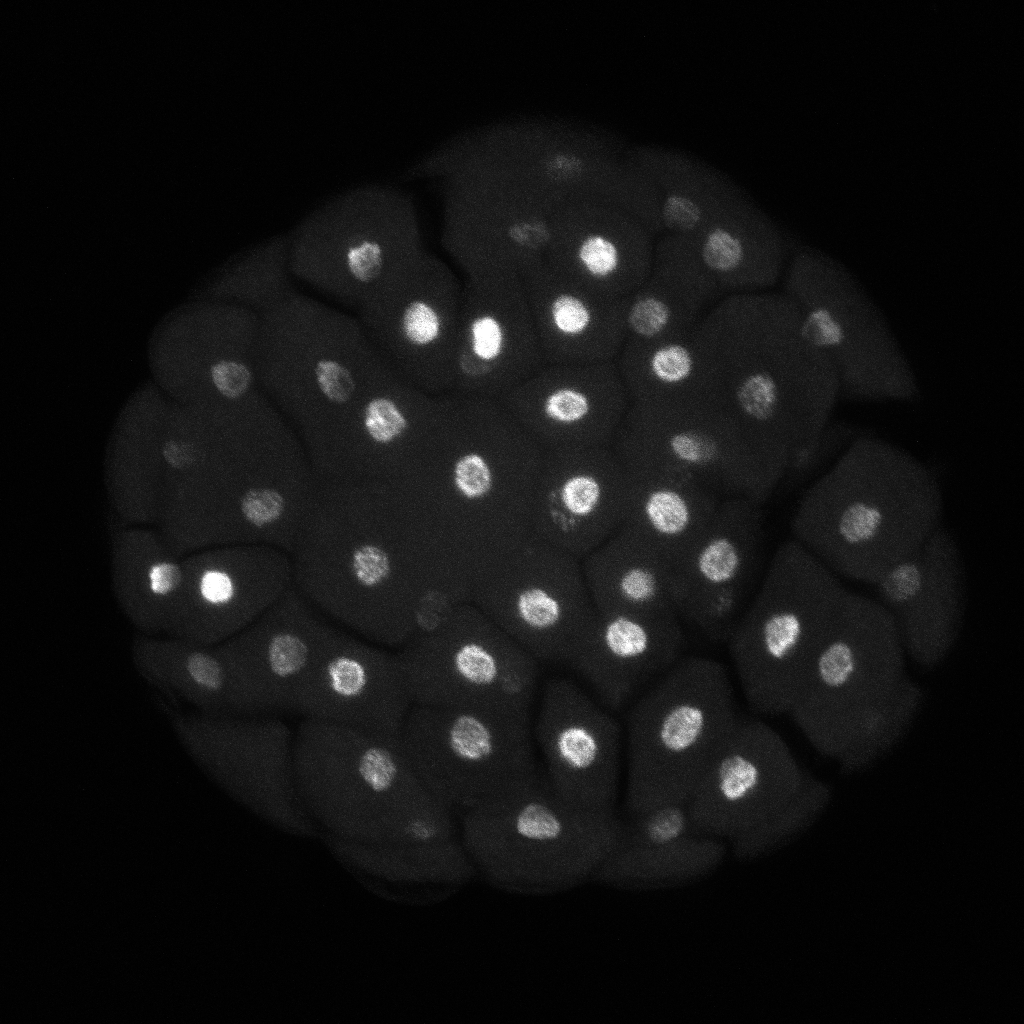

Supplement: Supplementary file 8 — Source Data for Figure 2 [file EMBJ-42-e112934-s001.zip › Fig.2/2A/shAlkbh1/R2/MAX_C1-EdU-002.tif]

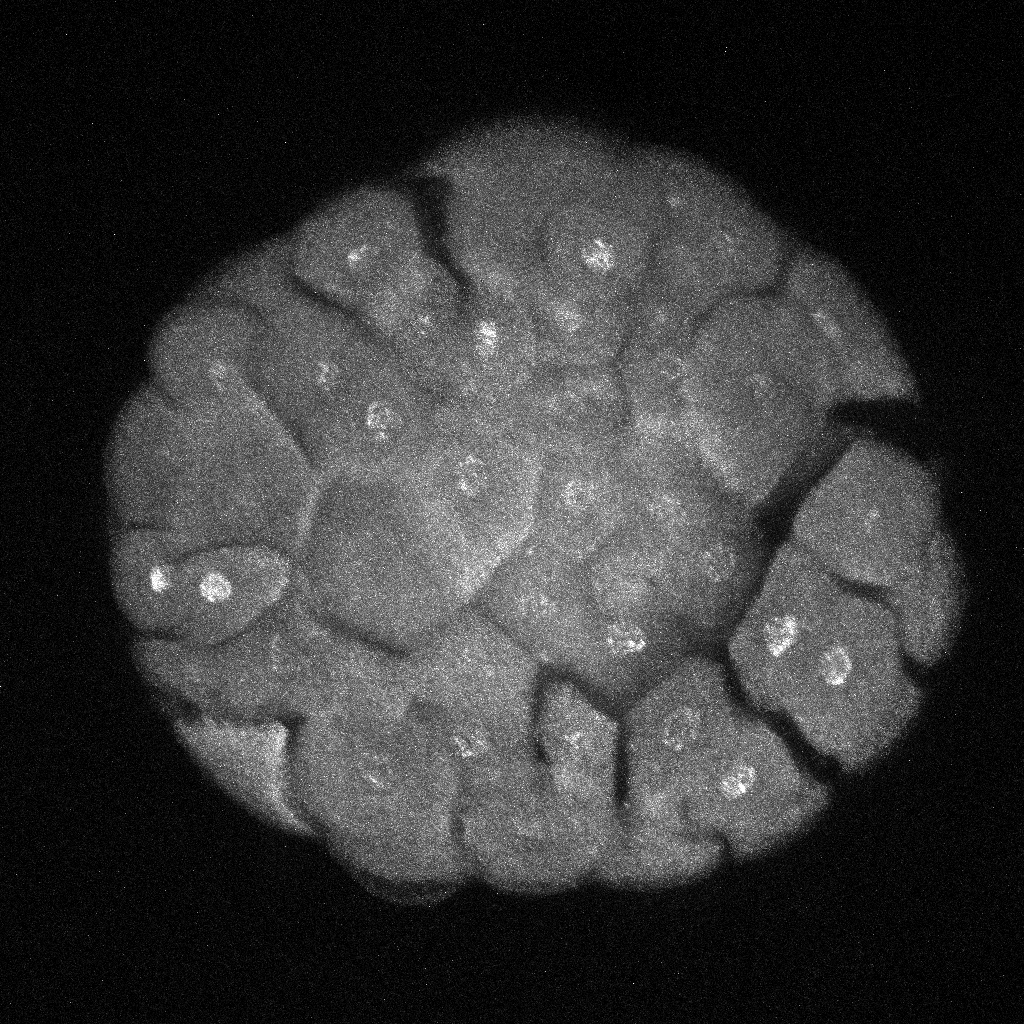

Supplement: Supplementary file 8 — Source Data for Figure 2 [file EMBJ-42-e112934-s001.zip › Fig.2/2A/shAlkbh1/R2/MAX_C2-6mA-002.tif]

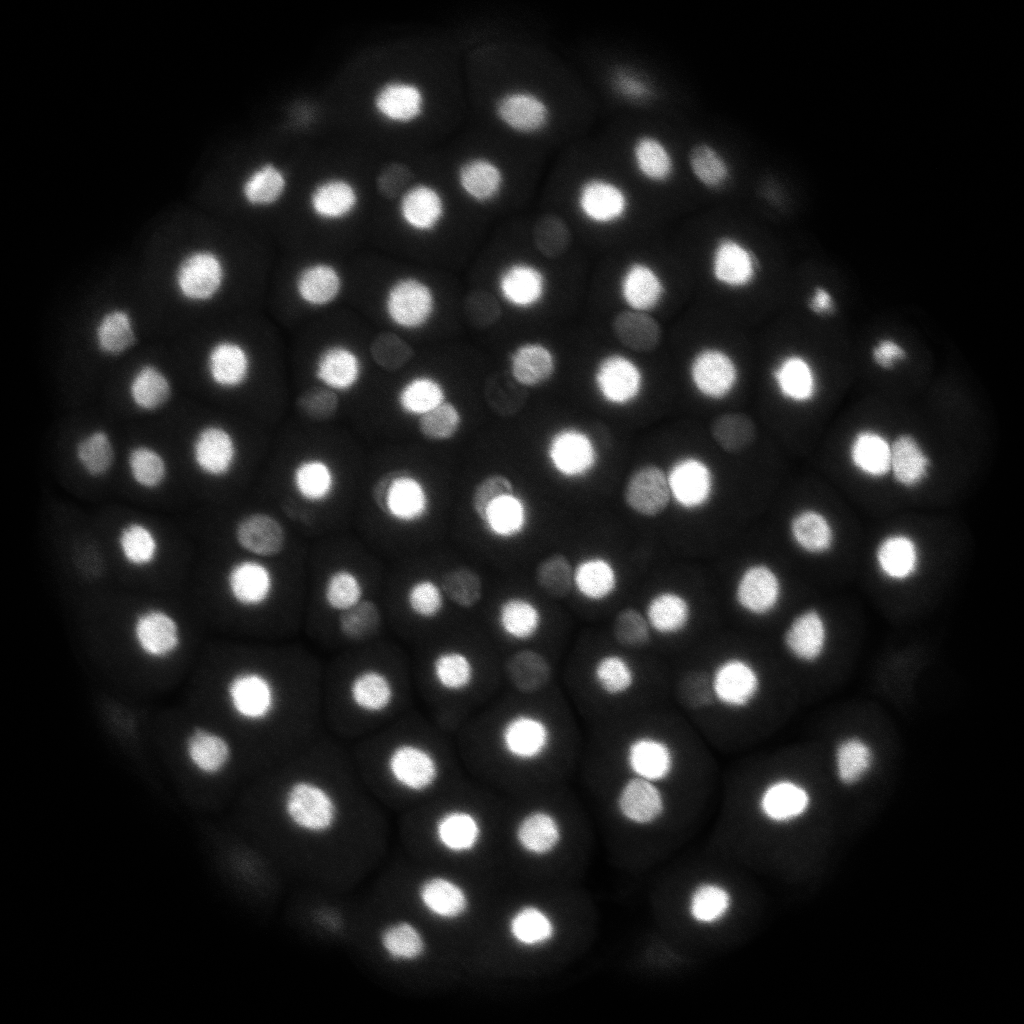

Supplement: Supplementary file 8 — Source Data for Figure 2 [file EMBJ-42-e112934-s001.zip › Fig.2/2A/shAlkbh1/R3/MAX_C1-EdU-003.tif]

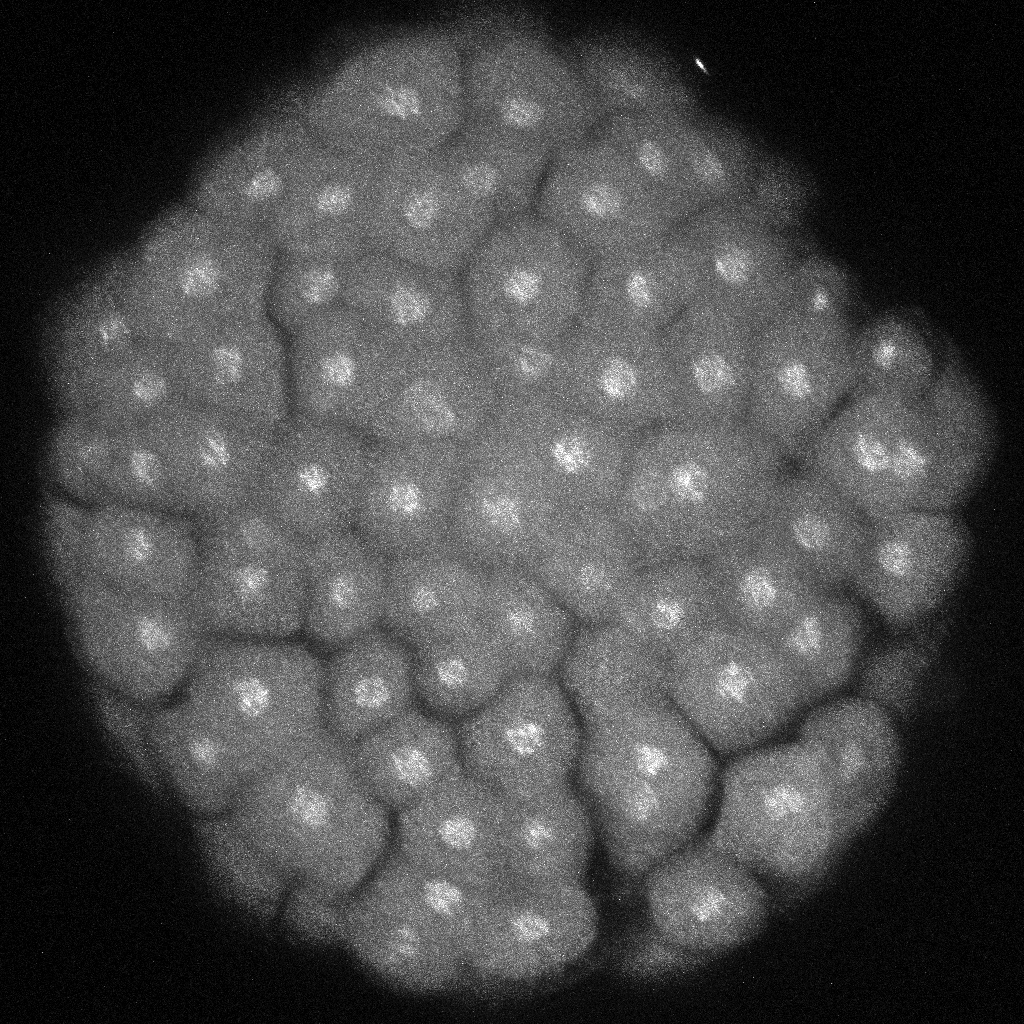

Supplement: Supplementary file 8 — Source Data for Figure 2 [file EMBJ-42-e112934-s001.zip › Fig.2/2A/shAlkbh1/R3/MAX_C2-6mA-003.tif]

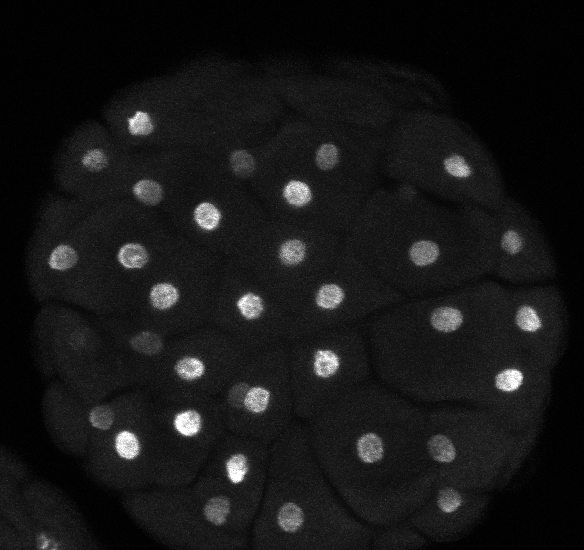

Supplement: Supplementary file 8 — Source Data for Figure 2 [file EMBJ-42-e112934-s001.zip › Fig.2/2A/shGfp/R1/MAX_C1-EdU-001.tif]

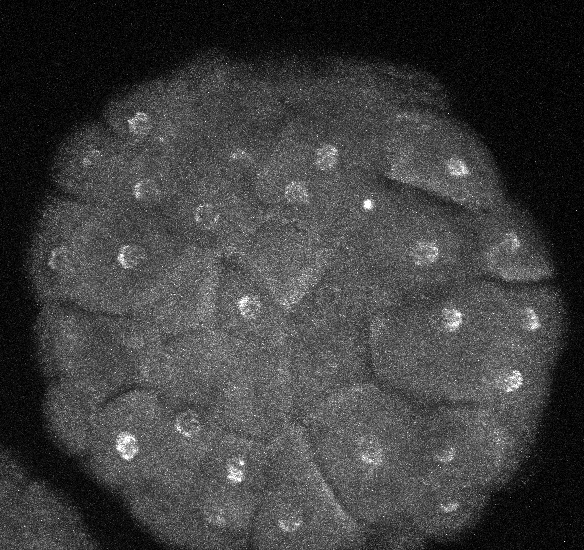

Supplement: Supplementary file 8 — Source Data for Figure 2 [file EMBJ-42-e112934-s001.zip › Fig.2/2A/shGfp/R1/MAX_C2-6mA-001.tif]

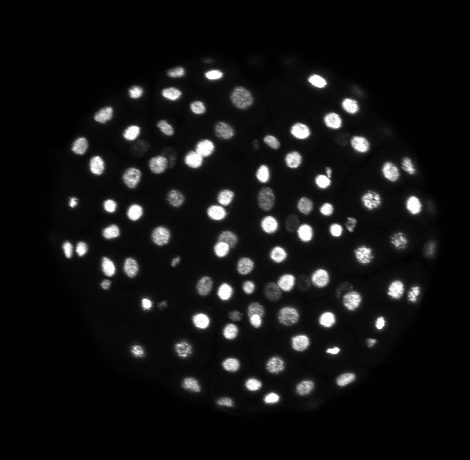

Supplement: Supplementary file 8 — Source Data for Figure 2 [file EMBJ-42-e112934-s001.zip › Fig.2/2A/shGfp/R2/MAX_C1-EdU-002.tif]

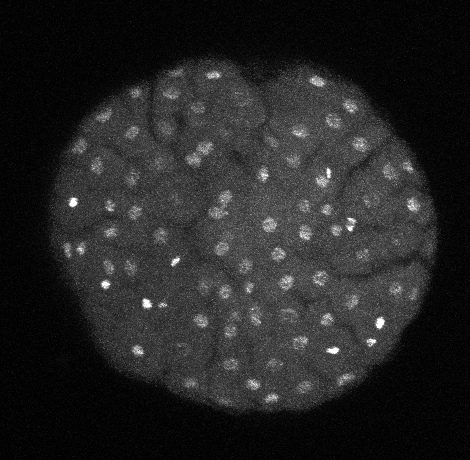

Supplement: Supplementary file 8 — Source Data for Figure 2 [file EMBJ-42-e112934-s001.zip › Fig.2/2A/shGfp/R2/MAX_C2-6mA-002.tif]

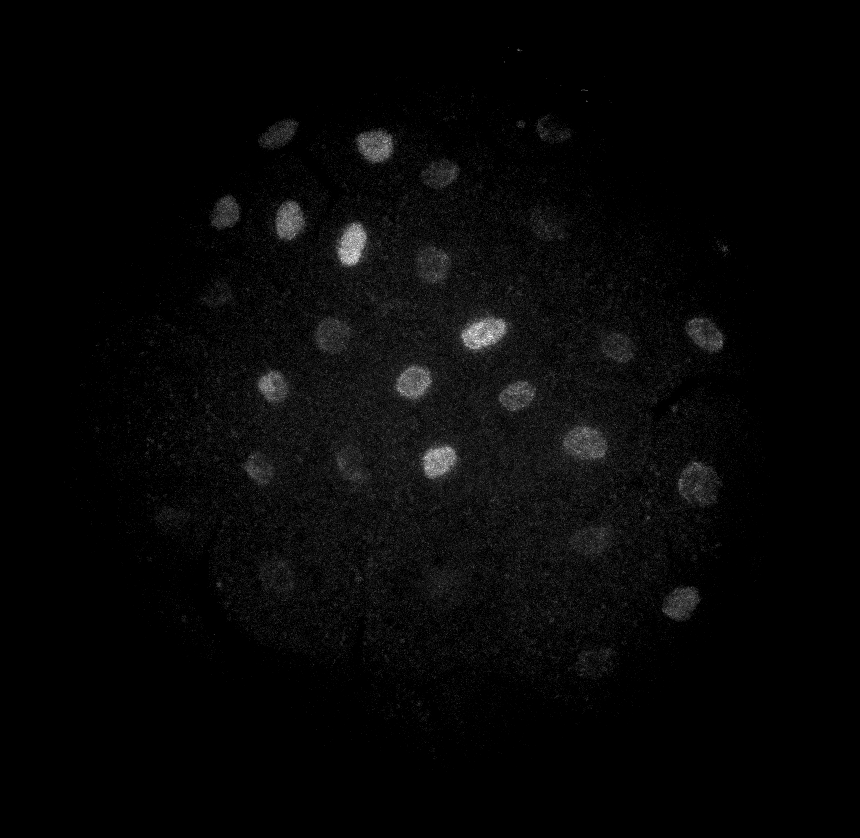

Supplement: Supplementary file 8 — Source Data for Figure 2 [file EMBJ-42-e112934-s001.zip › Fig.2/2A/shGfp/R3/MAX_C1_EdU_003.tif]

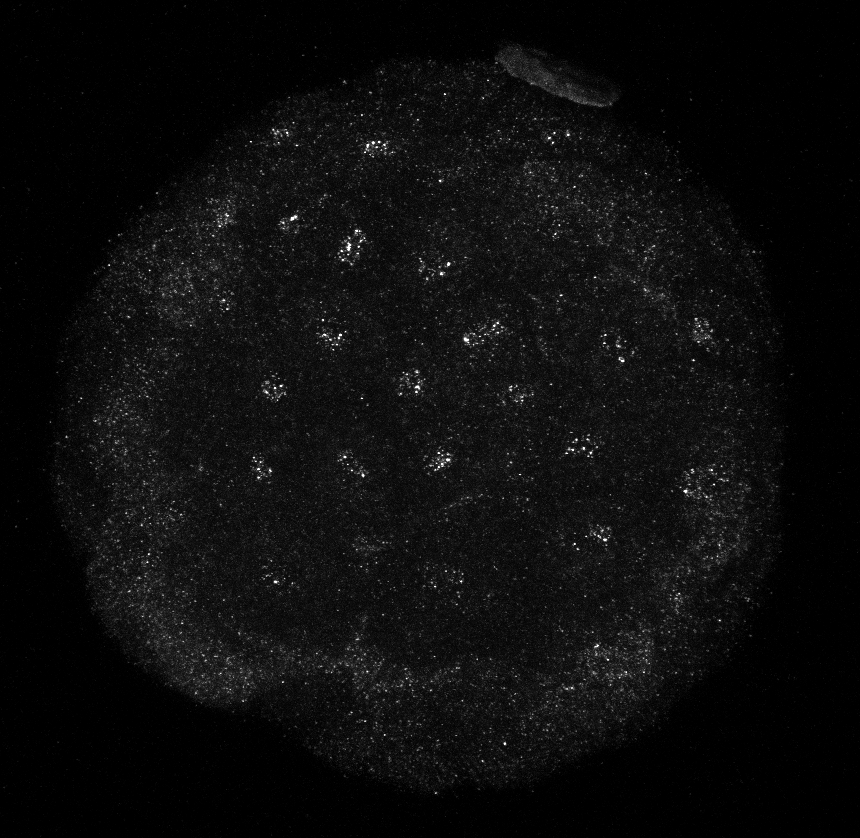

Supplement: Supplementary file 8 — Source Data for Figure 2 [file EMBJ-42-e112934-s001.zip › Fig.2/2A/shGfp/R3/MAX_C2_6mA-003.tif]

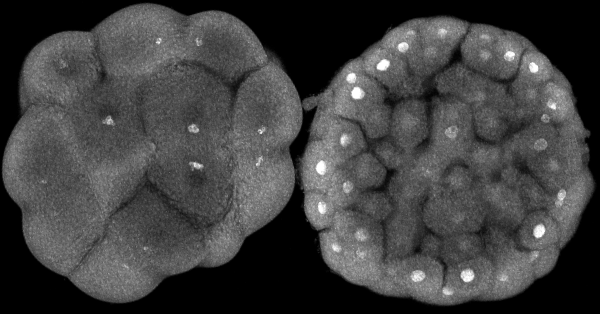

Supplement: Supplementary file 9 — Source Data for Figure 3 [file EMBJ-42-e112934-s008.zip › Fig.3/3B/MAX_C1-RawEU.tif]

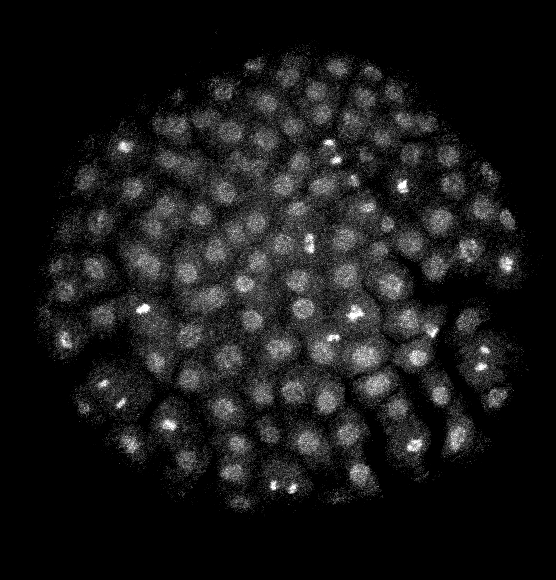

Supplement: Supplementary file 9 — Source Data for Figure 3 [file EMBJ-42-e112934-s008.zip › Fig.3/3B/MAX_C1-RNase.tif]

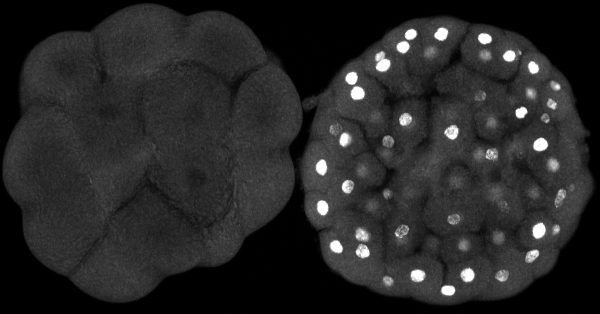

Supplement: Supplementary file 9 — Source Data for Figure 3 [file EMBJ-42-e112934-s008.zip › Fig.3/3B/MAX_C2-RawEU.tif]

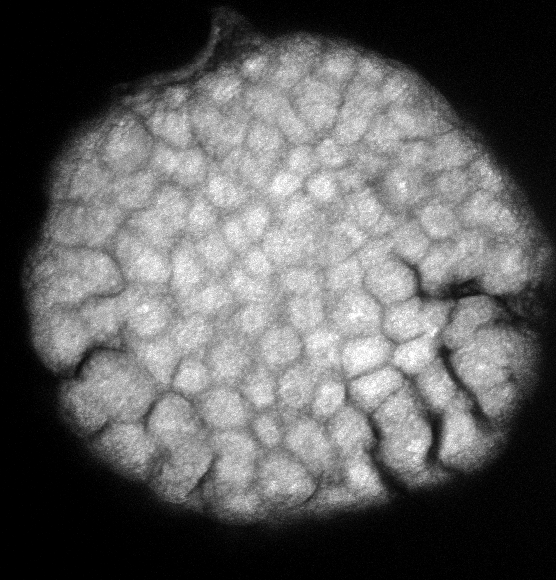

Supplement: Supplementary file 9 — Source Data for Figure 3 [file EMBJ-42-e112934-s008.zip › Fig.3/3B/MAX_C2-RNase.tif]

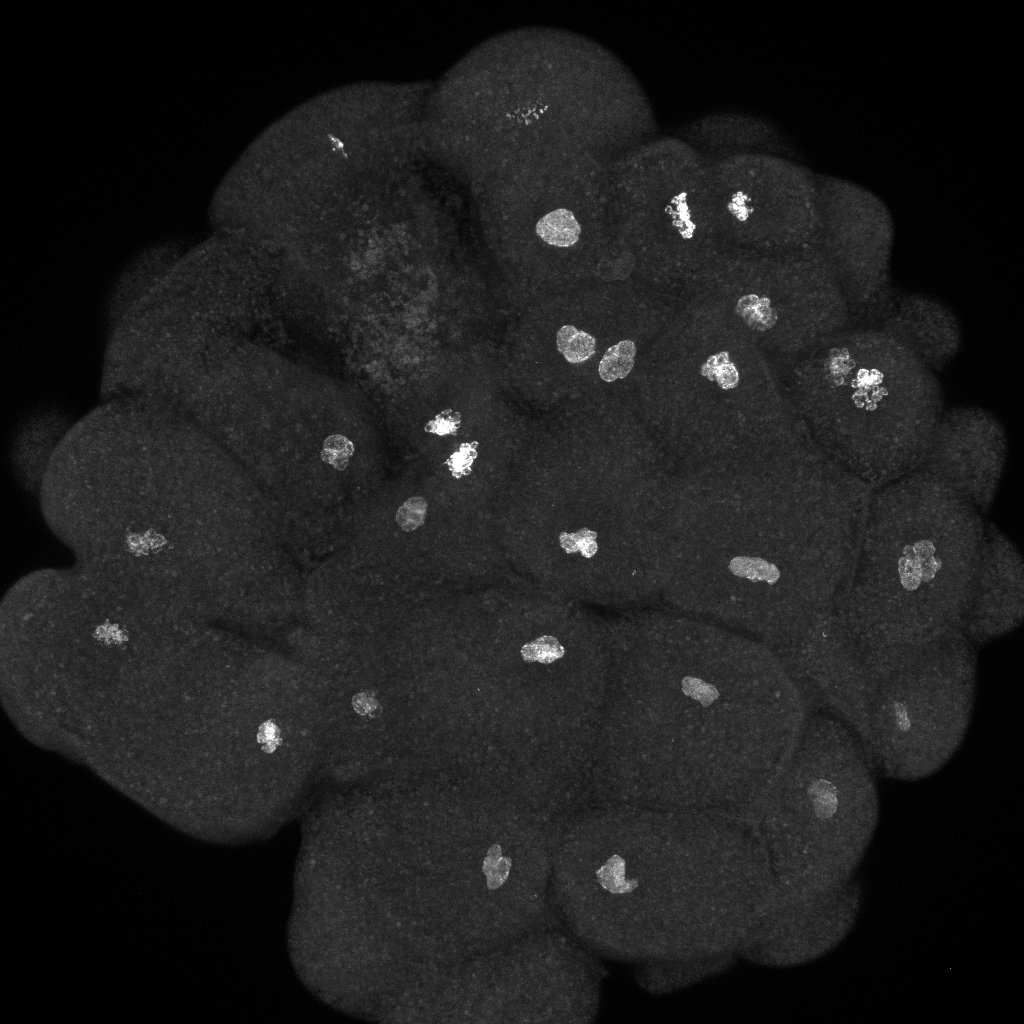

Supplement: Supplementary file 10 — Source Data for Figure 4 [file EMBJ-42-e112934-s007.zip › Fig.4/4A/Mutant rescue/R1/MAX_C1-DAPI-MutRes01.tif]

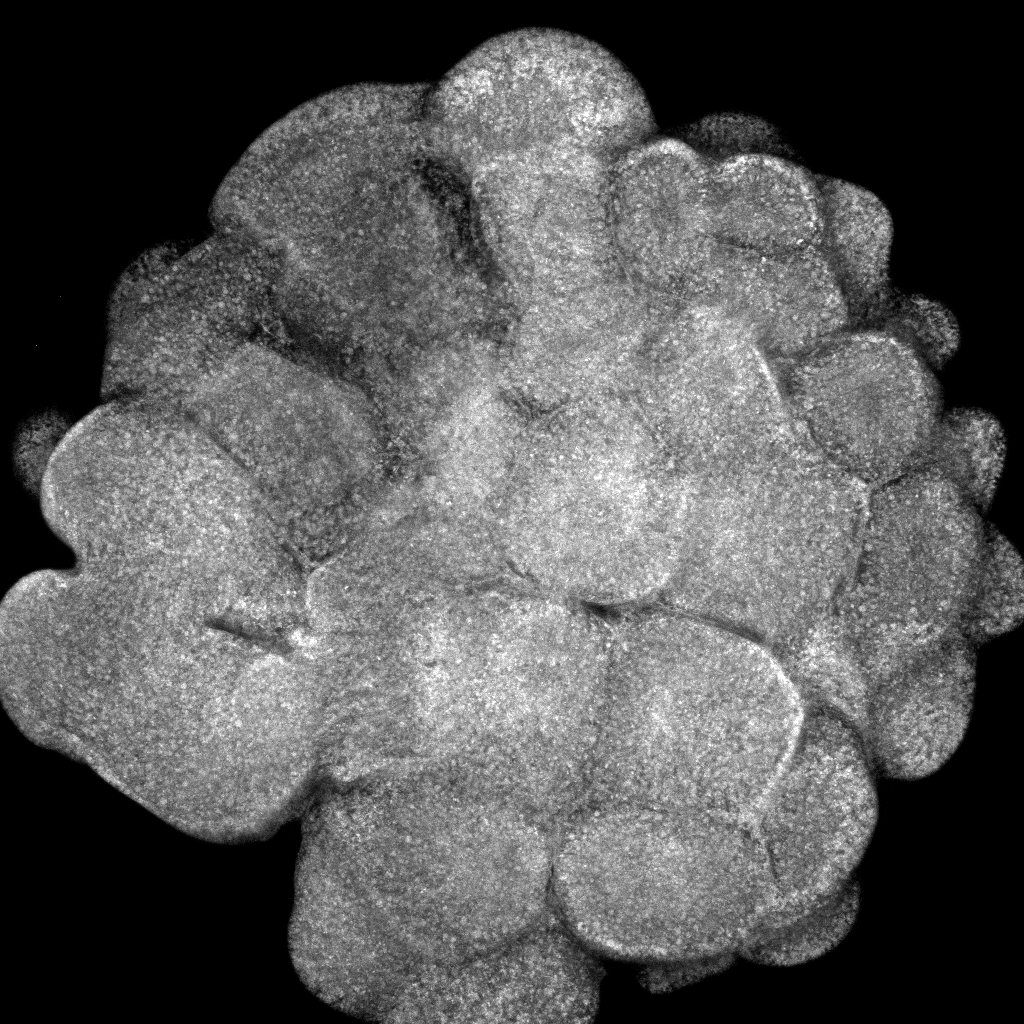

Supplement: Supplementary file 10 — Source Data for Figure 4 [file EMBJ-42-e112934-s007.zip › Fig.4/4A/Mutant rescue/R1/MAX_C2-EU-MutRes01.tif]

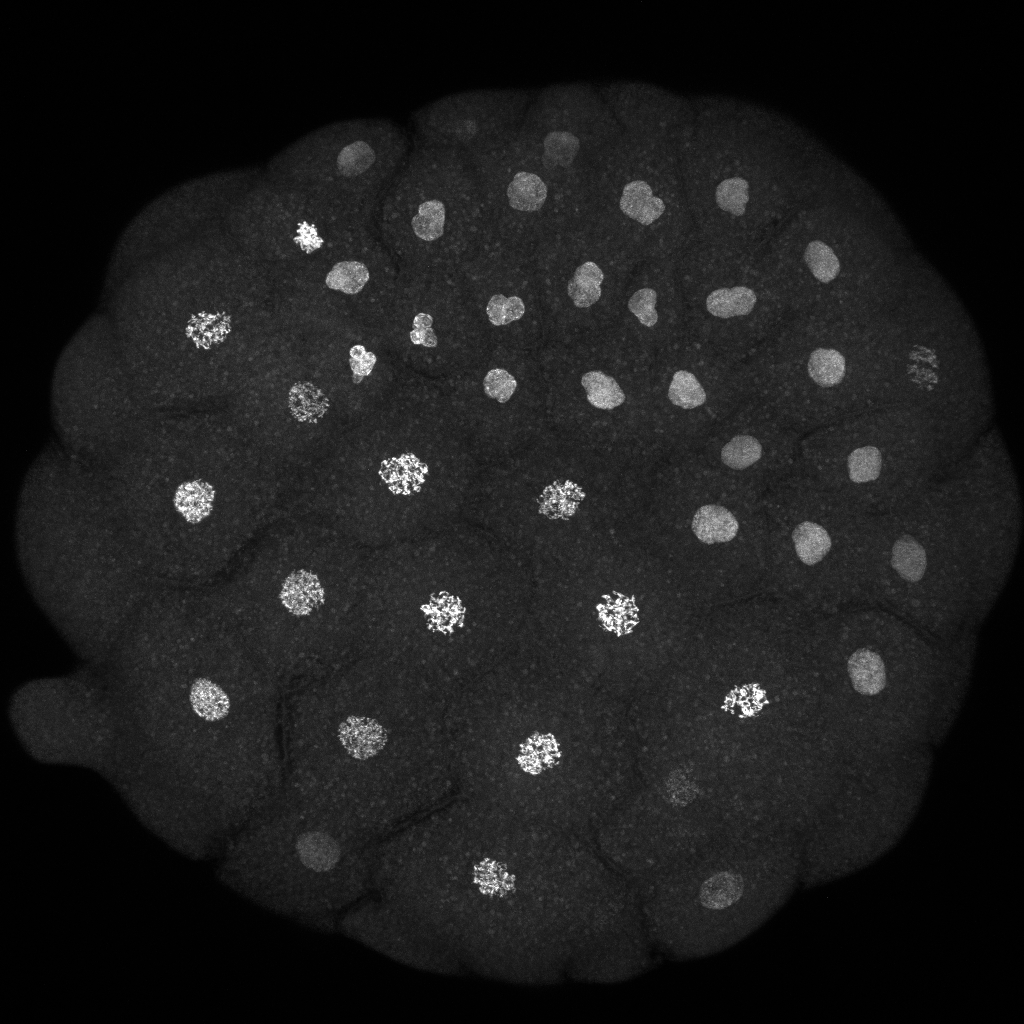

Supplement: Supplementary file 10 — Source Data for Figure 4 [file EMBJ-42-e112934-s007.zip › Fig.4/4A/Mutant rescue/R2/MAX_C1-DAPI_MutRes02.tif]

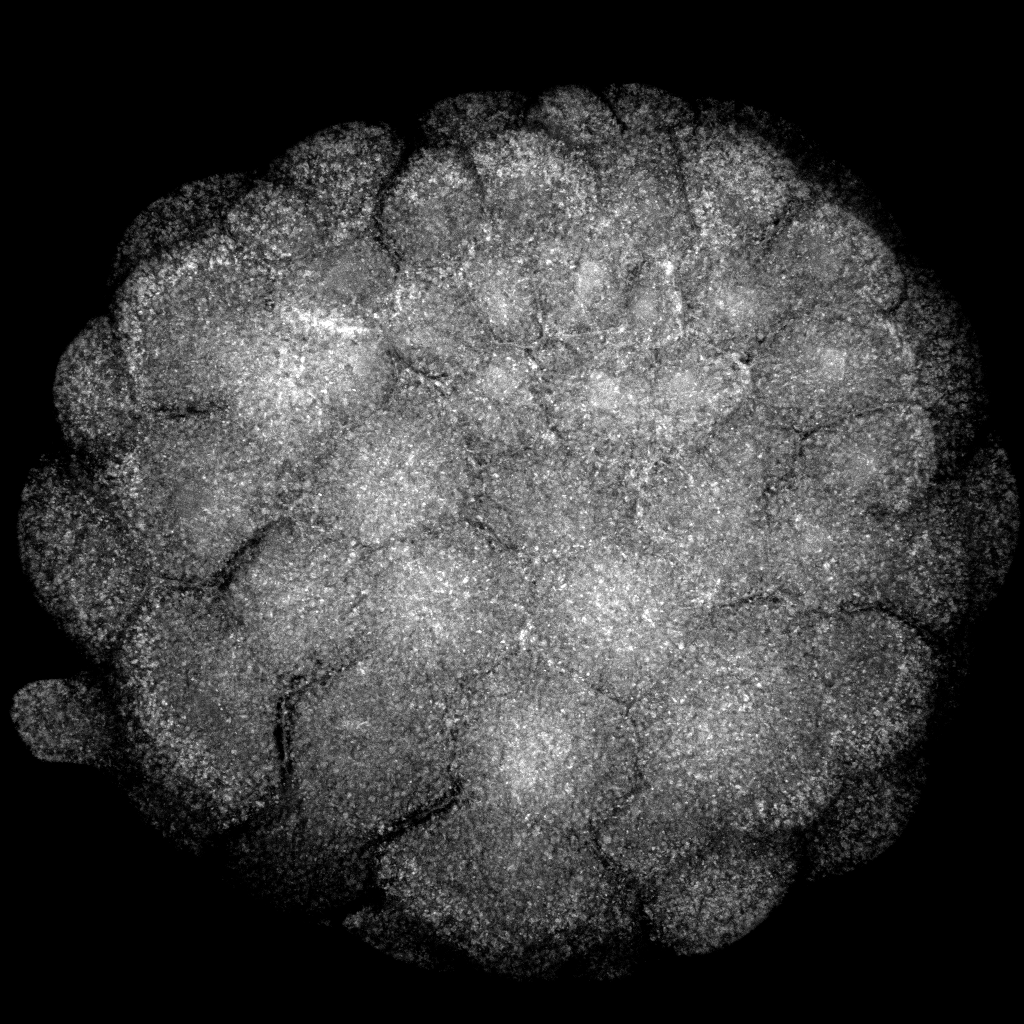

Supplement: Supplementary file 10 — Source Data for Figure 4 [file EMBJ-42-e112934-s007.zip › Fig.4/4A/Mutant rescue/R2/MAX_C2-EU_MutRes02.tif]

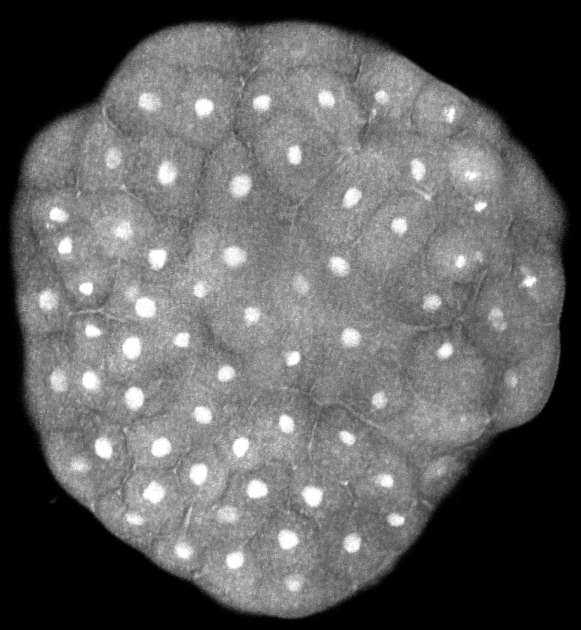

Supplement: Supplementary file 10 — Source Data for Figure 4 [file EMBJ-42-e112934-s007.zip › Fig.4/4A/rescue/R1/MAX_C1-DAPI-Rescue1.tif]

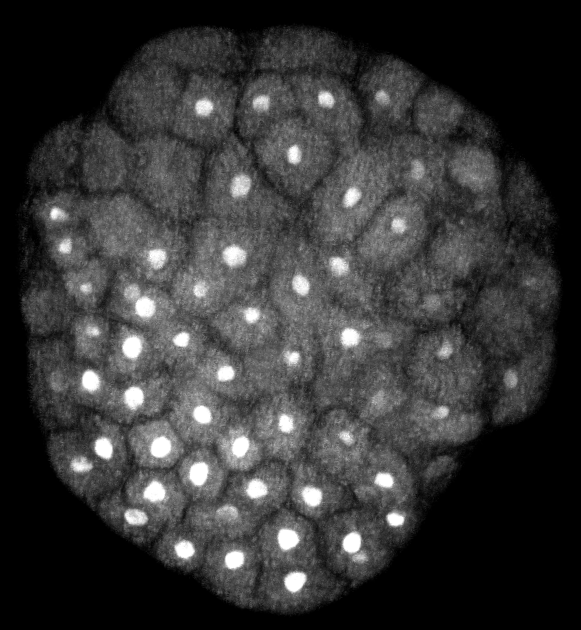

Supplement: Supplementary file 10 — Source Data for Figure 4 [file EMBJ-42-e112934-s007.zip › Fig.4/4A/rescue/R1/MAX_C2-EU-Rescue1.tif]

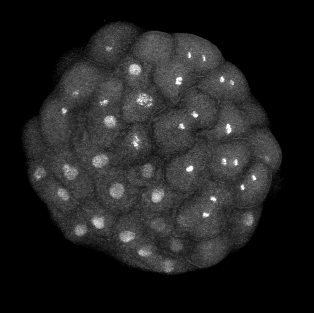

Supplement: Supplementary file 10 — Source Data for Figure 4 [file EMBJ-42-e112934-s007.zip › Fig.4/4A/rescue/R2/MAX_C1-DAPI_R2.tif]

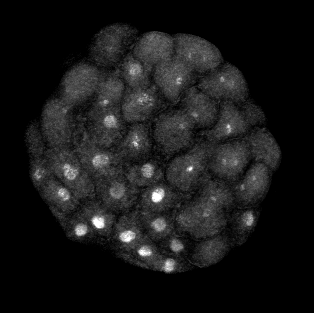

Supplement: Supplementary file 10 — Source Data for Figure 4 [file EMBJ-42-e112934-s007.zip › Fig.4/4A/rescue/R2/MAX_C2-EU_R2.tif]

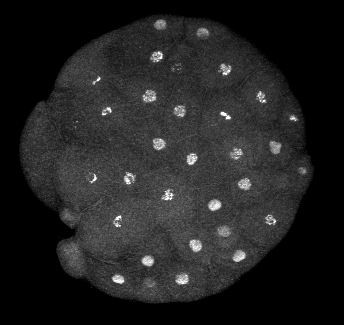

Supplement: Supplementary file 10 — Source Data for Figure 4 [file EMBJ-42-e112934-s007.zip › Fig.4/4A/shAlkbh1 injected/R1/MAX_C1-DAPI-sh1Injected1.tif]

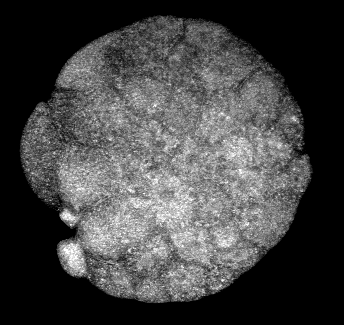

Supplement: Supplementary file 10 — Source Data for Figure 4 [file EMBJ-42-e112934-s007.zip › Fig.4/4A/shAlkbh1 injected/R1/MAX_C2-EU-sh1Injected1.tif]

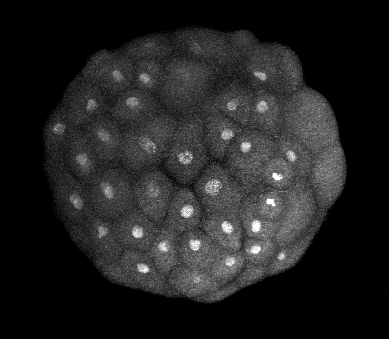

Supplement: Supplementary file 10 — Source Data for Figure 4 [file EMBJ-42-e112934-s007.zip › Fig.4/4A/shAlkbh1 injected/R2/MAX_C1-DAPI-sh1injected2.tif]

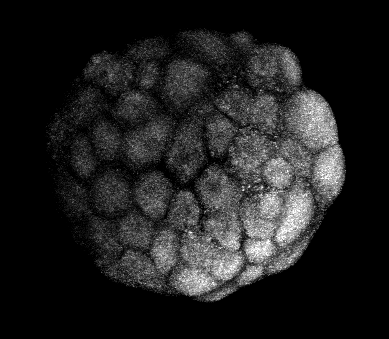

Supplement: Supplementary file 10 — Source Data for Figure 4 [file EMBJ-42-e112934-s007.zip › Fig.4/4A/shAlkbh1 injected/R2/MAX_C2-EU-sh1injected2.tif]

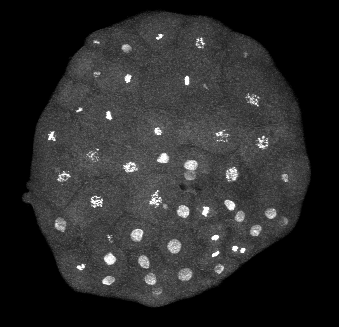

Supplement: Supplementary file 10 — Source Data for Figure 4 [file EMBJ-42-e112934-s007.zip › Fig.4/4A/shGfp injected/R1/MAX_C1-DAPI-shGfp_injected1.tif]

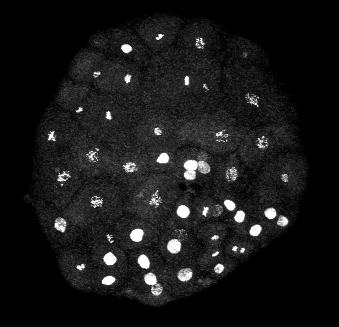

Supplement: Supplementary file 10 — Source Data for Figure 4 [file EMBJ-42-e112934-s007.zip › Fig.4/4A/shGfp injected/R1/MAX_C2-EU-shGfp_injected1.tif]

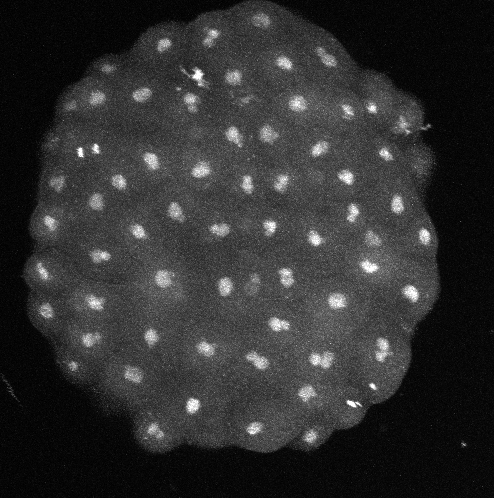

Supplement: Supplementary file 10 — Source Data for Figure 4 [file EMBJ-42-e112934-s007.zip › Fig.4/4A/shGfp injected/R2/MAX_C1-DAPI-shGFPinjected2.tif]

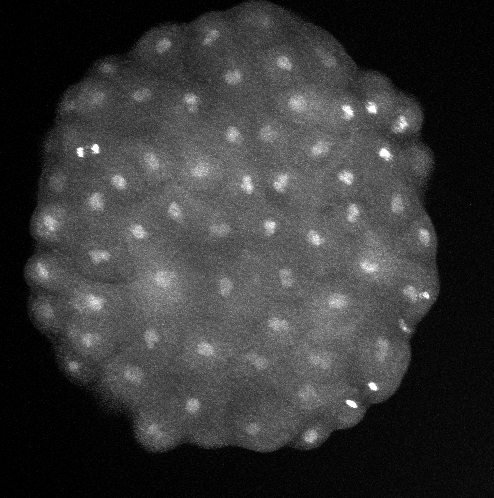

Supplement: Supplementary file 10 — Source Data for Figure 4 [file EMBJ-42-e112934-s007.zip › Fig.4/4A/shGfp injected/R2/MAX_C2-EU-shGFPinjected2.tif]

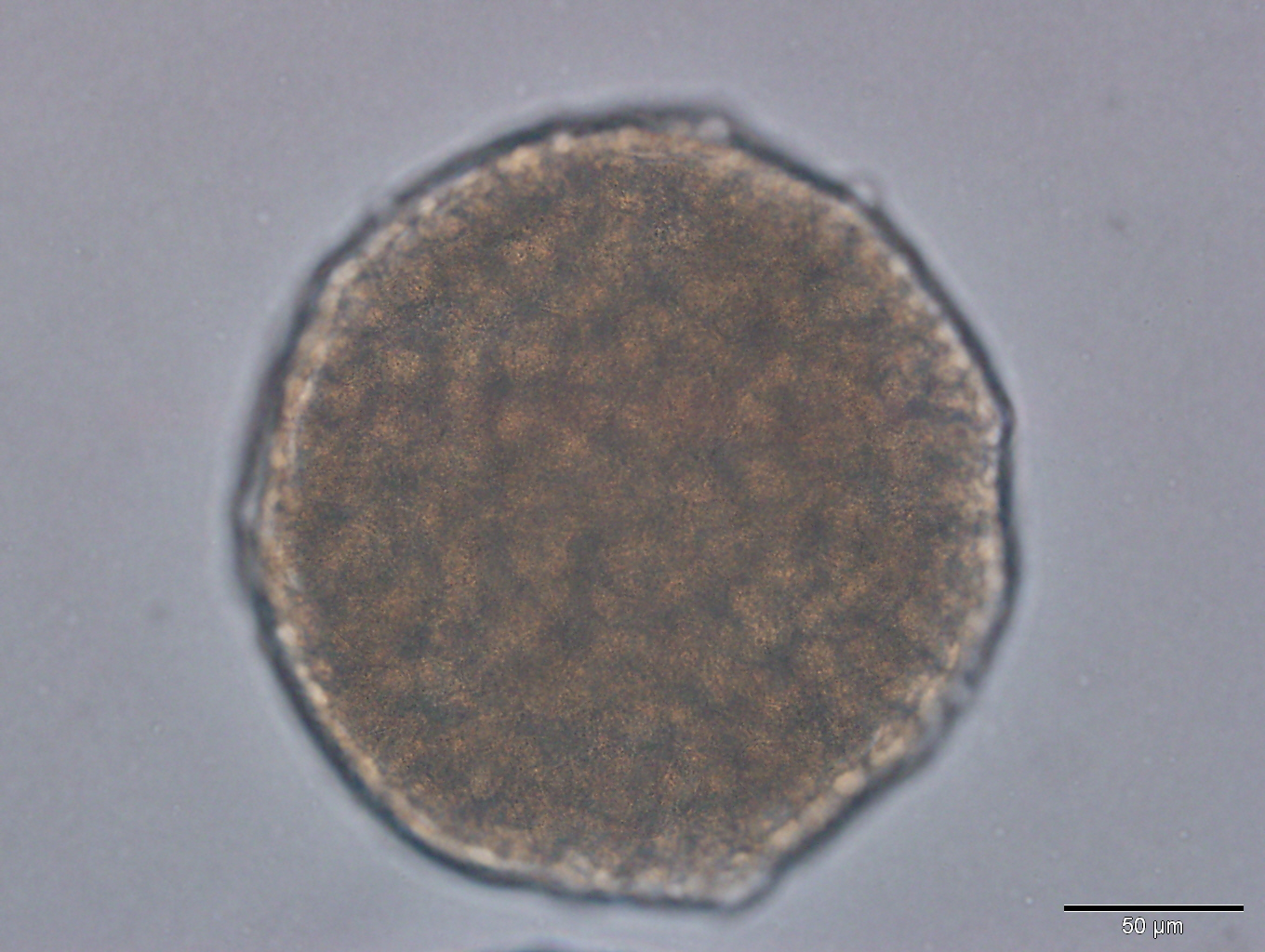

Supplement: Supplementary file 11 — Source Data for Figure 5 [file EMBJ-42-e112934-s012.zip › Fig.5/B/Negative/BF.tif]

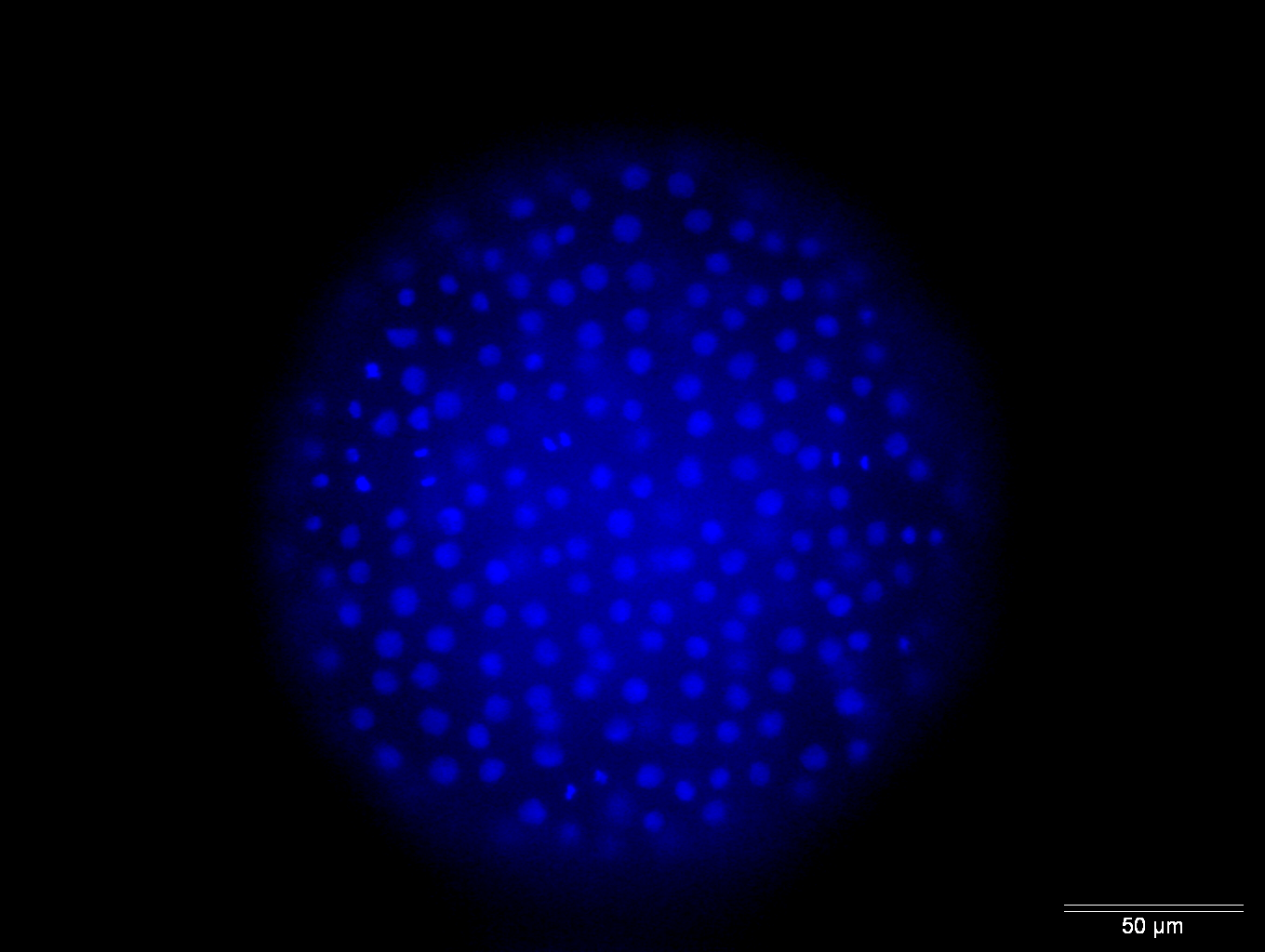

Supplement: Supplementary file 11 — Source Data for Figure 5 [file EMBJ-42-e112934-s012.zip › Fig.5/B/Negative/Hoescht.tif]

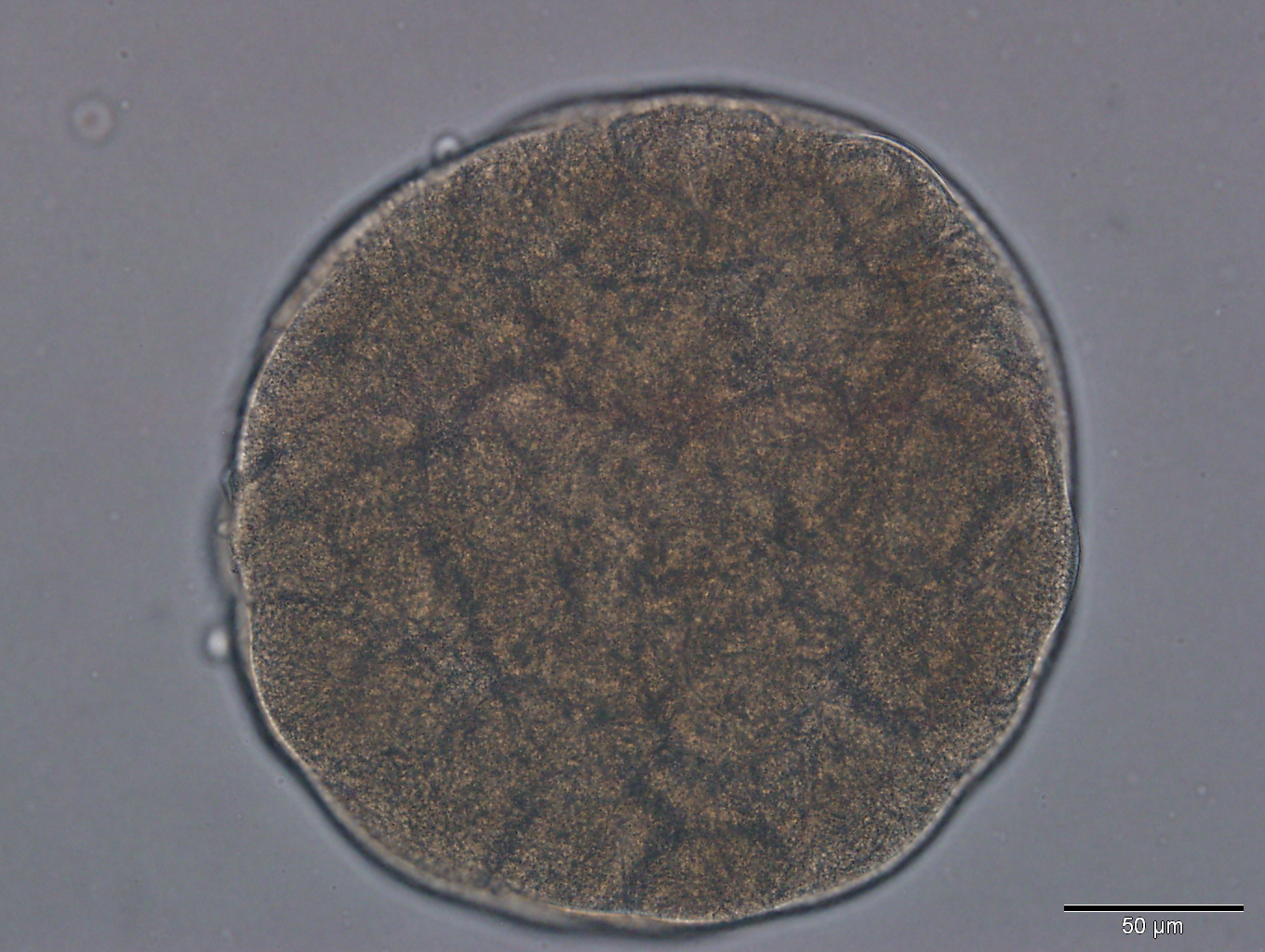

Supplement: Supplementary file 11 — Source Data for Figure 5 [file EMBJ-42-e112934-s012.zip › Fig.5/B/Treated/BF.tif]

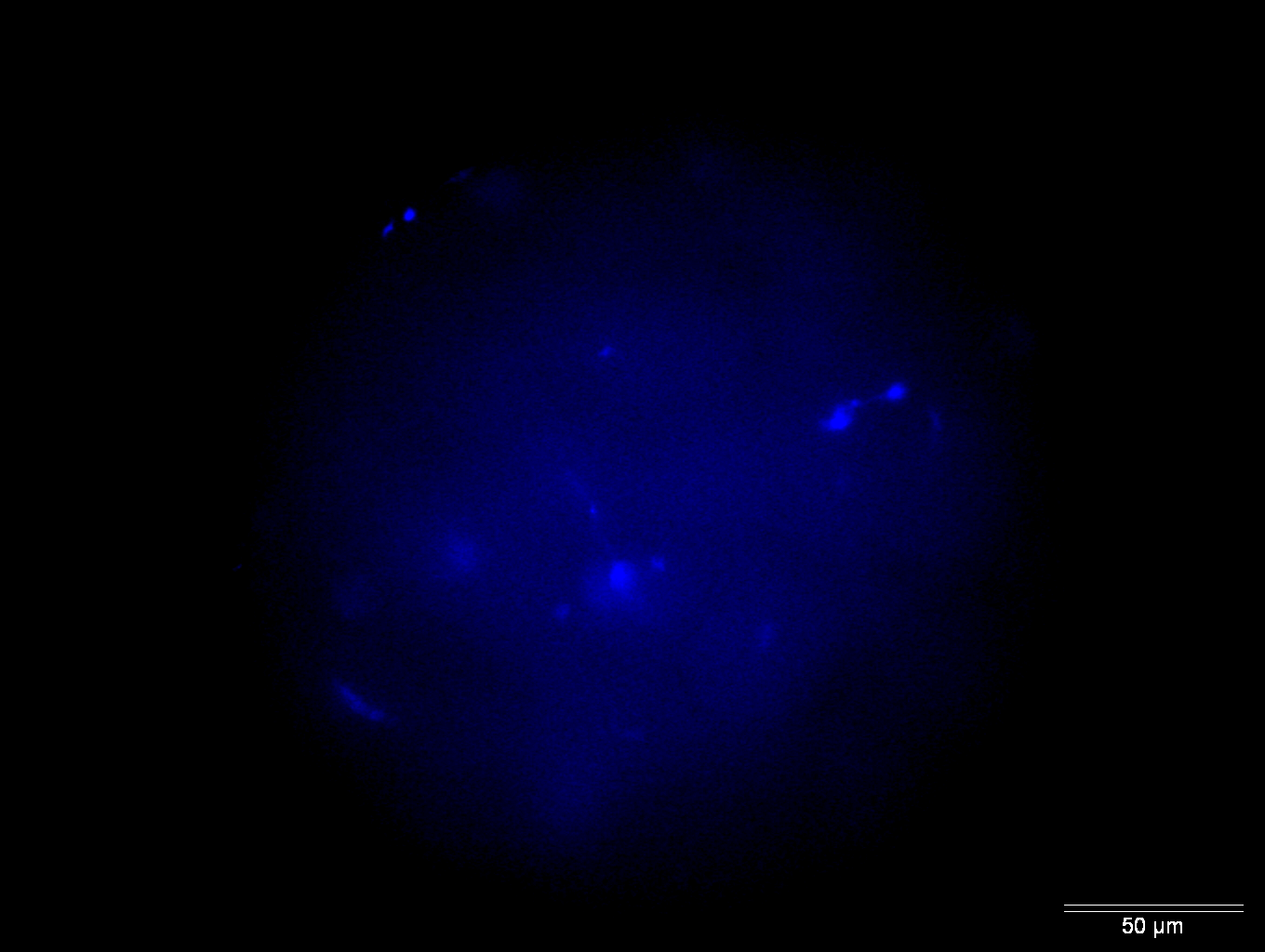

Supplement: Supplementary file 11 — Source Data for Figure 5 [file EMBJ-42-e112934-s012.zip › Fig.5/B/Treated/Hoescht.tif]

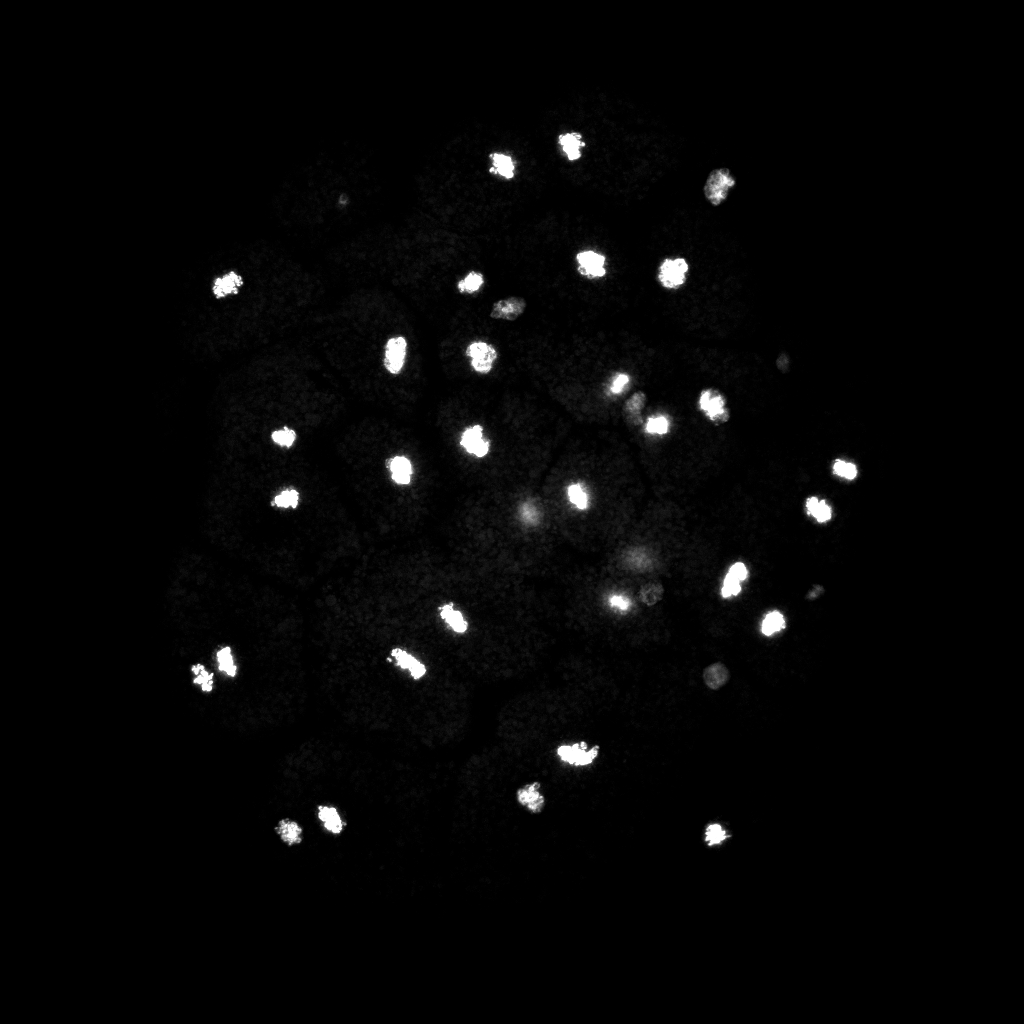

Supplement: Supplementary file 11 — Source Data for Figure 5 [file EMBJ-42-e112934-s012.zip › Fig.5/C/ATPinj/R1/MAX_C1-EdU_ATPinj_1.tif]

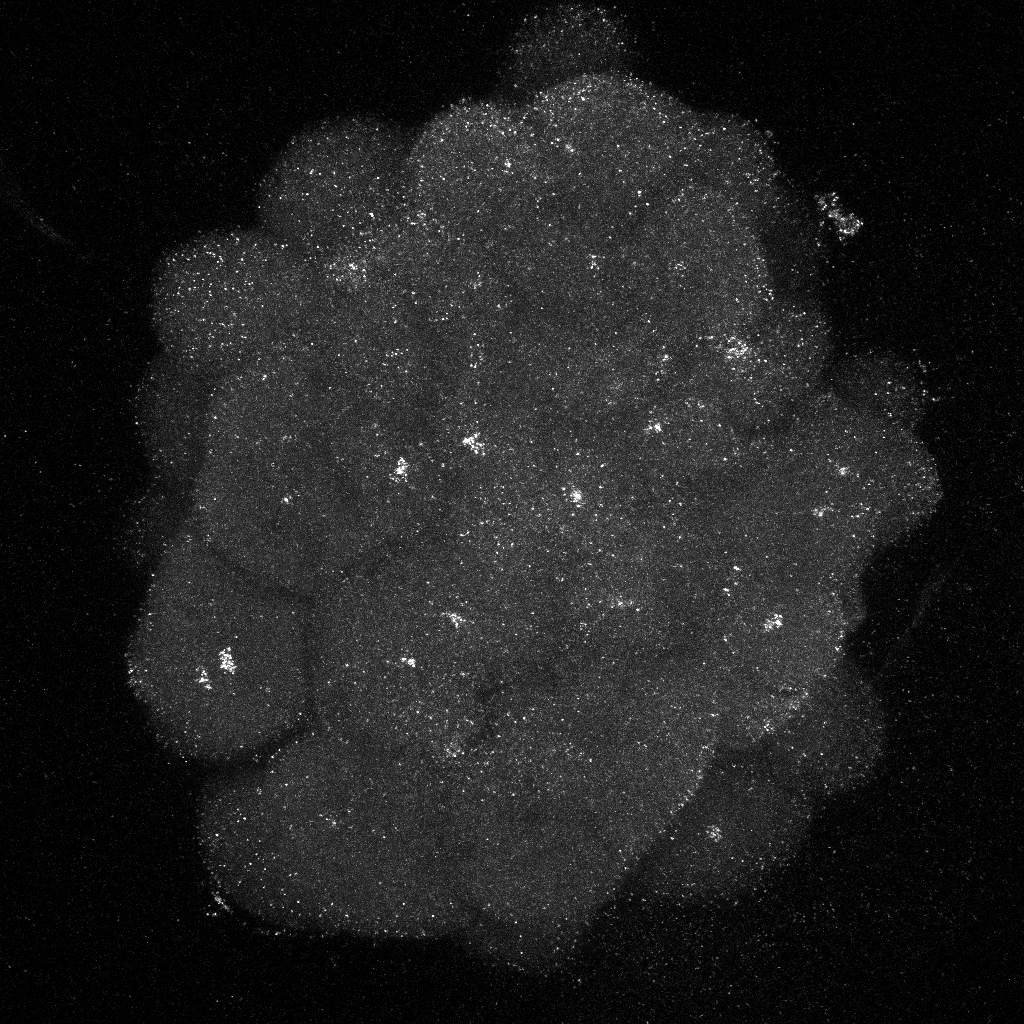

Supplement: Supplementary file 11 — Source Data for Figure 5 [file EMBJ-42-e112934-s012.zip › Fig.5/C/ATPinj/R1/MAX_C2_6mA_ATPinj_1.tif]

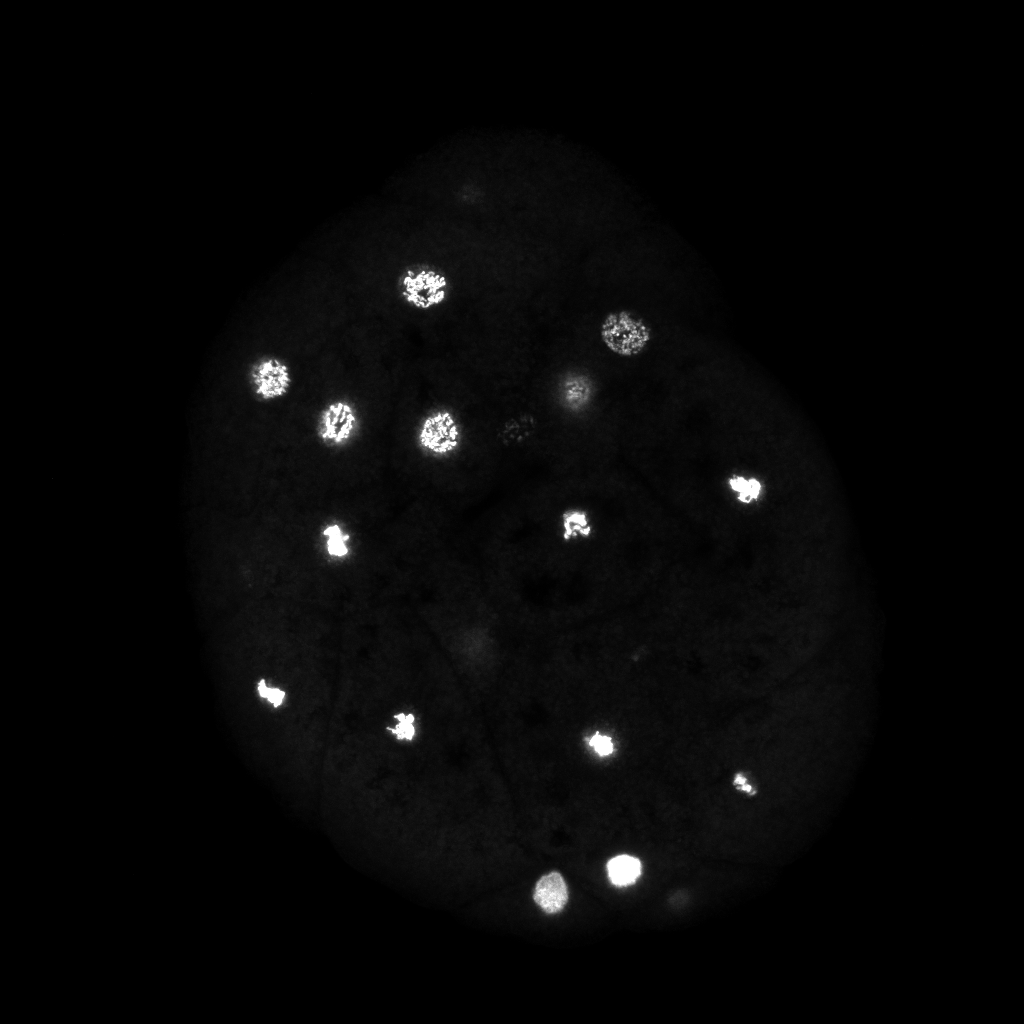

Supplement: Supplementary file 11 — Source Data for Figure 5 [file EMBJ-42-e112934-s012.zip › Fig.5/C/ATPinj/R2/MAX_C1-EdU_ATPinj_2.tif]

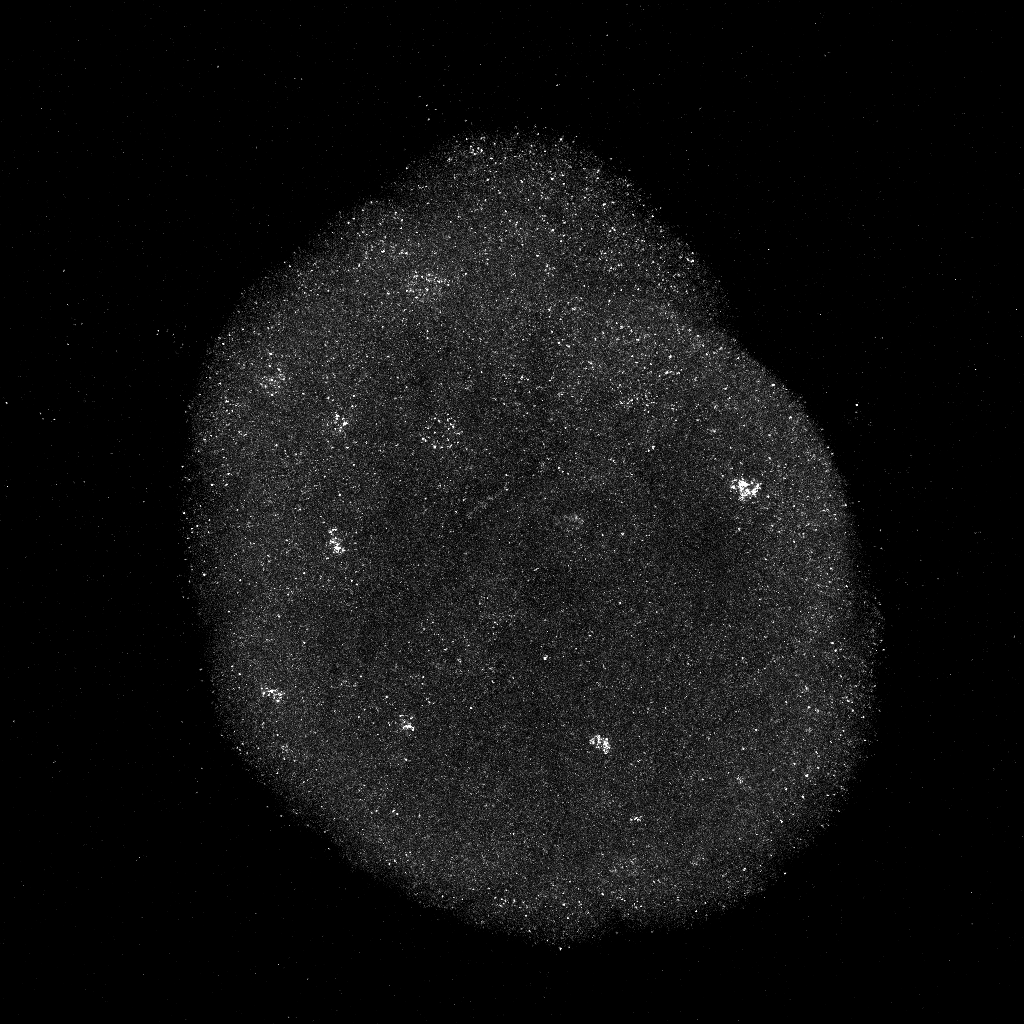

Supplement: Supplementary file 11 — Source Data for Figure 5 [file EMBJ-42-e112934-s012.zip › Fig.5/C/ATPinj/R2/MAX_C2-6mA_ATPinj_2.tif]

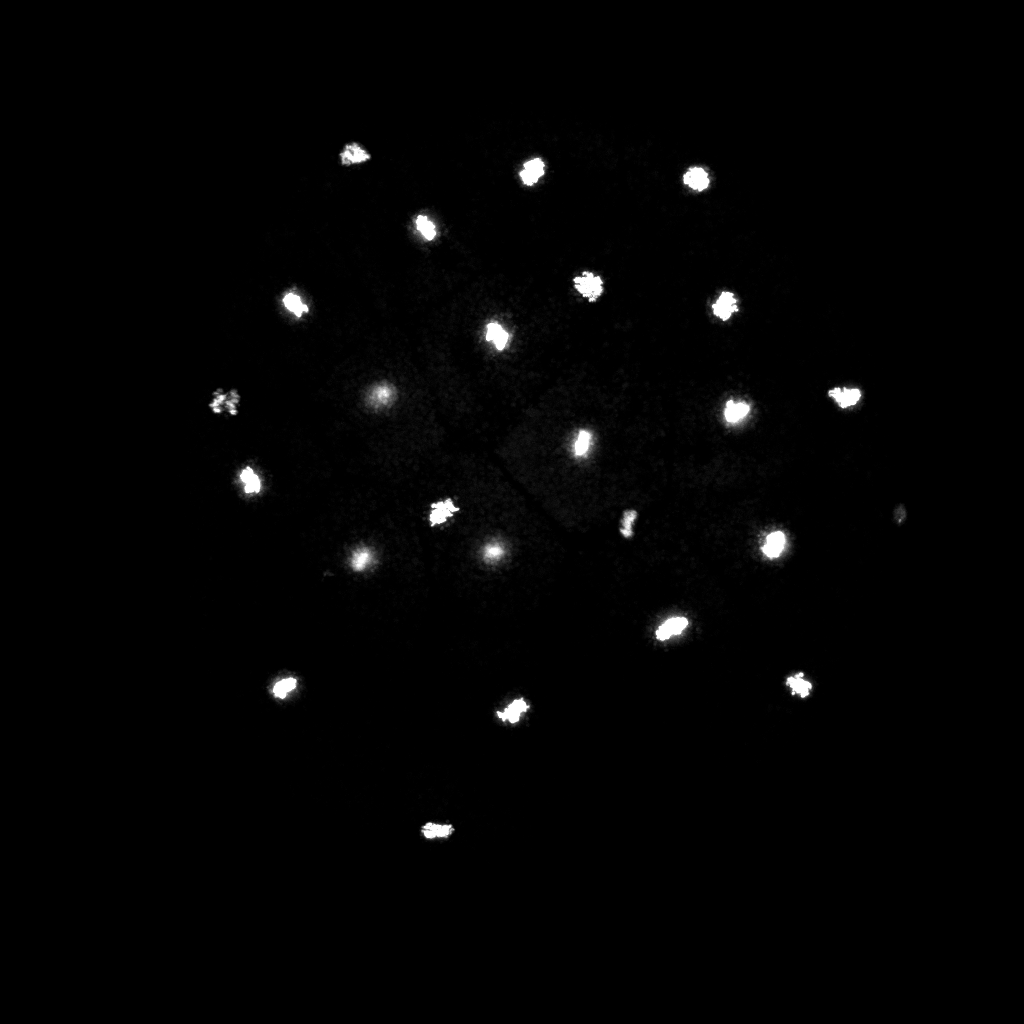

Supplement: Supplementary file 11 — Source Data for Figure 5 [file EMBJ-42-e112934-s012.zip › Fig.5/C/m6Ainj/R1/MAX_C1-EdU_m6Ainj_1.tif]

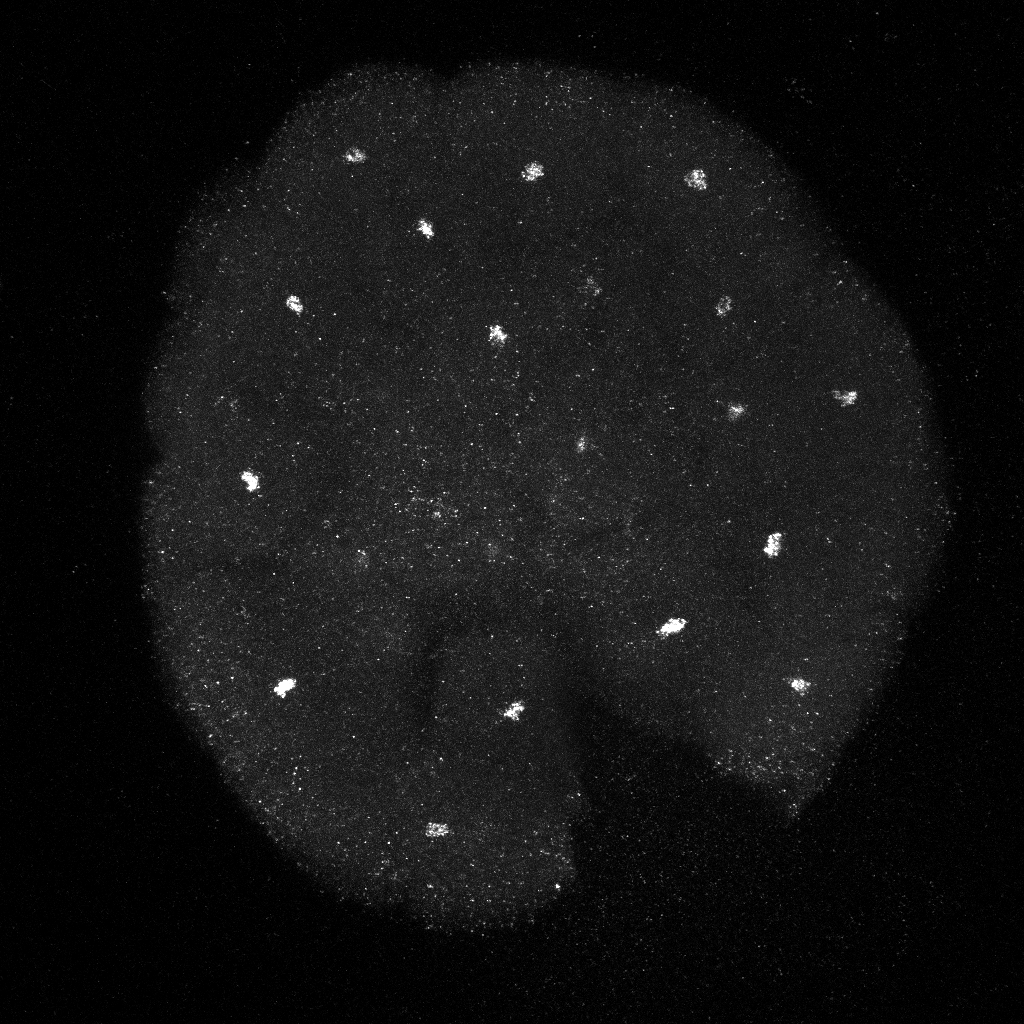

Supplement: Supplementary file 11 — Source Data for Figure 5 [file EMBJ-42-e112934-s012.zip › Fig.5/C/m6Ainj/R1/MAX_C2-6mA_m6Ainj_1.tif]

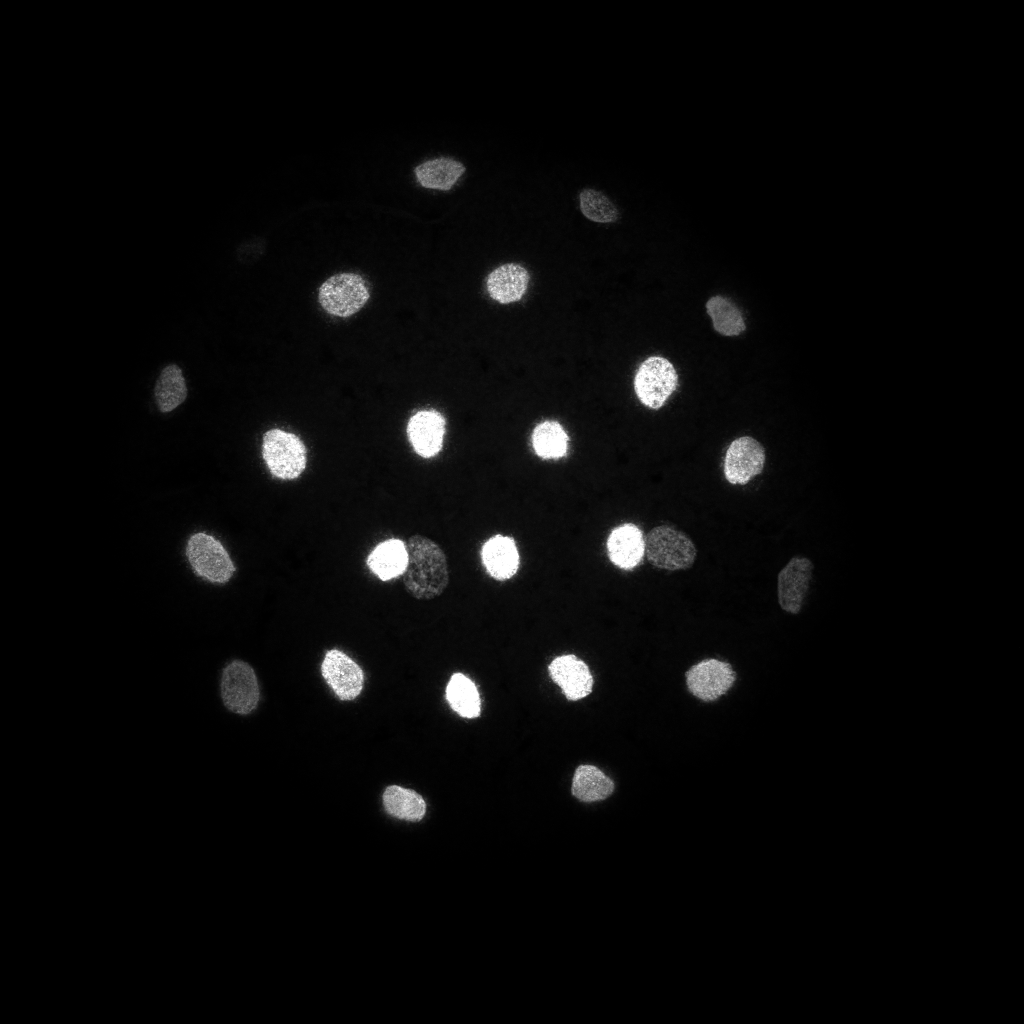

Supplement: Supplementary file 11 — Source Data for Figure 5 [file EMBJ-42-e112934-s012.zip › Fig.5/C/m6Ainj/R2/MAX_C1-EdU_m6Ainj_2.tif]

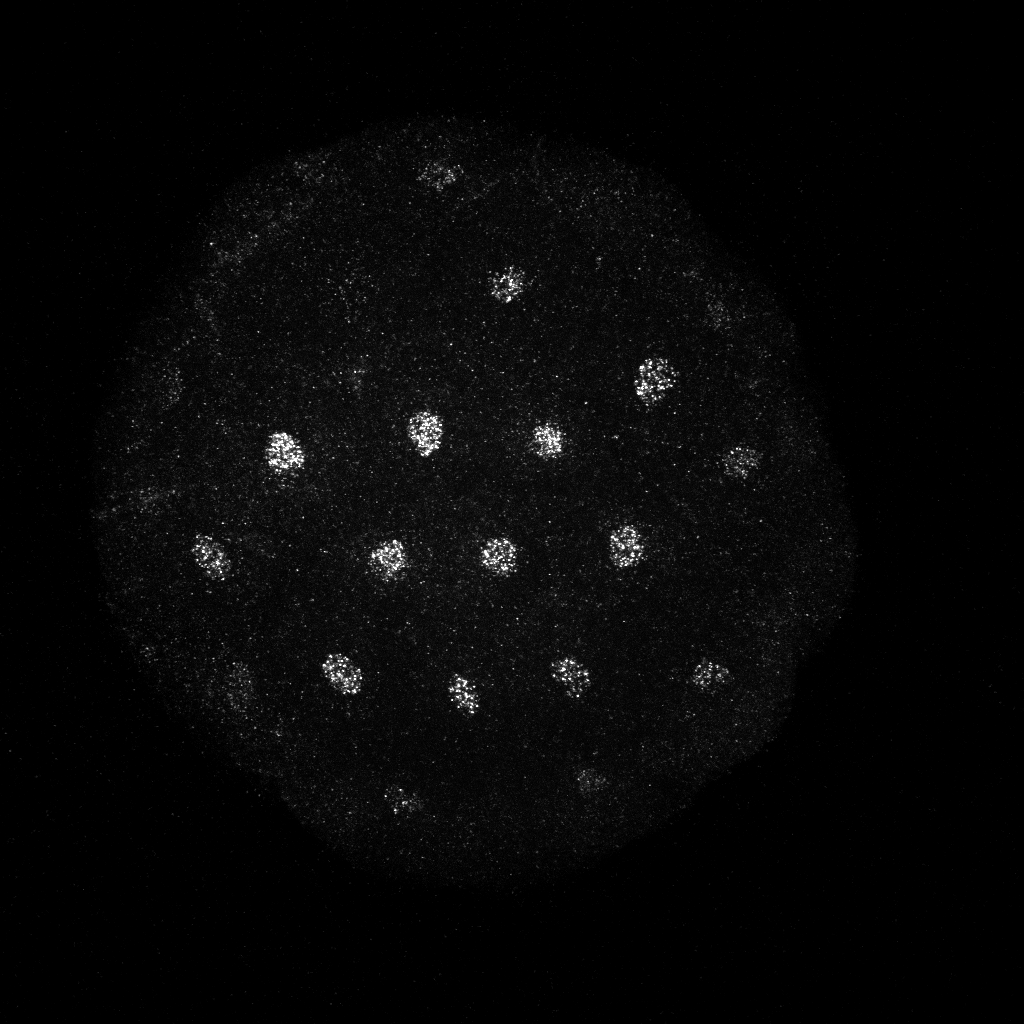

Supplement: Supplementary file 11 — Source Data for Figure 5 [file EMBJ-42-e112934-s012.zip › Fig.5/C/m6Ainj/R2/MAX_C2-6mA_m6Ainj_2.tif]

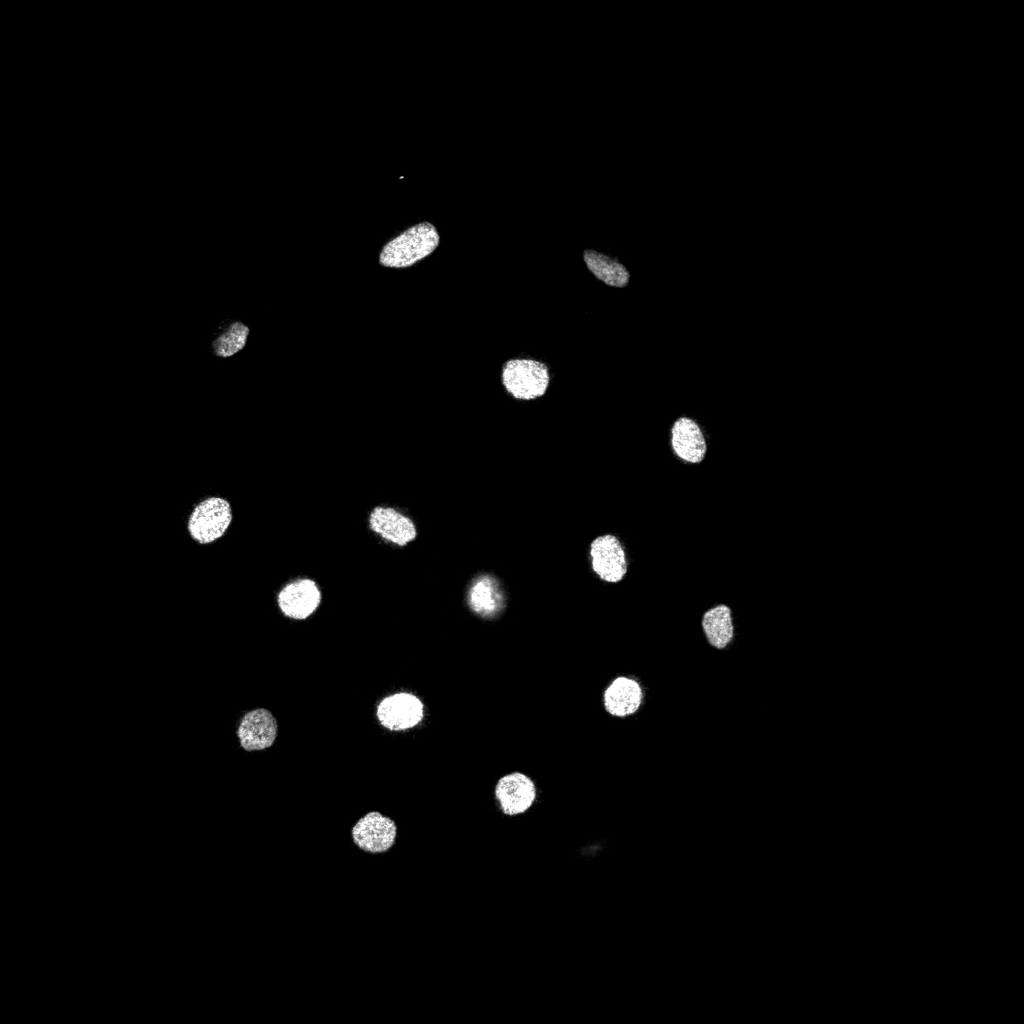

Supplement: Supplementary file 11 — Source Data for Figure 5 [file EMBJ-42-e112934-s012.zip › Fig.5/C/Uninjected/MAX_C1-uninj_1.tif]

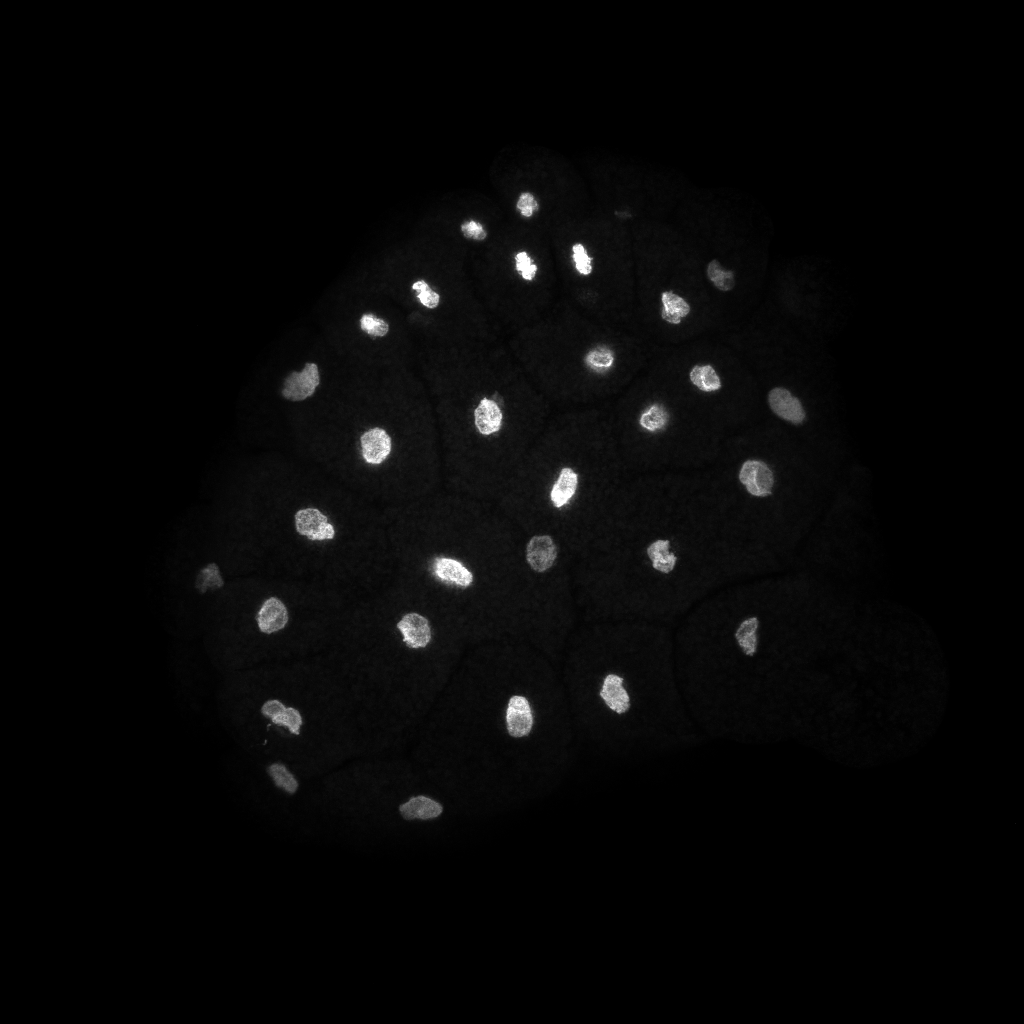

Supplement: Supplementary file 11 — Source Data for Figure 5 [file EMBJ-42-e112934-s012.zip › Fig.5/C/Uninjected/MAX_C1-uninj_2.tif]

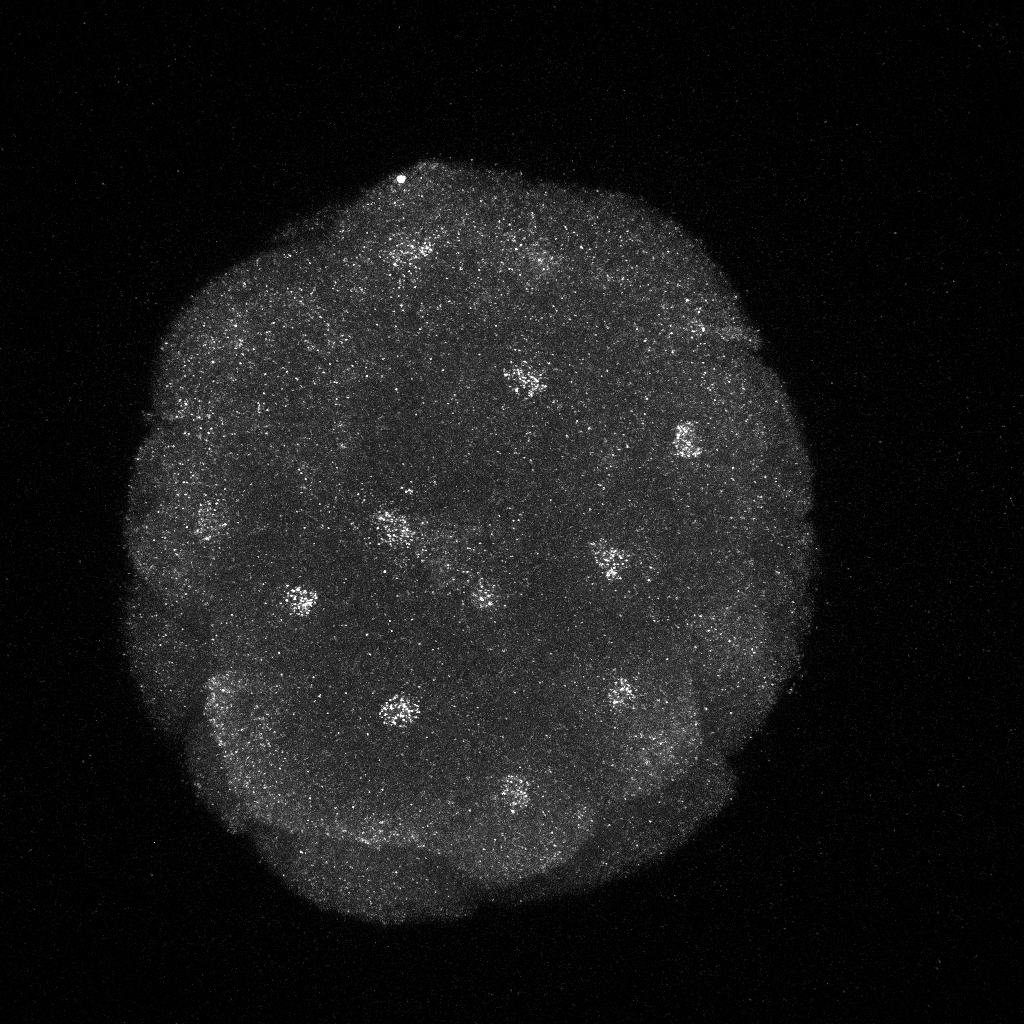

Supplement: Supplementary file 11 — Source Data for Figure 5 [file EMBJ-42-e112934-s012.zip › Fig.5/C/Uninjected/MAX_C2-uninj_1.tif]

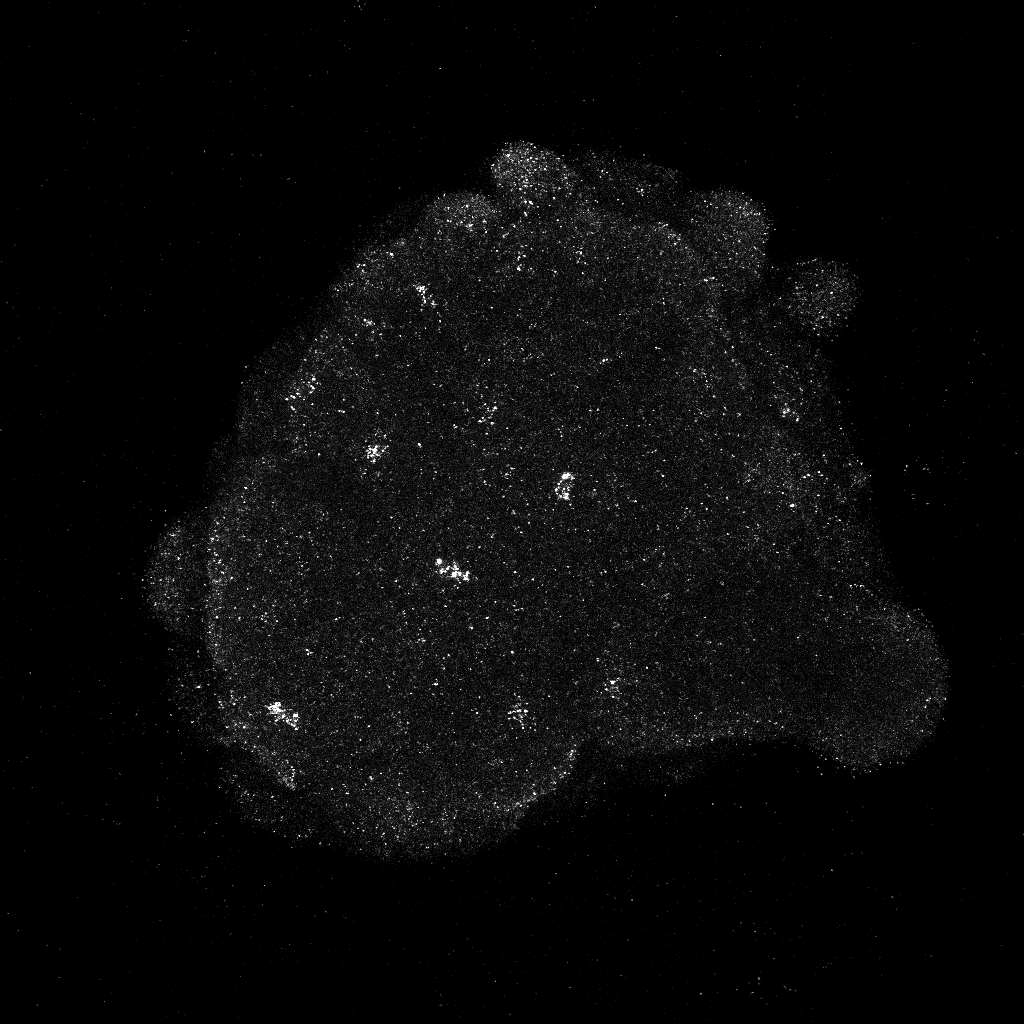

Supplement: Supplementary file 11 — Source Data for Figure 5 [file EMBJ-42-e112934-s012.zip › Fig.5/C/Uninjected/MAX_C2-uninj_2.tif]
