# Supplementary material for: Randomly incorporated genomic N6‐methyldeoxyadenosine delays zygotic transcription initiation in a cnidarian
Source: EMBO J. 2023 Jul 4;42(15):e112934. doi: 10.15252/embj.2022112934 (PMC10390872; doi:10.15252/embj.2022112934)
Supplement: Supplementary file 12 [file EMBJ-42-e112934-s006.pdf]

THE EMBO JOURNAL

Randomly incorporated genomic N6-methyldeoxyadenosine delays zygotic transcription initiation in a cnidarian

Febrimarsa Febrimarsa, Sebastian Gornik, Sofia Barreira, Miguel Salinas-Saavedra, Christine Schnitzler, Andreas Baxevanis, and Uri Frank
DOI: 10.15252/embj.2022112934

Corresponding author(s): Uri Frank (uri.frank@nuigalway.ie)

Review Timeline:

Transfer from Review Commons:	27th Oct 22
Editorial Decision:	28th Oct 22
Revision Received:	2nd Feb 23
Pre-decision consultation:	8th Mar 23
Preliminary Revision Plan:	21st Mar 23
Editorial Decision:	17th Apr 23
Revision Received:	17th May 23
Editorial Decision:	12th Jun 23
Revision Received:	14th Jun 23
Accepted:	16th Jun 23

Review COMMONS

Editor: Stefanie Boehm / Ieva Gailite

Transaction Report:This manuscript was transferred to The EMBO JOURNAL following peer review at Review Commons.

Review #1

1. Evidence, reproducibility and clarity:

Evidence, reproducibility and clarity (Required)

The main point of the current report is that 6mA is present in DNA of Hydractinia, and is introduced randomly into the genome by DNA polymerases, originating from degradation of maternally provided RNA via nucleotide salvage pathway. The authors observed that 6mA levels are changing over development and peak at 16-cell stage, with a sudden decrease to 'background levels' at 64 cell stage, a stage when zygotic genome gets activated. The 6mA drop is Alkbh1 dependent, since upon K/D of Alkbh1, 6mA levels were significantly higher than in control embryos. Authors also observed that AlkbH1 K/D delays zygotic genome activation (ZGA) to later stages, but without any noticeable consequences for the proper development. To demonstrate that 6mA is not controlled via direct DNA methylation, they show that K/D of two potential DNA methyl transferases N6amt1 and Mettl4 does not have any effect on 6mA levels. Supporting their hypothesis, authors demonstrate high activity and imperfect selectivity towards non-modified nucleotides of salvage pathway during embryo development using EU labeling experiments.

In general, the provided data support their model, however, the paper needs some improvements to include missing information and controls before publication.

Major comments:

1. Fig 1A shows a schematic where D3-6mA is added to only QTRAP but not QQQ experiment, usually QQQ methods also require isotopic standards for each component quantified to normalize for ionization differences and provide true quantitative information. Why did authors not use dA isotope? The ionization suppression is more pronounced at high concentrations of the components, which is true for dA in the current set up. How do authors control or at least test this? Fig S1 show that quantification works well, but were the total DNA amounts comparable to the gDNA amount used in actual samples? If yes, please indicate so.
2. In line 68 and in fig 1B, 1C there is a mysterious 'Neg. Ctrl 'sample. It is unclear what was the sample and more interestingly in fig 1B the levels in this sample are 0.015% but in fig 1C it is much below 0.001%. Why there is such a striking difference for the identical sample.
3. As I can see authors measured natural isotopologue of 6mA, however traces of contaminant bacterial DNA originating even from recombinant DNA degradation

enzymes also have 6mA, giving background signal. In their LC/MS experiments, did authors check if the 6mA comes truly from the gDNA and not from contaminant during DNA purification and processing before MS?
4. Fig 1D in the legend: authors should indicate that samples were already RNAse treated, and Line 80 in the text mentions a second RNase treatment (fig S1C) to confirm the specificity of the DNA staining.
5. In lines 86-87, authors compare the LC/MS and sequencing based quantifications, and say they are consistent. Can authors make a figure analogous to fig 1B but using sequencing data?
6. Fig 3B and 3C, controls showing the validity of EU staining, are required, such as RNAse treated sample with a signals disappearing; or control embryos without EU, thus having only background signal.
7. Fig 3D specificity control is missing, control embryos without EdU having only background signal.
8. Fig 4A legend: 'rescue solution (see text)'. Please describe in the legend what the solution was. Moreover, I did not find clear explanation in the text either, my only guess was from the materials in methods section, where authors used both shAlkbh1 and Alkbh1 mRNA with silent mutations.
9. Fig 4B shows many data points per condition and the legend says EU signals (in triplicate), was these triplicate animals with multiple cells, where EU signal from each cell was plotted as a point? Please specify in the legend.
10. Lines 169-170 state 'the lack of premature ZGA following N6amt1/Mettl4 knockdown (Figure S7B) indicate a lack of methyl transferase that maintains 6mA through embryogenesis' while an experiment indeed demonstrates that these are not the major players in this process, it does not prove these are not DNA methyl transferases. The absence of evidence is not the evidence of absence. I think authors should at least soften this conclusion.
11. Discussion section describes many experimental data that belong to Results section.
12. Fig S8 I think should be a part of the main figure since it is one of the important experiments to prove the high activity and somewhat low selectivity of salvage pathway in the embryos during the critical early stages.
13. Fig 5C the model is confusing, authors should improve it.
14. Fig S8 negative controls showing the specificity of CuAAC staining are missing: control animals/ embryos without EU.
15. Authors may find this reference useful: PMID: 32355286.
16. It is known that in mammals ADAL protein is the one which demethylates m6A nucleotide to clear it from the nucleotide pools and prevent it entering into the salvage pathway (PMID: 29884623). Does Hydractinia Symbiolongicarpus have an ADAL analog? If yes then it would be important to see if knock down/overexpression of this enzyme has any effect on the timing of ZGA. In principle, passively introduced 6mA

may be regulatory to proper time the ZGA, and is controlled via an activity of Adal and Alkbh1.
17. Material and methods are missing information:
- a. Line 370-371 provide references to the protocols listed or describe the steps.
- b. Line 373 standard column based purification protocol, what is it either explain or provide a reference.

Minor points:

Line 79 : 'Fig 1D and S1B', Did authors meant 'Fig1D and S1C'?

Fig 5A Y axis title is missing.

Line 379: 3D1-6mA should be D3-6mA please correct the other appearances as well.

Line 405: terms : dsDNA solutions and standard solutions are confusing please rephrase.

Line 410: Cleaned embryos, what does cleaned mean, be specific.

Line 413: PTx is mentioned, please explain what is it.

Line 415 and line 440 : HCl was washed and embryos were neutralized, I guess it should state : HCl was neutralized and embryos were washed with...'

Line 431: ' before fixed by incubation in PAGA-T..." did authors meant : 'before fixation with PAGA-T...?

Line 435: Permeabilization was done by further washes the fixed embryos with...", did authors meant: Permeabilization was done by an additional wash of the fixed embryos with...?

Line 440: The HCL was washed with what solution?

Line 446: For how long were the PTx washes?

Lines 458-460: the sentence is confusing.

Line 500: 'then used detect' should be 'then used to detect'

2. Significance:

Significance (Required)

There are many high profile papers describing the existence of 6mA in gDNA of different organism including insects and mammals. However, there is no proof that it has any biological function. Indeed, recent reports (PMID: 32355286 and 32203414) indicate that in mammalian cells, 6mA is indeed primarily incorporated by DNA polymerases and originates from a salvage pathway. The present report is the first in vivo evidence that confirms this to be the case more generally and, importantly, demonstrates a 6mA effect on ZGA. Hence, this is an important and timely report, which will be interesting to the field, as well as a broad audience to clarify the role of 6mA and the mechanism whereby it is introduced into gDNA.

My expertise: Biochemistry and biology of DNA and RNA modifications, including 6mA. Fair expertise: bioinformatics analysis.

3. How much time do you estimate the authors will need to complete the suggested revisions:

Estimated time to Complete Revisions (Required)

(Decision Recommendation)

Between 1 and 3 months

4. *Review Commons* values the work of reviewers and encourages them to get credit for their work. Select 'Yes' below to register your reviewing activity at Web of Science Reviewer Recognition Service (formerly Publons); note that the content of your review will not be visible on Web of Science.

Web of Science Reviewer Recognition

No

Review #2

1. Evidence, reproducibility and clarity:

Evidence, reproducibility and clarity (Required)

The manuscript reports developmental dynamics of DNA 6mA in the cnidarian Hydractinia symbiolongicarpus. The authors describe an event of a seemingly random accumulation of this DNA modification in 16-cell stage embryos of Hydractinia symbiolongicarpus followed by an apparent clearance of 6mA by the 64-cell stage. Interestingly, the depletion of cnidarian orthologue of the putative 6mA 'demethylase', Alkbh1, results in delay in zygotic transcription accompanied by high levels of DNA 6mA in 64-cell stage cnidarian embryos. The authors suggest that the 6mA they observe originates from random misincorporation of recycled degraded m6A-marked ribo-nucleotides during early cnidarian embryogenesis.

Overall, most of the experiments are performed at high technical level and the paper is generally nicely written. Despite this, in my opinion, the manuscript would benefit from incorporation of several addition controls and answering a number of points on the description/presenation of the data.

Major comments:

1. In the present version of the manuscript, the authors demonstrate the negative correlation between the presence of 6mA in the genome of cnidarian embryos and transcription. Although, the depletion of Alkbh1 leads to the delay in ZGA, strictly speaking, this effect may be independent of the catalytic function of Alkbh1. Therefore, to make a statement that m6A "random incorporation into the early embryonic genome inhibits transcription" the authors should use a catalytically inactive form of this enzyme as a control in the corresponding experiments and/or (ideally) perform in vitro transcription assays using 6mA-containing substrates.
2. Despite several experiments suggesting that random incorporation of recycled ribonucleotides occurs in cnidarian embryos, the source of 6mA in their DNA seems currently unclear. Would it be possible to directly test the author's hypothesis by comparing the levels of 6mA upon maternal (and possibly zygotic) depletion of the cnidarian orthologue of RNA m6A methyltransferase Mettl3 in cnidarian embryos? Alternatively, the authors could incubate the embryos in medium supplemented with labeled ribo-m6A followed by checking the levels of DNA 6mA in the embryonic DNA?

Minor comments:

1. It would be nice to complement Fig. 4, 5, and S7 with immunostaining of the corresponding embryos for 6mA.
2. The current Discussion contains references for several figures with experimental results. I suggest separating these experimental data from the Discussion. The authors should, in my opinion, make an additional Results chapter and, if possible, expand the Discussion section (that is currently minimal) speculating on significance of their results for different biological systems.
3. The present Title reads like a clear overstatement (at least currently, please see major comments above). The Title should also reference the organism where the observations have been made.

2. Significance:

Significance (Required)

The presence and significance of DNA 6mA in animal genomes is a very interesting and highly controversial topic. Although a number of studies suggest that relatively high levels of this DNA modification occur in multicellular eukaryotes in different biological/functional contexts, other reports challenged these observations attributing them to different experimental artifacts. In this context, the current paper that provides high quality novel experimental data on the developmental dynamics of DNA 6mA in cnidarian is extremely interesting and timely. Moreover, the author's results and the hypotheses on the function/origin of 6mA in cnidarian embryogenesis may provide a conceptual framework for the interpretation of other 6mA/m6A-related studies performed on different experimental models. Thus, this manuscript will definitely be of interest for a wide range of researchers working in the fields of epigenetics, cancer biology and developmental biology.
I strongly believe that this is an interesting and important study that definitely deserves to be published in a high impact journal.

3. How much time do you estimate the authors will need to complete the suggested revisions:

Estimated time to Complete Revisions (Required)

(Decision Recommendation)

Between 3 and 6 months

4. *Review Commons* values the work of reviewers and encourages them to get credit for their work. Select 'Yes' below to register your reviewing activity at Web of Science Reviewer Recognition Service (formerly Publons); note that the content of your review will not be visible on Web of Science.

Web of Science Reviewer Recognition

Yes

Revision Plan

Manuscript number: RC-2022-01616
Corresponding author(s): Uri Frank

[The "revision plan" should delineate the revisions that authors intend to carry out in response to the points raised by the referees. It also provides the authors with the opportunity to explain their view of the paper and of the referee reports.

The document is important for the editors of affiliate journals when they make a first decision on the transferred manuscript. It will also be useful to readers of the reprint and help them to obtain a balanced view of the paper.

If you wish to submit a full revision, please use our "Full Revision" template. ***It is important to use the appropriate template to clearly inform the editors of your intentions.****]*

1. General Statements [optional]

We appreciate the efforts the two reviewers had invested in reviewing our manuscript. Their constructive comments will help improve the paper overall.

2. Description of the planned revisions

Insert here a point-by-point reply that explains what revisions, additional experimentations and analyses are planned to address the points raised by the referees.

Revision Plan

Below is our point-by-point reply to the reviewers' comments.

Reviewer #1 (Evidence, reproducibility and clarity (Required)):

The main point of the current report is that 6mA is present in DNA of Hydractinia, and is introduced randomly into the genome by DNA polymerases, originating from degradation of maternally provided RNA via nucleotide salvage pathway. The authors observed that 6mA levels are changing over development and peak at 16-cell stage, with a sudden decrease to 'background levels' at 64 cell stage, a stage when zygotic genome gets activated. The 6mA drop is Alkbh1 dependent, since upon K/D of Alkbh1, 6mA levels were significantly higher than in control embryos. Authors also observed that AlkbH1 K/D delays zygotic genome activation (ZGA) to later stages, but without any noticeable consequences for the proper development. To demonstrate that 6mA is not controlled via direct DNA methylation, they show that K/D of two potential DNA methyl transferases N6amt1 and Mettl4 does not have any effect on 6mA levels. Supporting their hypothesis, authors demonstrate high activity and imperfect selectivity towards non-modified nucleotides of salvage pathway during embryo development using EU labeling experiments.
In general, the provided data support their model, however, the paper needs some improvements to include missing information and controls before publication.

Major comments:
1. Fig 1A shows a schematic where D3-6mA is added to only QTRAP but not QQQ experiment, usually QQQ methods also require isotopic standards for each component quantified to normalize for ionization differences and provide true quantitative information. Why did authors not use dA isotope? The ionization suppression is more pronounced at high concentrations of the components, which is true for dA in the current set up. How do authors control or at least test this?
We have limited resources of isotopic-labelled standards. Therefore, we initially used QQQ without these standards to obtain data that covered many time points in development to identify the general pattern and key time points of high and low 6mA. Once the QQQ indicated that the 16-cell stage has the highest 6mA and that this drops to background at the 64-cell stage (and remains so later on), we performed QTRAP with the isotope-labeled standard control only for these two stages. Looking at the data resulting from both techniques, it appears that they essentially revealed the same pattern. Since the main focus of the study is on 16- and 64-cell embryos, we feel that the contribution of performing all stages by QTRAP would be marginal. We have performed control experiments to assess ionization suppression for dA and found that it was insignificant. We will add the corresponding data to the Materials and Methods section.

Fig S1 show that quantification works well, but were the total DNA amounts comparable to the gDNA amount used in actual samples? If yes, please indicate so.
Yes, the amounts were the same (2 μg). We will change the methods sections accordingly.

Review COMMONS

2. In line 68 and in fig 1B, 1C there is a mysterious 'Neg. Ctrl 'sample. It is unclear what was the sample and more interestingly in fig 1B the levels in this sample are 0.015% but in fig 1C it is much below 0.001%. Why there is such a striking difference for the identical sample.
Negative controls were the same amounts (2 µg) of oligonucleotides without 6mA, DNAse-treated exactly like the samples. Figure 1B shows that QQQ is not sensitive enough to reliably detect 6mA concentrations below 0.02%, incapable to distinguish the background 6mA in the negative control from the level of 6mA in the 64-cell stage and later. Therefore, we utilized D3-6mA labelled QTRAP (Figure 1C) and determined that the level in the 64 cells stage embryos was actually ~0.01%. In the negative control, the amount was considerably lower, around 6 ppm (0.0006%).

3. As I can see authors measured natural isotopologue of 6mA, however traces of contaminant bacterial DNA originating even from recombinant DNA degradation enzymes also have 6mA, giving background signal. In their LC/MS experiments, did authors check if the 6mA comes truly from the gDNA and not from contaminant during DNA purification and processing before MS?
Yes, we did. As control for the level of 6mA contamination from the enzymatic digestion (sourced from bacteria), we also performed digestion of the negative control (see also answer to previous comment).

4. Fig 1D in the legend: authors should indicate that samples were already RNAse treated, and Line 80 in the text mentions a second RNase treatment (fig S1C) to confirm the specificity of the DNA staining.
The samples were indeed RNase-treated. We will modify the legend and the reference to figure 1D on line 80 accordingly.

5. In lines 86-87, authors compare the LC/MS and sequencing based quantifications, and say they are consistent. Can authors make a figure analogous to fig 1B but using sequencing data?
The data are already provided in Figure S1E. However, we used a Venn diagram to denote that these figures were generated by a different type of analysis (SMRT-sequencing as opposed to QTRAP). They are consistent but not identical.

6. Fig 3B and 3C, controls showing the validity of EU staining, are required, such as RNAse treated sample with a signals disappearing; or control embryos without EU, thus having only background signal.
Indeed, Fig 3C shows an RNase treated sample in which the EU signal is abolished as expected.

7. Fig 3D specificity control is missing, control embryos without EdU having only background signal.
The control is provided in Figure 3B. It shows a sample without EdU (treated with EU) and shows the background signal.

8. Fig 4A legend: 'rescue solution (see text)'. Please describe in the legend what the solution was. Moreover, I did not find clear explanation in the text either, my only guess was from the materials in methods section, where authors used both shAlkbh1 and Alkbh1 mRNA with silent mutations.

The reviewer is right, this was indeed the control that was used. We will modify the text to clarify this point.

9. Fig 4B shows many data points per condition and the legend says EU signals (in triplicate), was these triplicate animals with multiple cells, where EU signal from each cell was plotted as a point? Please specify in the legend.

Yes, triplicate embryos and each cell used as point. The legend will be adapted.

10. Lines 169-170 state 'the lack of premature ZGA following N6amt1/Mettl4 knockdown (Figure S7B) indicate a lack of methyl transferase that maintains 6mA through embryogenesis' while an experiment indeed demonstrates that these are not the major players in this process, it does not prove these are not DNA methyl transferases. The absence of evidence is not the evidence of absence. I think authors should at least soften this conclusion.

We agree and will tone down the relevant statement.

11. Discussion section describes many experimental data that belong to Results section.

This is a point also raised by Reviewer #2. We will move these points to the results and expand the discussion.

12. Fig S8 I think should be a part of the main figure since it is one of the important experiments to prove the high activity and somewhat low selectivity of salvage pathway in the embryos during the critical early stages.

We had originally left it out to save space. We prefer to leave this decision with the editor.

13. Fig 5C the model is confusing, authors should improve it.

It is difficult to describe a complex story using a single static model. Therefore, we will add an animation to the supplemental material to clarify the model.

14. Fig S8 negative controls showing the specificity of CuAAC staining are missing: control animals/ embryos without EU.

We will redo these experiments and include appropriate controls.

15. Authors may find this reference useful: PMID: 32355286.

We will add this ref.

16. It is known that in mammals ADAL protein is the one which demethylates m6A nucleotide to clear it from the nucleotide pools and prevent it entering into the salvage pathway (PMID:

29884623). Does Hydractinia Symbiolongicarpus have an ADAL analog? If yes then it would be important to see if knock down/overexpression of this enzyme has any effect on the timing of ZGA. In principle, passively introduced 6mA may be regulatory to proper time the ZGA, and is controlled via an activity of Adal and Alkbh1.
The gene is present in the Hydractinia genome. We could perform the experiments recommended. We will knock the gene down and look at the effect of this manipulation on ZGA.

17. Material and methods are missing information:
a. Line 370-371 provide references to the protocols listed or describe the steps.
b. Line 373 standard column based purification protocol, what is it either explain or provide a reference.
References will be provided.

Minor points:
Line 79 : 'Fig 1D and S1B', Did authors meant 'Fig1D and S1C'?
Fig 5A Y axis title is missing.
Line 379: 3D1-6mA should be D3-6mA please correct the other appearances as well.
Line 405: terms : dsDNA solutions and standard solutions are confusing please rephrase.
Line 410: Cleaned embryos, what does cleaned mean, be specific.
Line 413: PTx is mentioned, please explain what is it.
Line 415 and line 440 : HCl was washed and embryos were neutralized, I guess it should state : HCl was neutralized and embryos were washed with...'
Line 431: ' before fixed by incubation in PAGA-T..." did authors meant : 'before fixation with PAGA-T...?
Line 435: Permeabilization was done by further washes the fixed embryos with...", did authors meant: Permeabilization was done by an additional wash of the fixed embryos with...?
Line 440: The HCL was washed with what solution?
Line 446: For how long were the PTx washes?
Lines 458-460: the sentence is confusing.
Line 500: 'then used detect' should be 'then used to detect'
We will adopt all minor points above.

Reviewer #1 (Significance (Required)):

There are many high profile papers describing the existence of 6mA in gDNA of different organism including insects and mammals. However, there is no proof that it has any biological function. Indeed, recent reports (PMID: 32355286 and 32203414) indicate that in mammalian cells, 6mA is indeed primarily incorporated by DNA polymerases and originates from a salvage pathway. The present report is the first in vivo evidence that confirms this to be the case more

generally and, importantly, demonstrates a 6mA effect on ZGA. Hence, this is an important and timely report, which will be interesting to the field, as well as a broad audience to clarify the role of 6mA and the mechanism whereby it is introduced into gDNA.
My expertise: Biochemistry and biology of DNA and RNA modifications, including 6mA. Fair expertise: bioinformatics analysis.

Reviewer #2 (Evidence, reproducibility and clarity (Required)):

The manuscript reports developmental dynamics of DNA 6mA in the cnidarian Hydractinia symbiolongicarpus. The authors describe an event of a seemingly random accumulation of this DNA modification in 16-cell stage embryos of Hydractinia symbiolongicarpus followed by an apparent clearance of 6mA by the 64-cell stage. Interestingly, the depletion of cnidarian orthologue of the putative 6mA 'demethylase', Alkbh1, results in delay in zygotic transcription accompanied by high levels of DNA 6mA in 64-cell stage cnidarian embryos. The authors suggest that the 6mA they observe originates from random misincorporation of recycled degraded m6A-marked ribo-nucleotides during early cnidarian embryogenesis.
Overall, most of the experiments are performed at high technical level and the paper is generally nicely written. Despite this, in my opinion, the manuscript would benefit from incorporation of several addition controls and answering a number of points on the description/presenation of the data.
Major comments:

1. In the present version of the manuscript, the authors demonstrate the negative correlation between the presence of 6mA in the genome of cnidarian embryos and transcription. Although, the depletion of Alkbh1 leads to the delay in ZGA, strictly speaking, this effect may be independent of the catalytic function of Alkbh1. Therefore, to make a statement that m6A "random incorporation into the early embryonic genome inhibits transcription" the authors should use a catalytically inactive form of this enzyme as a control in the corresponding experiments and/or (ideally) perform in vitro transcription assays using 6mA-containing substrates.
We could perform shRNA-mediated Alkbh1 KD and try rescue ZGA by co-injecting a catalytically-inactive Alkbh1 mRNA.

The suggested in vitro experiment would be inconclusive for two reasons: first, Hydractinia polymerase may respond differently to 6mA; second, 6mA-mediated transcription inhibition could be indirect, requiring the in vivo context. We would like to add that transcription inhibition of 6mA has been demonstrated in vitro using yeast DNA polymerase as cited in the paper.

2. Despite several experiments suggesting that random incorporation of recycled ribonucleotides occurs in cnidarian embryos, the source of 6mA in their DNA seems currently

unclear. Would it be possible to directly test the author's hypothesis by comparing the levels of 6mA upon maternal (and possibly zygotic) depletion of the cnidarian orthologue of RNA m6A methyltransferase Mettl3 in cnidarian embryos? Alternatively, the authors could incubate the embryos in medium supplemented with labeled ribo-m6A followed by checking the levels of DNA 6mA in the embryonic DNA?
We show that maternal mRNAs are already methylated in the early embryo (Figure 5). Therefore, it would indeed make sense to ablate Mettl3 from the maternal tissue while maternal mRNAs are methylated. However, in the absence of a conditional knockout technique in Hydractinia, this would require generation of CRISPR-Cas9 mutants that would likely die early in their development, long before reaching sexual maturity. Instead, we are happy to perform the other experiment suggested by the reviewer to directly demonstrate m6A to 6mA transition.

Minor comments:
1. It would be nice to complement Fig. 4, 5, and S7 with immunostaining of the corresponding embryos for 6mA.
6mA immunostaning is not compatible with EU labeling because, first, they require different types of fixation (PAGA-T vs formaldehyde); second, immunostaining requires RNase treatment to remove m6A which would also remove the EU signal.

2. The current Discussion contains references for several figures with experimental results. I suggest separating these experimental data from the Discussion. The authors should, in my opinion, make an additional Results chapter and, if possible, expand the Discussion section (that is currently minimal) speculating on significance of their results for different biological systems.
This has also been requested by Reviewer #1. We will follow the reviewer's recommendation.

3. The present Title reads like a clear overstatement (at least currently, please see major comments above). The Title should also reference the organism where the observations have been made.
Following the revision, we believe that both random incorporation of 6mA and a delay in zygotic transcription will be well supported by our data. We will add the organism's name to the title as suggested.

Reviewer #2 (Significance (Required)):

The presence and significance of DNA 6mA in animal genomes is a very interesting and highly controversial topic. Although a number of studies suggest that relatively high levels of this DNA modification occur in multicellular eukaryotes in different biological/functional contexts, other reports challenged these observations attributing them to different experimental artifacts. In this context, the current paper that provides high quality novel experimental data on the

developmental dynamics of DNA 6mA in cnidarian is extremely interesting and timely. Moreover, the author's results and the hypotheses on the function/origin of 6mA in cnidarian embryogenesis may provide a conceptual framework for the interpretation of other 6mA/m6A-related studies performed on different experimental models. Thus, this manuscript will definitely be of interest for a wide range of researchers working in the fields of epigenetics, cancer biology and developmental biology.
I strongly believe that this is an interesting and important study that definitely deserves to be published in a high impact journal.

3. Description of the revisions that have already been incorporated in the transferred manuscript

Please insert a point-by-point reply describing the revisions that were already carried out and included in the transferred manuscript. If no revisions have been carried out yet, please leave this section empty.

4. Description of analyses that authors prefer not to carry out

Please include a point-by-point response explaining why some of the requested data or additional analyses might not be necessary or cannot be provided within the scope of a revision. This can be due to time or resource limitations or in case of disagreement about the necessity of such additional data given the scope of the study. Please leave empty if not applicable.

Reviewer #2 suggested four experiments, three of which are either impossible in our system or expected to reveal insignificant information. First, the reviewer suggests ablating Mettl3 from the maternal tissue. While being a good idea in principle, there is no conditional ablation technique available for Hydractinia. Generating CRISPR-Cas9 mutants would likely result in embryonic lethality, long before sexual maturation has been reached.

Second, the reviewer proposed to perform in vitro experiments with m6A-containing substrates. These experiments are unlikely to reveal useful data since the Hydractinia polymerase may respond differently to methylated adenine than commercially available polymerases. Also, transcription inhibition may be indirect, depending on the in vivo context that cannot be mimicked in vitro.

Finally, the reviewer suggested expressing a catalytically-dead Alkbh1 in the background of endogenous Alkbh1 knockdown to demonstrate that its function depends on the enzymatic activity to remove 6mA from the genome. While we could perform the

experiment (see our reply above), the information emanating from it would arguably be outside the scope of this study.

Dear Prof. Frank,

Thank you for transferring your manuscript from Review Commons to The EMBO Journal. I have now carefully read your study, as well as looked at the referee reports and your response, and discussed everything with the other members of the editorial team. Taking all into consideration, we have decided to invite a revision of the manuscript at The EMBO Journal.

In the revised version it will be important to resolve the referees' main concerns as outlined in your response to the Review Commons referee reports, and that the requested information on experimental procedures and controls is added. In particular, please address referee #2's points 1 and 2 experimentally. For point 1 this could be by adding the catalytic inactive Alkbh1 to a depleted background as suggested, or alternative means of demonstrating that Alkbh1's function in removing 6mA is required for ZGA, in turn supporting that 6mA must be removed for proper transcription. In addition, please also revise the manuscript and figures to address the more technical issues and questions the referees raise on specific points. When submitting the revised manuscript, please remember to include a detailed point-by-point response listing the individual referee comments and your response. The manuscript should then also fulfill all EMBO Journal formatting guidelines requirements when it is next submitted (please see below and: https://www.embopress.org/page/journal/14602075/authorguide#submissionofrevisions

Please note that it is our policy to allow only a single round of major revision. Acceptance depends on a positive outcome of a second round of review and therefore on the completeness of your responses included in the next, final version of the manuscript. Please contact us a soon as possible if you encounter any issues that would substantially affect the revision or if you have any questions on specific points.

At EMBO Press we ask authors to provide source data for the main and EV figures. Our source data coordinator will contact you to discuss which figure panels we would need source data for and will also provide you with helpful tips on how to upload and organize the files.

Please also feel free to contact me should you have any other further questions. Thank you for the opportunity to consider your work for publication, I look forward to receiving your revised manuscript.

Kind regards,
Stefanie Boehm

Stefanie Boehm
Editor
The EMBO Journal

-

***IMPORTANT NOTE: we now perform an initial quality control of all revised manuscripts before re-review. Your manuscript will FAIL this control and the handling will be DELAYED if the following APPLIES:

1) A data availability section is missing. (The section must be named "Data Availability" and must have the required format, detailed below). Please provide either a referee token or public access to all data.
2) Your manuscript contains error bars based on n=2. Please use scatter blots showing the individual datapoints in these cases. The use of statistical tests needs to be justified.

When submitting your revised manuscript, please carefully review the instructions that follow below. Failure to include requested items will delay the evaluation of your revision.***

When submitting your revised manuscript, please carefully review the instructions below and include the following items:

1) A .docx formatted version of the manuscript text (including legends for main figures, EV figures and tables). Please make sure that the changes are highlighted to be clearly visible.

2) Individual production quality figure files as .eps, .tif, .jpg (one file per figure). *Please remove figures from the main manuscript*

3) A .docx formatted letter INCLUDING the reviewers' reports and your detailed point-by-point response to their comments. As part of the EMBO Press transparent editorial process, the point-by-point response is part of the Review Process File (RPF), which will be published alongside your paper.

4) A complete author checklist, which you can download from our author guidelines (https://wol-prod-cdn.literatumonline.com/pb-assets/embo-site/Author%20Checklist%20-%20EMBO%20J-1561436015657.xlsx). Please insert information in the checklist that is also reflected in the manuscript. The completed author checklist will also be part of the RPF.

5) All corresponding authors are required to supply an ORCID ID for their name upon submission of a revised manuscript.

6) Before submitting your revision, primary datasets produced in this study need to be deposited in an appropriate public database. The required format for this section is:

Data availability

The datasets (and computer code) produced in this study are available in the following databases:

[data type]: [name of the resource] [accession number/identifier/doi] ([URL or identifiers.org/DATABASE:ACCESSION])

*** Note - All links should resolve to a page where the data can be accessed. ***

Please also see https://www.embopress.org/page/journal/14602075/authorguide#datadeposition Please note that the Data Availability Section is restricted to new primary data that are part of this study.

7) We replaced Supplementary Information with Expanded View (EV) Figures and Tables that are collapsible/expandable online (see examples in http://msb.embopress.org/content/11/6/812). A maximum of 5 EV Figures can be typeset. EV Figures should be cited as 'Figure EV1, Figure EV2" etc.. in the text and their respective legends should be included in the main text after the legends of regular figures.

- For the figures that you do NOT wish to display as Expanded View figures, they should be bundled together with their legends in a single PDF file called *Appendix*, which should start with a short Table of Content. Appendix figures should be referred to in the main text as: "Appendix Figure S1, Appendix Figure S2" etc. See detailed instructions regarding expanded view here: < https://www.embopress.org/page/journal/14602075/authorguide#expandedview>.

- Additional Tables/Datasets should be labeled and referred to as Table EV1, Dataset EV1, etc. Legends have to be provided in a separate tab in case of .xls files. Alternatively, the legend can be supplied as a separate text file (README) and zipped together with the Table/Dataset file.
** We suggest uploading table S1, S2, S3 as datasets. **

8) Our journal encourages inclusion of *data citations in the reference list* to directly cite datasets that were re-used and obtained from public databases. Data citations in the article text are distinct from normal bibliographical citations and should directly link to the database records from which the data can be accessed. In the main text, data citations are formatted as follows: "Data ref: Smith et al, 2001" or "Data ref: NCBI Sequence Read Archive PRJNA342805, 2017". In the Reference list, data citations must be labeled with "[DATASET]". A data reference must provide the database name, accession number/identifiers and a resolvable link to the landing page from which the data can be accessed at the end of the reference. Further instructions are available at < https://www.embopress.org/page/journal/14602075/authorguide#referencesformat >.

9) We would also encourage you to include the source data for figure panels that show essential data. Numerical data can be provided as individual .xls or .csv files (including a tab describing the data). For 'blots' or microscopy, uncropped images should be submitted (using a zip archive or a single pdf per main figure if multiple images need to be supplied for one panel).

Please note that source data should be uploaded in the following format:
a) For main figures: please upload the source data as one zip file per figure and label it as source_data_figX.
b) For EV figures please compile individual (zip) files for each figure, then combine these and upload one zip file for all figures. Please label this as source_data_EVfigures.
c) For Appendix figures please also make one file per figure, combine all Appendix files into one final zip file for all figures and label it as source_data_Appendix.

Additional information on source data and instruction on how to label the files are available at < https://www.embopress.org/page/journal/14602075/authorguide#sourcedata >.

10) When assembling figures, please refer to our figure preparation guideline in order to ensure proper formatting and readability in print as well as on screen:
https://wol-prod-cdn.literatumonline.com/pb-assets/embo-site/EMBOPress_Figure_Guidelines_061115-1561436025777.pdf

Please remember: Digital image enhancement is acceptable practice, as long as it accurately represents the original data and conforms to community standards. If a figure has been subjected to significant electronic manipulation, this must be noted in the figure legend or in the 'Materials and Methods' section. The editors reserve the right to request original versions of figures and the original images that were used to assemble the figure.

Further information is available in our Guide For Authors: https://www.embopress.org/page/journal/14602075/authorguide

****
We realize that it is difficult to revise to a specific deadline. In the interest of protecting the conceptual advance provided by the work, we recommend a revision within 3 months (26th Jan 2023). Please discuss the revision progress ahead of this time with the editor if you require more time to complete the revisions. Use the link below to submit your revision:

Link Not Available

****

***
Rev_Com_number: RC-2022-01616
New_manu_number: EMBOJ-2022-112934
Corr_author: Frank
Title: Randomly incorporated genomic 6mA delays zygotic transcription initiation

---

Reviewer #1 (Evidence, reproducibility and clarity (Required)):

The main point of the current report is that 6mA is present in DNA of Hydractinia, and is introduced randomly into the genome by DNA polymerases, originating from degradation of maternally provided RNA via nucleotide salvage pathway. The authors observed that 6mA levels are changing over development and peak at 16-cell stage, with a sudden decrease to 'background levels' at 64 cell stage, a stage when zygotic genome gets activated. The 6mA drop is Alkbh1 dependent, since upon K/D of Alkbh1, 6mA levels were significantly higher than in control embryos. Authors also observed that AlkbH1 K/D delays zygotic genome activation (ZGA) to later stages, but without any noticeable consequences for the proper development. To demonstrate that 6mA is not controlled via direct DNA methylation, they show that K/D of two potential DNA methyl transferases N6amt1 and Mettl4 does not have any effect on 6mA levels. Supporting their hypothesis, authors demonstrate high activity and imperfect selectivity towards non-modified nucleotides of salvage pathway during embryo development using EU labeling experiments.
In general, the provided data support their model, however, the paper needs some improvements to include missing information and controls before publication.

Major comments:
1. Fig 1A shows a schematic where D3-6mA is added to only QTRAP but not QQQ experiment, usually QQQ methods also require isotopic standards for each component quantified to normalize for ionization differences and provide true quantitative information. Why did authors not use dA isotope?

We were limited by the amount of isotope-labeled standards available. Therefore, we initially used QQQ without these standards to obtain data that covered many time points in development to identify the general pattern and key time points of high and low 6mA. Once established that the 16-cell stage has the highest 6mA and that this drops to background at the 64-cell stage (and remains so later on), we performed QTRAP with the isotope-labeled standard control only for these two stages. Looking at the data resulting from both techniques, it appears that they essentially revealed the same pattern; QTRAP is just more sensitive. Since the main focus of the study is on 16- and 64-cell embryos, we feel that the contribution of performing all stages by QTRAP would be marginal.

The ionization suppression is more pronounced at high concentrations of the components, which is true for dA in the current set up. How do authors control or at least test this?

We have performed control experiments to assess ionization suppression and found that it was insignificant. We have added the corresponding text to the Materials and Methods section.

Fig S1 show that quantification works well, but were the total DNA amounts comparable to the gDNA amount used in actual samples? If yes, please indicate so.

Yes, the amounts were the same (2 μg). We have changed the method sections accordingly.

2. In line 68 and in fig 1B, 1C there is a mysterious 'Neg. Ctrl 'sample. It is unclear what was the sample and more interestingly in fig 1B the levels in this sample are 0.015% but in fig 1C it is much below 0.001%. Why there is such a striking difference for the identical sample.

Negative controls were the same amounts (2 µg) of oligonucleotides without modified 6mA, DNAse-treated exactly like the samples (hence containing some background level of 6mA from the enzyme). Figure 1B indicates that QQQ is not sensitive enough to reliably detect 6mA concentrations below 0.02%, incapable to distinguish the background 6mA in the negative control from the level of 6mA in the 64-cell stage and later. Therefore, we utilized D3-6mA labelled QTRAP (Figure 1C) and determined that the level in the 64 cells stage embryos was actually ~0.01%. In the negative control, the amount was considerably lower, around 7 ppm (0.0007%).

3. As I can see authors measured natural isotopologue of 6mA, however traces of contaminant bacterial DNA originating even from recombinant DNA degradation enzymes also have 6mA, giving background signal. In their LC/MS experiments, did authors check if the 6mA comes truly from the gDNA and not from contaminant during DNA purification and processing before MS?

Indeed, the QQQ cannot discriminate between the background signal and the 6mA level at 64 cells; however, the QTRAP can (see Fig 1C). To clarify, as control for the level of 6mA contamination from the enzymatic digestion (sourced from bacteria), we also performed digestion of the negative control (see also answer to previous comment).

4. Fig 1D in the legend: authors should indicate that samples were already RNAse treated, and Line 80 in the text mentions a second RNase treatment (fig S1C) to confirm the specificity of the DNA staining.

The samples were indeed RNase-treated. We have modified the legend accordingly.

5. In lines 86-87, authors compare the LC/MS and sequencing based quantifications, and say they are consistent. Can authors make a figure analogous to fig 1B but using sequencing data?

The data are already provided in Figure S1E (now EV1C). However, we used a Venn diagram to denote that these figures were generated by a different type of analysis (sequencing as opposed to QTRAP). They are consistent but not identical. Using the same type of presentation may provide a false impression with the readers.

6. Fig 3B and 3C, controls showing the validity of EU staining, are required, such as RNAse treated sample with a signals disappearing; or control embryos without EU, thus having only background signal.

Indeed, Fig 3C (now Fig 3B bottom) shows an RNase treated sample in which the EU signal is abolished as expected.

7. Fig 3D specificity control is missing, control embryos without EdU having only background signal.

The control is now provided in Figure EV3D.

8. Fig 4A legend: 'rescue solution (see text)'. Please describe in the legend what the solution was. Moreover, I did not find clear explanation in the text either, my only guess was from the materials in methods section, where authors used both shAlkbh1 and Alkbh1 mRNA with silent mutations.

The reviewer is right. The text has now been modified to clarify this point.

9. Fig 4B shows many data points per condition and the legend says EU signals (in triplicate), was these triplicate animals with multiple cells, where EU signal from each cell was plotted as a point? Please specify in the legend.

The misunderstanding probably arose from the fact that we used each nucleus rather than each embryo as data point.

10. Lines 169-170 state 'the lack of premature ZGA following N6amt1/Mettl4 knockdown (Figure S7B) indicate a lack of methyl transferase that maintains 6mA through embryogenesis' while an experiment indeed demonstrates that these are not the major players in this process, it does not prove these are not DNA methyl transferases. The absence of evidence is not the evidence of absence. I think authors should at least soften this conclusion.

We agree; this phrase has been removed.

11. Discussion section describes many experimental data that belong to Results section.

This is a point also raised by Reviewer #2. We have now moved these points to the results and expanded the discussion.

12. Fig S8 I think should be a part of the main figure since it is one of the important experiments to prove the high activity and somewhat low selectivity of salvage pathway in the embryos during the critical early stages.

We had originally left it out to save space. It has now been moved to the Extended View figures (Fig EV3).

13. Fig 5C the model is confusing, authors should improve it.

We have modified the model (now Fig 5D) to improve clarity.

14. Fig S8 negative controls showing the specificity of CuAAC staining are missing: control animals/ embryos without EU.

Controls have been added to Fig EV3D as requested.

15. Authors may find this reference useful: PMID: 32355286.

We have added this ref.

16. It is known that in mammals ADAL protein is the one which demethylates m6A nucleotide to clear it from the nucleotide pools and prevent it entering into the salvage pathway (PMID: 29884623). Does Hydractinia Symbiolongicarpus have an ADAL analog? If yes then it would be important to see if knock down/overexpression of this enzyme has any effect on the timing of ZGA. In principle, passively introduced 6mA may be regulatory to proper time the ZGA, and is controlled via an activity of Adal and Alkbh1.

The gene is present in the Hydractinia genome. We have performed the experiments recommended and indeed found that Hydractinia Adal1 KD delays ZGA. These data have been added to the results (Fig EV3E).

17. Material and methods are missing information:
a. Line 370-371 provide references to the protocols listed or describe the steps.
b. Line 373 standard column based purification protocol, what is it either explain or provide a reference.

References now provided.

Minor points:
Line 79 : 'Fig 1D and S1B', Did authors meant 'Fig1D and S1C'?
Fig 5A Y axis title is missing.
Line 379: 3D1-6mA should be D3-6mA please correct the other appearances as well.
Line 405: terms : dsDNA solutions and standard solutions are confusing please rephrase.
Line 410: Cleaned embryos, what does cleaned mean, be specific.
Line 413: PTx is mentioned, please explain what is it.
Line 415 and line 440 : HCl was washed and embryos were neutralized, I guess it should state : HCl was neutralized and embryos were washed with...'
Line 431: ' before fixed by incubation in PAGA-T..." did authors meant : 'before fixation with PAGA-T...?
Line 435: Permeabilization was done by further washes the fixed embryos with...", did authors meant: Permeabilization was done by an additional wash of the fixed embryos with...?
Line 440: The HCL was washed with what solution?
Line 446: For how long were the PTx washes?
Lines 458-460: the sentence is confusing.
Line 500: 'then used detect' should be 'then used to detect'

All these points have been addressed.

Reviewer #1 (Significance (Required)):

There are many high profile papers describing the existence of 6mA in gDNA of different organism including insects and mammals. However, there is no proof that it has any biological function. Indeed, recent reports (PMID: 32355286 and 32203414) indicate that in

mammalian cells, 6mA is indeed primarily incorporated by DNA polymerases and originates from a salvage pathway. The present report is the first in vivo evidence that confirms this to be the case more generally and, importantly, demonstrates a 6mA effect on ZGA. Hence, this is an important and timely report, which will be interesting to the field, as well as a broad audience to clarify the role of 6mA and the mechanism whereby it is introduced into gDNA.
My expertise: Biochemistry and biology of DNA and RNA modifications, including 6mA. Fair expertise: bioinformatics analysis.

Reviewer #2 (Evidence, reproducibility and clarity (Required)):

The manuscript reports developmental dynamics of DNA 6mA in the cnidarian Hydractinia symbiolongicarpus. The authors describe an event of a seemingly random accumulation of this DNA modification in 16-cell stage embryos of Hydractinia symbiolongicarpus followed by an apparent clearance of 6mA by the 64-cell stage. Interestingly, the depletion of cnidarian orthologue of the putative 6mA 'demethylase', Alkbh1, results in delay in zygotic transcription accompanied by high levels of DNA 6mA in 64-cell stage cnidarian embryos. The authors suggest that the 6mA they observe originates from random misincorporation of recycled degraded m6A-marked ribo-nucleotides during early cnidarian embryogenesis.
Overall, most of the experiments are performed at high technical level and the paper is generally nicely written. Despite this, in my opinion, the manuscript would benefit from incorporation of several addition controls and answering a number of points on the description/presenation of the data.
Major comments:

1. In the present version of the manuscript, the authors demonstrate the negative correlation between the presence of 6mA in the genome of cnidarian embryos and transcription. Although, the depletion of Alkbh1 leads to the delay in ZGA, strictly speaking, this effect may be independent of the catalytic function of Alkbh1. Therefore, to make a statement that m6A "random incorporation into the early embryonic genome inhibits transcription" the authors should use a catalytically inactive form of this enzyme as a control in the corresponding experiments and/or (ideally) perform in vitro transcription assays using 6mA-containing substrates.

We have followed the reviewer's suggestion using a catalytically inactive mRNA in rescue experiments (called mutant rescue in Fig 4).

2. Despite several experiments suggesting that random incorporation of recycled ribonucleotides occurs in cnidarian embryos, the source of 6mA in their DNA seems currently unclear. Would it be possible to directly test the author's hypothesis by comparing the levels of 6mA upon maternal (and possibly zygotic) depletion of the cnidarian orthologue of RNA m6A methyltransferase Mettl3 in cnidarian embryos? Alternatively, the authors could incubate the embryos in medium supplemented with labeled ribo-m6A followed by checking the levels of DNA 6mA in the embryonic DNA?

We have flowed the reviewer's alternative suggestion, injecting m6ATP into zygotes. We find a higher level of 6mA at the 32-cell stage compared to unmethylated ATP injected embryos. These data are shown in Fig 5C.

Minor comments:
1. It would be nice to complement Fig. 4, 5, and S7 with immunostaining of the corresponding embryos for 6mA.

This is unfortunately impossible in Figs 4 & S7 (S7 is now EV2) due to the incompatibility of the fixative used in EU staining with immunostaining of 6mA. In Fig 5, the cell cycle in embryos treated with HU is arrested and comparison would be difficult due to aberrant nuclear morphology.

2. The current Discussion contains references for several figures with experimental results. I suggest separating these experimental data from the Discussion. The authors should, in my opinion, make an additional Results chapter and, if possible, expand the Discussion section (that is currently minimal) speculating on significance of their results for different biological systems.

This has also been requested by Reviewer #1. We have removed all new data to the results and generated a new discussion section.

3. The present Title reads like a clear overstatement (at least currently, please see major comments above). The Title should also reference the organism where the observations have been made.

Following the revision, we believe that both random incorporation of 6mA and a delay in zygotic transcription are well supported by our data. We have added the animal's genus name to the title as requested.

Reviewer #2 (Significance (Required)):

The presence and significance of DNA 6mA in animal genomes is a very interesting and highly controversial topic. Although a number of studies suggest that relatively high levels of this DNA modification occur in multicellular eukaryotes in different biological/functional contexts, other reports challenged these observations attributing them to different experimental artifacts. In this context, the current paper that provides high quality novel experimental data on the developmental dynamics of DNA 6mA in cnidarian is extremely interesting and timely. Moreover, the author's results and the hypotheses on the function/origin of 6mA in cnidarian embryogenesis may provide a conceptual framework for the interpretation of other 6mA/m6A-related studies performed on different experimental models. Thus, this manuscript will definitely be of interest for a wide range of researchers working in the fields of epigenetics, cancer biology and developmental biology.
I strongly believe that this is an interesting and important study that definitely deserves to be published in a high impact journal.

Dear Dr. Frank,

Thank you for submitting your revised Review Commons manuscript to The EMBO Journal. We have now received comments from one of the original reviewers, which are included below for your information.

As you will see from the reports, the reviewer finds that several of their initial comments are not addressed satisfactorily. Furthermore, they also raise additional experimental points that they find would need to be addressed to consider publication in The EMBO Journal. I realise that these points were not raised at Review Commons and reflect the re-adjustment by the reviewer to the level of insight expected for publication here.

Since several points indicated by the referees ask for additional experimentation and may not be easily addressable, I would appreciate if you could take a look at the reviewer comments and let me know to what extent you would be prepared to address the raised issues in a second revision round. A brief preliminary point-by-point response to the reviewer comments would be very helpful for the final editorial decision.

Please feel free to contact me if you have any questions regarding this pre-decision consultation approach. I look forward to your response.

With best regards,

Ieva

Ieva Gailite, PhD
Senior Scientific Editor
The EMBO Journal
Meyerhofstrasse 1
D-69117 Heidelberg
Tel: +4962218891309
i.gailite@embojournal.org

Referee #1 (Report for Author)

While I appreciate that the authors have made efforts to revise the ms, it unfortunately still requires major revision. The conclusions/assumptions the authors make are not fully supported by the data presented, and for some reviewer's comments the authors failed to provide satisfactory answers.

Major points
1. Authors failed to provide satisfactory answers to reviewer's comments:
a. Response to reviewer 1 comment 1: The experiments that confirm no ionization suppression were the ones described in lines 266-269, however the amounts of dA used in this experiment is 4 pmol s= 4x10-12 moles ,which is more than 100 times lower than the ones used in gDNA samples. Assuming average Mw of nucleotide in DNA is 310 g/mol, and dA represents~ 25 % of DNA sequence content, one can estimate that 2 μg of DNA roughly contains ~ 1.6 x10-9 moles or 1.6 nmoles of dA (2×10-6(g)/310 (g/mol)/4).

Additionally I disagree with an argument that dA isotopes are too difficult/expensive, a variety of dA with a different combination of isotope atoms are commercially available from several companies. (e.g https://www.silantes-shop.com). The commercial dA nucleoside isotopic standard can be added after DNA digest, to all samples including standards.
Lines 268 says that 4 pmol dA is spiked in into 'biological samples', but what are these biological samples is not clear. If these are the 2 µg of gDNA then 4 pmols of dA spike in will be 100x lower then dA present in gDNA samples, which makes no sense.

b. Response to reviewer 1 comment 2 : In lines 76-77, authors observe that a measurement of an identical control sample (Fig 1 B and C 'CTRL(-)') with QQQ instrument returns values 10 x greater then Q-Trap, and authors still conclude that Q-trap results confirm that QQQ measurements are all correct. An identical control sample must contain identical levels of m6A, thus if both methods work, the measured values must be similar. Author's arguments that this difference can be explained by difference in sensitivity is wrong. Poor sensitivity will just not be able to detect any signal, and the values will be missing.
The only conclusion from the observed results, which can be made, is that QQQ measurements are erroneous and thus misleading, and should be optimized to either solve this inconsistency or be replaced with q-trap experiments.
c. Response to Reviewer 2 minor comment 1: states that labeling of 6mA and EU is impossible for identical samples due to incompatibility of the techniques. However, such staining can be performed in parallel on two identical samples. Alternatively, authors could provide LC/MS based quantifications of 6mA instead in analogous samples.
2. Authors should not assign the processes to a precise stage, since experiments performed only at selected stages with intermediate stages being omitted.

a. Lines 125-126: using results of fig 3B where two stages are shown, '16-cell' and '64-cell', authors conclude that transcriptional wave is starting at 64-cell stage. Stage '32- cell' is not shown, thus authors must consider that the wave may start at '32-cell' stage as well.
b. Lines 136-138: discusses that EU incorporation was delayed upon shAlkbh1, but they only show stage 512-cell and 64-cell. It would be beneficial to also include intermediate stages 128- and 256-cell and ideally even stages 16- and 32-cell. Additionally the two panels from Fig 2-A and EV2-A must be shown in one. Considering the conclusion authors made, that should be limited to only editorial edits.

3. Authors conclusions are not fully supported with the data provided:

a. Line 139 says that results from EU incorporation suggest that 6mA levels follow EU patterns (linked to zygotic genome activation). This is just a hypothesis, moreover, Fig EV2C is confusing, the bars are only hypothetical. The effects of Alkbh1 on 6mA levels must be confirmed experimentally using LC/MS.
b. Lines 152-163: Authors performed the knock down of N6amt1 and Mettl4 and observe no zygotic genome activation by EU incorporation (fig EV2D), then they conclude that these enzymes do not deposit 6mA.
First, stage is not indicated; Second, for the shMettl4 sample, EU signal is present, while for the shN6AMT1 sample the EU signal is absent. The effects of each protein knockdowns are not identical on EU level, how authors arrive at the conclusion above is mysterious.
Authors should show EU labeling for different stages to clearly demonstrate when EU signal is absent and when it is presen. Also a stage at which ZGA starts in each treatment will be clearly identifiable. Something like from 16-cell stage - to a stage when all samples show EU signal. Moreover, authors must include shGFP or another non-targeting control.
To make any conclusion about the effect of the two above mentioned proteins on 6mA levels in DNA, authors must provide 6mA LC/MS measurements for all stages, which will be shown for EU labeling.
c. Line 170-172 authors measure m6A levels in maternally provided RNA in the embryos; observing a slight decrease in m6A levels authors conclude that RNA is degraded between stages 2-cell and 16-cell, (fig5A), it is an overstatement. For instance, methyl groups from m6A could be removed RNA by Alkbh5 or Alkbh1 on otherwise intact RNA.
d. Fig. EV3E: Knock down of Adal shows decrease of EU incorporation at 64-cell stage. Again controls showing proper development are missing, as well as stages before and after 64 cell stage, till the stage where EU signal appears in all samples. Apart from that, 6mA levels in DNA need to be determined by LC/MS to confirm that Adal knock down indeed causes 6mA levels increase in DNA.

4. The model shown on fig 4C is confusing, what does Alkbh1 do at stage 16? Why at stage 16 m6A is present and at stage 64 is almost absent except the two sites bound (protected?) by Alkbh1?

Model shown on fig 5d is also confusing and why Alkbh1 is absent here at stage 16, what is the difference between stage 16 on the models from fig 4C and 5D?

Moreover, authors should only present single model and show all they want clearly on that drawing, and explain all that explicitly in figure legend.

Model in fig 5D states that RNR is activated at 8-cell stage, how do authors know this? HU experiments that indicate that HU stops development at stage 8 is not related to the time when HU is activated. It just shows that dNTP pools reach critical levels.

Minor points:

1. 'block-i1 solution' terminology appears in line 287 but composition :(3% BSA and 0.25% Triton-X in PBS) is listed much later in line 314. Composition must be described at the first instance it appears in text.
2. Figures in general : Embryonic stages are sometimes labeled as 'c' or 'cell' on different figures, authors should be consistent
3. Fig 4C authors must indicate that each spot corresponds to single nucleus and state how many different embryos/slides were used, that information must be included directly in the figure legend.
Fig 5A: Y-axis says m6A/A in arb units, why then the % is used, it is confusing since it assumes % of m6A over A, which is not the case for that measurement thus usage of % in this case should be avoided.
4. Fig EV1A: 'variable standard' term is confusing, call it for instance 'expected '
5. Fig EV2C: All of the bars must be labeled not only 16c.
6. Line 140: Albh1 should be Alkbh1
7. Line 249: 'chilled in ice' should be 'chilled on ice'

8. Lines 369-370: .... 'RNA was then used to detect 6mA using UHPLC-QQQ after RNase A/T1 overnight digestion and ultrafiltration with MRM of A (268.1/152.1) and m6A (282.1/166.1)'. I guess ultrafiltration was done using some membrane/ column, and not with MRM. Please rephrase the sentence it is confusing.
9. Line 647 20 µ should be 20 µm

Referee #1 (Report for Author)

While I appreciate that the authors have made efforts to revise the ms, it unfortunately still requires major revision. The conclusions/assumptions the authors make are not fully supported by the data presented, and for some reviewer's comments the authors failed to provide satisfactory answers.

Major points
1. Authors failed to provide satisfactory answers to reviewer's comments:
a. Response to reviewer 1 comment 1: The experiments that confirm no ionization suppression were the ones described in lines 266-269, however the amounts of dA used in this experiment is 4 pmol s= 4x10-12 moles ,which is more than 100 times lower than the ones used in gDNA samples. Assuming average Mw of nucleotide in DNA is 310 g/mol, and dA represents~ 25 % of DNA sequence content, one can estimate that 2 µg of DNA roughly contains ~ 1.6 x10-9 moles or 1.6 nmoles of dA (2×10-6(g)/310 (g/mol)/4).
Additionally I disagree with an argument that dA isotopes are too difficult/expensive, a variety of dA with a different combination of isotope atoms are commercially available from several companies. (e.g https://www.silantes-shop.com). The commercial dA nucleoside isotopic standard can be added after DNA digest, to all samples including standards.
Lines 268 says that 4 pmol dA is spiked in into 'biological samples', but what are these biological samples is not clear. If these are the 2 µg of gDNA then 4 pmols of dA spike in will be 100x lower then dA present in gDNA samples, which makes no sense.

We are happy to perform ionization suppression control experiments at 100x higher concentration. If this is deemed insufficient, we can also redo the 16- and 64-cell stages with all isotopically-labeled controls as per the reviewer's recommendation. The latter would require additional two-three months of work because the amount of DNA required corresponds to several 100,000s embryos per stage.

b. Response to reviewer 1 comment 2 : In lines 76-77, authors observe that a measurement of an identical control sample (Fig 1 B and C 'CTRL(-)') with QQQ instrument returns values 10 x greater then Q-Trap, and authors still conclude that Q-trap results confirm that QQQ measurements are all correct. An identical control sample must contain identical levels of m6A, thus if both methods work, the measured values must be similar. Author's arguments that this difference can be explained by difference in sensitivity is wrong. Poor sensitivity will just not be able to detect any signal, and the values will be missing.
The only conclusion from the observed results, which can be made, is that QQQ measurements are erroneous and thus misleading, and should be optimized to either solve this inconsistency or be replaced with q-trap experiments.

The QQQ experiments were performed a few years ago as an initial broad screen. At that time, it was the most easy and economical way, despite its relative inaccuracy. It has been known that QQQ pipelines can result in a low level of false positive detection originating from the bacterial enzymes used for digestion (Greer et al. 2015). The level of 6mA 'background' contamination from the enzymes would be different from batch to batch, as understood in the community (Schiffers et al. 2017, O'Brown et al. 2019). Indeed, we were using different batches of digestive enzymes in these two different pipelines (QQQ vs QTRAP). Nevertheless, the QQQ screen revealed a high 6mA peak at the 16-cell stage and a background level concentration at the 64-cell stage and all other stages. It was confirmed by PacBio SMRT-seq sequencing, by immunofluorescence, and using QTRAP on the 16- and 64-cell stages only as there was little incentive to do the other stages in which the 6mA level was low (based on QQQ, sequencing, and immunofluorescence). As mentioned in our

response to the previous point, we can perform the QTRAP assays again using all controls, or just do the ionization suppression control. We could transfer the QQQ data to the supplement or removed altogether as they do not make any difference to the paper's main points.

c. Response to Reviewer 2 minor comment 1: states that labeling of 6mA and EU is impossible for identical samples due to incompatibility of the techniques. However, such staining can be performed in parallel on two identical samples. Alternatively, authors could provide LC/MS based quantifications of 6mA instead in analogous samples.

Parallel staining of EU and 6mA, and LC/MS quantification in analogous samples, is already provided in Figures 2A and 2C, respectively.

2. Authors should not assign the processes to a precise stage, since experiments performed only at selected stages with intermediate stages being omitted.
a. Lines 125-126: using results of fig 3B where two stages are shown, '16-cell' and '64-cell', authors conclude that transcriptional wave is starting at 64-cell stage. Stage '32- cell' is not shown, thus authors must consider that the wave may start at '32-cell' stage as well.

We do have 32-cell stage figures showing only minimal EU incorporation. We will include these in a new supplemental figure. We had originally left them out because they did not provide any significant information.

b. Lines 136-138: discusses that EU incorporation was delayed upon shAlkbh1, but they only show stage 512-cell and 64-cell. It would be beneficial to also include intermediate stages 128- and 256-cell and ideally even stages 16- and 32-cell. Additionally the two panels from Fig 2-A and EV2-A must be shown in one. Considering the conclusion authors made, that should be limited to only editorial edits.

We do have data from 32- and 128-cell stages (both showing only minimal incorporation) and can do 256 as well.

3. Authors conclusions are not fully supported with the data provided:
a. Line 139 says that results from EU incorporation suggest that 6mA levels follow EU patterns (linked to zygotic genome activation). This is just a hypothesis, moreover, Fig EV2C is confusing, the bars are only hypothetical. The effects of Alkbh1 on 6mA levels must be confirmed experimentally using LC/MS.

We show that upon Alkbh1 knockdown, 6mA level is elevated, confirmed by LC/MS (Figure 2C). Parallel knockdown experiment abolished EU incorporation at the same embryonic stage (Figure 4). Therefore, there is a temporal correlation between 6mA level and zygotic genome activation. This is shown functional by Alkbh1 knockdown. Figure EV2C is an expected scenario as indicated in the heading. It is does not discuss Alkbh1.

b. Lines 152-163: Authors performed the knock down of N6amt1 and Mettl4 and observe no zygotic genome activation by EU incorporation (fig EV2D), then they conclude that these enzymes do not deposit 6mA.

First, stage is not indicated; Second, for the shMettl4 sample, EU signal is present, while for the shN6AMT1 sample the EU signal is absent. The effects of each protein knockdowns are

not identical on EU level, how authors arrive at the conclusion above is mysterious.
Authors should show EU labeling for different stages to clearly demonstrate when EU signal is absent and when it is presen. Also a stage at which ZGA starts in each treatment will be clearly identifiable. Something like from 16-cell stage - to a stage when all samples show EU signal. Moreover, authors must include shGFP or another non-targeting control.
To make any conclusion about the effect of the two above mentioned proteins on 6mA levels in DNA, authors must provide 6mA LC/MS measurements for all stages, which will be shown for EU labeling.

The reviewer had already raised this point in the first review round. Unfortunately, despite intending to tone-down the statement, we have eventually failed to do so. We will rephrase the statement to reflect the actual data that we have.

c. Line 170-172 authors measure m6A levels in maternally provided RNA in the embryos; observing a slight decrease in m6A levels authors conclude that RNA is degraded between stages 2-cell and 16-cell, (fig5A), it is an overstatement. For instance, methyl groups from m6A could be removed RNA by Alkbh5 or Alkbh1 on otherwise intact RNA.

We will rephrase the statement.

d. Fig. EV3E: Knock down of Adal shows decrease of EU incorporation at 64-cell stage. Again controls showing proper development are missing, as well as stages before and after 64 cell stage, till the stage where EU signal appears in all samples. Apart from that, 6mA levels in DNA need to be determined by LC/MS to confirm that Adal knock down indeed causes 6mA levels increase in DNA.

In the first revision cycle, we were requested by the reviewer to perform Adal1 knockdown and monitor a possible delay of zygotic transcription, which we have done. Providing a full analysis of Adal1 is, arguably, outside the scope of this study. The list of genes that potentially contribute to 6mA clearance is not exhausted. Adding the full analysis of Adal1 would only add incremental knowledge and would not change the main conclusion. It would, however, require additional six months of work. The Adal1 results are only briefly mentioned in the text. We suggest leaving it as such.

4. The model shown on fig 4C is confusing, what does Alkbh1 do at stage 16? Why at stage 16 m6A is present and at stage 64 is almost absent except the two sites bound (protected?) by Alkbh1?
Model shown on fig 5d is also confusing and why Alkbh1 is absent here at stage 16, what is the difference between stage 16 on the models from fig 4C and 5D?
Moreover, authors should only present single model and show all they want clearly on that drawing, and explain all that explicitly in figure legend.
Model in fig 5D states that RNR is activated at 8-cell stage, how do authors know this? HU experiments that indicate that HU stops development at stage 8 is not related to the time when HU is activated. It just shows that dNTP pools reach critical levels.

We will provide a single, simpler model and rephrase the statement on HU.

Minor points:
1. 'block-i1 solution' terminology appears in line 287 but composition :(3% BSA and 0.25% Triton-X in PBS) is listed much later in line 314. Composition must be described at the first instance it appears in text.

This will be done.

2. Figures in general : Embryonic stages are sometimes labeled as 'c' or 'cell' on different figures, authors should be consistent

This will be done.

3. Fig 4C authors must indicate that each spot corresponds to single nucleus and state how many different embryos/slides were used, that information must be included directly in the figure legend. Fig 5A: Y-axis says m6A/A in arb units, why then the % is used, it is confusing since it assumes % of m6A over A, which is not the case for that measurement thus usage of % in this case should be avoided.

This will be done.

4. Fig EV1A: 'variable standard' term is confusing, call it for instance 'expected '

This will be done.

5. Fig EV2C: All of the bars must be labeled not only 16c.

This will be done.

6. Line 140: Albh1 should be Alkbh1

This will be done.

7. Line 249: 'chilled in ice' should be 'chilled on ice'

This will be done.

8. Lines 369-370: .... 'RNA was then used to detect 6mA using UHPLC-QQQ after RNase A/T1 overnight digestion and ultrafiltration with MRM of A (268.1/152.1) and m6A (282.1/166.1)'. I guess ultrafiltration was done using some membrane/ column, and not with MRM. Please rephrase the sentence it is confusing.

This will be done.

9. Line 647 20 μ should be 20 μm

This will be done.

Dear Uri,

Thank you for providing a preliminary revision plan for your manuscript. I sincerely apologise for the delay due to the Easter holidays. I have now received a positive assessment of your revision proposal from reviewer #1, who has also adjusted the experimental requests in response to your comments, which are copied below. I would therefore like to invite you to submit a revised manuscript according to the agreed-upon revision plan.

We generally allow three months as standard revision time. As a matter of policy, competing manuscripts published during this period will not negatively impact on our assessment of the conceptual advance presented by your study. However, please contact me as soon as possible upon publication of any related work to discuss the appropriate course of action. Should you foresee a problem in meeting this three-month deadline, please let us know in advance in order to arrange an extension.

When preparing your letter of response to the referees' comments, please bear in mind that this will form part of the Review Process File and will therefore be available online to the community. For more details on our Transparent Editorial Process, please visit our website: https://www.embopress.org/page/journal/14602075/authorguide#transparentprocess.

Please feel free to contact me if have any further questions regarding the revision. Thank you for the opportunity to consider your work for publication. I look forward to receiving your revision.

With best regards,

Ieva

Ieva Gailite, PhD
Senior Scientific Editor
The EMBO Journal
Meyerhofstrasse 1
D-69117 Heidelberg
Tel: +4962218891309
i.gailite@embojournal.org

Instructions for preparing your revised manuscript:

Please make sure you upload a letter of response to the referees' comments together with the revised manuscript.

Please also check that the title and abstract of the manuscript are brief, yet explicit, even to non-specialists.

When assembling figures, please refer to our figure preparation guideline in order to ensure proper formatting and readability in print as well as on screen:
https://bit.ly/EMBOPressFigurePreparationGuideline
See also figure legend guidelines: https://www.embopress.org/page/journal/14602075/authorguide#figureformat

At EMBO Press we ask authors to provide source data for the main manuscript figures. Our source data coordinator will contact you to discuss which figure panels we would need source data for and will also provide you with helpful tips on how to upload and organize the files.

IMPORTANT: When you send the revision we will require
- a point-by-point response to the referees' comments, with a detailed description of the changes made (as a word file).
- a word file of the manuscript text.
- individual production quality figure files (one file per figure)
- a complete author checklist, which you can download from our author guidelines (https://www.embopress.org/page/journal/14602075/authorguide).
- Expanded View files (replacing Supplementary Information)
Please see out instructions to authors
https://www.embopress.org/page/journal/14602075/authorguide#expandedview

Please remember: Digital image enhancement is acceptable practice, as long as it accurately represents the original data and conforms to community standards. If a figure has been subjected to significant electronic manipulation, this must be noted in the

figure legend or in the 'Materials and Methods' section. The editors reserve the right to request original versions of figures and the original images that were used to assemble the figure.

Further information is available in our Guide For Authors: https://www.embopress.org/page/journal/14602075/authorguide

We realize that it is difficult to revise to a specific deadline. In the interest of protecting the conceptual advance provided by the work, we recommend a revision within 3 months (16th Jul 2023). Please discuss the revision progress ahead of this time with the editor if you require more time to complete the revisions. Use the link below to submit your revision:

Link Not Available

Referee 1:

In general I appreciate this study and most results do support the main message that 6mA increases at stage 16 in gDNA and 6mA levels correlate well with the timing of zygotic genome activation. But there remain issues to be resolved, not least in the way data are presented and discussed.

Referee #1 (Report for Author)

While I appreciate that the authors have made efforts to revise the ms, it unfortunately still requires major revision. The conclusions/assumptions the authors make are not fully supported by the data presented, and for some reviewer's comments the authors failed to provide satisfactory answers.

Major points
1. Authors failed to provide satisfactory answers to reviewer's comments:
a. Response to reviewer 1 comment 1: The experiments that confirm no ionization suppression were the ones described in lines 266-269, however the amounts of dA used in this experiment is 4 pmol s= 4x10-12 moles ,which is more than 100 times lower than the ones used in gDNA samples. Assuming average Mw of nucleotide in DNA is 310 g/mol, and dA represents~ 25 % of DNA sequence content, one can estimate that 2 µg of DNA roughly contains ~ 1.6 x10-9 moles or 1.6 nmoles of dA (2×10-6(g)/310 (g/mol)/4).
Additionally I disagree with an argument that dA isotopes are too difficult/expensive, a variety of dA with a different combination of isotope atoms are commercially available from several companies. (e.g https://www.silantes-shop.com). The commercial dA nucleoside isotopic standard can be added after DNA digest, to all samples including standards.
Lines 268 says that 4 pmol dA is spiked in into 'biological samples', but what are these biological samples is not clear. If these are the 2 µg of gDNA then 4 pmols of dA spike in will be 100x lower then dA present in gDNA samples, which makes no sense.

We are happy to perform ionization suppression control experiments at 100x higher concentration. If this is deemed insufficient, we can also redo the 16- and 64-cell stages with all isotopically-labeled controls as per the reviewer's recommendation. The latter would require additional two-three months of work because the amount of DNA required corresponds to several 100,000s embryos per stage.

Reviewer response:

First, I was not aware of the complexity of the experiments, and this is another example where the authors could provide more details. In the DNA isolation section the number of embryos in buffer volume these embryos were suspended in, ratio of embryos to phenol/chlorophorm are missing and the reference in lines 234-231 do not include where the DNA purification procedure for Hydractinia embryos is described.

Second, my major reason to ask for these technical controls was to understand why a "Ctrl (-)" sample on fig 1 B and C shows two different values, see my next comment. The explanation provided by the authors that two methods detect different levels of the signals in identical sample due to different instrumentations used appears wrong to me (lines 75-78 :This method enabled us to detect 6mA/A levels of 0.01% as being distinct from negative control.")
As I can now see from authors response to comment 2, I was correct, it was not the instrument as authors stated in the manuscript, but the sample itself.

Now knowing the reason of the observed discrepancy in the "Ctrl" sample, I have no problem with the difference in the measurements anymore. However, I think it is also important to deliver this information to the future readers as well.

As a compromise for this and the next point, I suggest authors do only editorial corrections, clearly state that the Ctrl samples

from two panels can not be directly compared and state in the manuscript the true reasons. Moreover, authors should clearly indicate, on the fig 1B, with a line (band including error bars) the 6mA measurements, which are not correct, since they are masked by high 6mA background level of the given experiment (experiment, not a method and not an instrument!). Thus, the true levels of 6mA of embryonic gDNA are most likely even lower and could not be detected.

b. Response to reviewer 1 comment 2 : In lines 76-77, authors observe that a measurement of an identical control sample (Fig 1 B and C 'CTRL(-)') with QQQ instrument returns values 10 x greater then Q-Trap, and authors still conclude that Q-trap results confirm that QQQ measurements are all correct. An identical control sample must contain identical levels of m6A, thus if both methods work, the measured values must be similar. Author's arguments that this difference can be explained by difference in sensitivity is wrong. Poor sensitivity will just not be able to detect any signal, and the values will be missing.
The only conclusion from the observed results, which can be made, is that QQQ measurements are erroneous and thus misleading, and should be optimized to either solve this inconsistency or be replaced with q-trap experiments.

The QQQ experiments were performed a few years ago as an initial broad screen. At that time, it was the most easy and economical way, despite its relative inaccuracy. It has been known that QQQ pipelines can result in a low level of false positive detection originating from the bacterial enzymes used for digestion (Greer et al. 2015). The level of 6mA 'background' contamination from the enzymes would be different from batch to batch, as understood in the community (Schiffers et al. 2017, O'Brown et al. 2019). Indeed, we were using different batches of digestive enzymes in these two different pipelines (QQQ vs QTRAP). Nevertheless, the QQQ screen revealed a high 6mA peak at the 16-cell stage and a background level concentration at the 64-cell stage and all other stages. It was confirmed by PacBio SMRT-seq sequencing, by immunofluorescence, and using QTRAP on the 16- and 64-cell stages only as there was little incentive to do the other stages in which the 6mA level was low (based on QQQ, sequencing, and immunofluorescence). As mentioned in our response to the previous point, we can perform the QTRAP assays again using all controls, or just do the ionization suppression control. We could transfer the QQQ data to the supplement or removed altogether as they do not make any difference to the paper's main points.

See the reviewer response to first point.

c. Response to Reviewer 2 minor comment 1: states that labelling of 6mA and EU is impossible for identical samples due to incompatibility of the techniques. However, such staining can be performed in parallel on two identical samples. Alternatively, authors could provide LC/MS based quantifications of 6mA instead in analogous samples.

Parallel staining of EU and 6mA, and LC/MS quantification in analogous samples, is already provided in Figures 2A and 2C, respectively.

Reviewer response:

I agree, the fig 2C data I overlooked

2. Authors should not assign the processes to a precise stage, since experiments performed only at selected stages with intermediate stages being omitted.
a. Lines 125-126: using results of fig 3B where two stages are shown, '16-cell' and '64-cell', authors conclude that transcriptional wave is starting at 64-cell stage. Stage '32- cell' is not shown, thus authors must consider that the wave may start at '32-cell' stage as well.

We do have 32-cell stage figures showing only minimal EU incorporation. We will include these in a new supplemental figure. We had originally left them out because they did not provide any significant information.

Reviewer response:

Please do so.

b. Lines 136-138: discusses that EU incorporation was delayed upon shAlkbh1, but they only show stage 512-cell and 64-cell. It

would be beneficial to also include intermediate stages 128- and 256-cell and ideally even stages 16- and 32-cell. Additionally the two panels from Fig 2-A and EV2-A must be shown in one. Considering the conclusion authors made, that should be limited to only editorial edits.

We do have data from 32- and 128-cell stages (both showing only minimal incorporation) and can do 256 as well.

Reviewer response:

Please do so.

3. Authors conclusions are not fully supported with the data provided:
a. Line 139 says that results from EU incorporation suggest that 6mA levels follow EU patterns (linked to zygotic genome activation). This is just a hypothesis, moreover, Fig EV2C is confusing, the bars are only hypothetical. The effects of Alkbh1 on 6mA levels must be confirmed experimentally using LC/MS.

We show that upon Alkbh1 knockdown, 6mA level is elevated, confirmed by LC/MS (Figure 2C). Parallel knockdown experiment abolished EU incorporation at the same embryonic stage (Figure 4). Therefore, there is a temporal correlation between 6mA level and zygotic genome activation. This is shown functional by Alkbh1 knockdown. Figure EV2C is an expected scenario as indicated in the heading. It is does not discuss Alkbh1.

Reviewer response:

Considering, I overlooked the MS measurements presented on fig 2C, I agree that EU measurements and 6mA levels correlate the data is there, and the conclusion can be made.

b. Lines 152-163: Authors performed the knock down of N6amt1 and Mettl4 and observe no zygotic genome activation by EU incorporation (fig EV2D), then they conclude that these enzymes do not deposit 6mA.

First, stage is not indicated; Second, for the shMettl4 sample, EU signal is present, while for the shN6AMT1 sample the EU signal is absent. The effects of each protein knockdowns are not identical on EU level, how authors arrive at the conclusion above is mysterious.
Authors should show EU labeling for different stages to clearly demonstrate when EU signal is absent and when it is presen. Also a stage at which ZGA starts in each treatment will be clearly identifiable. Something like from 16-cell stage - to a stage when all samples show EU signal. Moreover, authors must include shGFP or another non-targeting control.
To make any conclusion about the effect of the two above mentioned proteins on 6mA levels in DNA, authors must provide 6mA LC/MS measurements for all stages, which will be shown for EU labeling.

The reviewer had already raised this point in the first review round. Unfortunately, despite intending to tone-down the statement, we have eventually failed to do so. We will rephrase the statement to reflect the actual data that we have.

Reviewer response:

Please do so.

c. Line 170-172 authors measure m6A levels in maternally provided RNA in the embryos; observing a slight decrease in m6A levels authors conclude that RNA is degraded between stages 2-cell and 16-cell, (fig5A), it is an overstatement. For instance, methyl groups from m6A could be removed RNA by Alkbh5 or Alkbh1 on otherwise intact RNA.

We will rephrase the statement.

Reviewer response:

Please do so.

d. Fig. EV3E: Knock down of Adal shows decrease of EU incorporation at 64-cell stage. Again controls showing proper development are missing, as well as stages before and after 64 cell stage, till the stage where EU signal appears in all samples. Apart from that, 6mA levels in DNA need to be determined by LC/MS to confirm that Adal knock down indeed causes 6mA levels increase in DNA.

In the first revision cycle, we were requested by the reviewer to perform Adal1 knockdown and monitor a possible delay of zygotic transcription, which we have done. Providing a full analysis of Adal1 is, arguably, outside the scope of this study. The list of genes that potentially contribute to 6mA clearance is not exhausted. Adding the full analysis of Adal1 would only add incremental knowledge and would not change the main conclusion. It would, however, require additional six months of work. The Adal1 results are only briefly mentioned in the text. We suggest leaving it as such.

Reviewer response:

I think that Adal experiments provide additional evidence and strengthen the main hypothesis.

Also, I did not ask authors to identify which enzymes are involved in nucleotide demethylation. Instead, to use one of the potential major effectors as a tool to manipulate m6A/6mA nucleotide pools and subsequent 6mA introduction into the gDNA. ALKBH1, an analogous experiment, may affect both: m6A in RNA ,and 6mA in DNA, by direct demethylation of the methylated base within RNA or DNA. Adal enzymes, at least in mammals, only work on nucleotide level, thus a scenario when 6mA is erased from DNA directly can be excluded.

Additionally, manipulations with a protein from a different pathway minimise the possibility of indirect effects Alkbh1 manipulations may have on 6mA and zygotic genome activation.

I understand that redoing the experiment and including all the measurements is difficult, thus, requested m6A MS measurements indeed may be excluded. However I think providing EU staining for later stages (at least two within short developmental time between each other, where earlier stage shows no or weak EU signal and later stage stronger EU signals and respective Mock controls) can be performed.

4. The model shown on fig 4C is confusing, what does Alkbh1 do at stage 16? Why at stage 16 m6A is present and at stage 64 is almost absent except the two sites bound (protected?) by Alkbh1?

Model shown on fig 5d is also confusing and why Alkbh1 is absent here at stage 16, what is the difference between stage 16 on the models from fig 4C and 5D?
Moreover, authors should only present single model and show all they want clearly on that drawing, and explain all that explicitly in figure legend.
Model in fig 5D states that RNR is activated at 8-cell stage, how do authors know this? HU experiments that indicate that HU stops development at stage 8 is not related to the time when HU is activated. It just shows that dNTP pools reach critical levels.

We will provide a single, simpler model and rephrase the statement on HU.

Reviewer response:

Please do so.

***
Rev_Com_number: RC-2022-01616
New_manu_number: EMBOJ-2022-112934R
Corr_author: Frank
Title: Randomly incorporated genomic 6mA delays zygotic transcription initiation in Hydractinia

Referee 1:

In general I appreciate this study and most results do support the main message that 6mA increases at stage 16 in gDNA and 6mA levels correlate well with the timing of zygotic genome activation. But there remain issues to be resolved, not least in the way data are presented and discussed.

Referee #1 (Report for Author)

While I appreciate that the authors have made efforts to revise the ms, it unfortunately still requires major revision. The conclusions/assumptions the authors make are not fully supported by the data presented, and for some reviewer's comments the authors failed to provide satisfactory answers.

Major points
1. Authors failed to provide satisfactory answers to reviewer's comments:
a. Response to reviewer 1 comment 1: The experiments that confirm no ionization suppression were the ones described in lines 266-269, however the amounts of dA used in this experiment is 4 pmol s= 4x10-12 moles ,which is more than 100 times lower than the ones used in gDNA samples. Assuming average Mw of nucleotide in DNA is 310 g/mol, and dA represents~ 25 % of DNA sequence content, one can estimate that 2 µg of DNA roughly contains ~ 1.6 x10-9 moles or 1.6 nmoles of dA (2×10-6(g)/310 (g/mol)/4).
Additionally I disagree with an argument that dA isotopes are too difficult/expensive, a variety of dA with a different combination of isotope atoms are commercially available from several companies. (e.g https://www.silantes-shop.com). The commercial dA nucleoside isotopic standard can be added after DNA digest, to all samples including standards.
Lines 268 says that 4 pmol dA is spiked in into 'biological samples', but what are these biological samples is not clear. If these are the 2 µg of gDNA then 4 pmols of dA spike in will be 100x lower then dA present in gDNA samples, which makes no sense.

We are happy to perform ionization suppression control experiments at 100x higher concentration. If this is deemed insufficient, we can also redo the 16- and 64-cell stages with all isotopically-labeled controls as per the reviewer's recommendation. The latter would require additional two-three months of work because the amount of DNA required corresponds to several 100,000s embryos per stage.

Reviewer response:

First, I was not aware of the complexity of the experiments, and this is another example where the authors could provide more details. In the DNA isolation section the number of embryos in buffer volume these embryos were suspended in, ratio of embryos to phenol/chlorophorm are missing and the reference in lines 234-231 do not include where the DNA purification procedure for Hydractinia embryos is described.

We have provided more details on this section describing the protocols of DNA extraction.

Second, my major reason to ask for these technical controls was to understand why a “Ctrl (-)” sample on fig 1 B and C shows two different values, see my next comment. The explanation provided by the authors that two methods detect different levels of the signals in identical sample due to different instrumentations used appears wrong to me (lines 75-78 :This method enabled us to detect 6mA/A levels of 0.01% as being distinct from negative control.”)
As I can now see from authors response to comment 2, I was correct, it was not the

instrument as authors stated in the manuscript, but the sample itself.

We have changed the manuscript accordingly (see manuscript with tracked changes).

Now knowing the reason of the observed discrepancy in the "Ctrl" sample, I have no problem with the difference in the measurements anymore. However, I think it is also important to deliver this information to the future readers as well.

As a compromise for this and the next point, I suggest authors do only editorial corrections, clearly state that the Ctrl samples from two panels cannot be directly compared and state in the manuscript the true reasons. Moreover, authors should clearly indicate, on the fig 1B, with a line (band including error bars) the 6mA measurements, which are not correct, since they are masked by high 6mA background level of the given experiment (experiment, not a method and not an instrument!). Thus, the true levels of 6mA of embryonic gDNA are most likely even lower and could not be detected.

We have modified Fig 1B and C, and pointed out in the text the differences in the experiments and samples between the two protocols.

b. Response to reviewer 1 comment 2 : In lines 76-77, authors observe that a measurement of an identical control sample (Fig 1 B and C 'CTRL(-)') with QQQ instrument returns values 10 x greater then Q-Trap, and authors still conclude that Q-trap results confirm that QQQ measurements are all correct. An identical control sample must contain identical levels of m6A, thus if both methods work, the measured values must be similar. Author's arguments that this difference can be explained by difference in sensitivity is wrong. Poor sensitivity will just not be able to detect any signal, and the values will be missing.
The only conclusion from the observed results, which can be made, is that QQQ measurements are erroneous and thus misleading, and should be optimized to either solve this inconsistency or be replaced with q-trap experiments.

The QQQ experiments were performed a few years ago as an initial broad screen. At that time, it was the most easy and economical way, despite its relative inaccuracy. It has been known that QQQ pipelines can result in a low level of false positive detection originating from the bacterial enzymes used for digestion (Greer et al. 2015). The level of 6mA 'background' contamination from the enzymes would be different from batch to batch, as understood in the community (Schiffers et al. 2017, O'Brown et al. 2019). Indeed, we were using different batches of digestive enzymes in these two different pipelines (QQQ vs QTRAP). Nevertheless, the QQQ screen revealed a high 6mA peak at the 16-cell stage and a background level concentration at the 64-cell stage and all other stages. It was confirmed by PacBio SMRT-seq sequencing, by immunofluorescence, and using QTRAP on the 16- and 64-cell stages only as there was little incentive to do the other stages in which the 6mA level was low (based on QQQ, sequencing, and immunofluorescence). As mentioned in our response to the previous point, we can perform the QTRAP assays again using all controls, or just do the ionization suppression control. We could transfer the QQQ data to the supplement or removed altogether as they do not make any difference to the paper's main points.

See the reviewer response to first point.

See our response to first point

c. Response to Reviewer 2 minor comment 1: states that labelling of 6mA and EU is

impossible for identical samples due to incompatibility of the techniques. However, such staining can be performed in parallel on two identical samples. Alternatively, authors could provide LC/MS based quantifications of 6mA instead in analogous samples.

Parallel staining of EU and 6mA, and LC/MS quantification in analogous samples, is already provided in Figures 2A and 2C, respectively.

Reviewer response:

I agree, the fig 2C data I overlooked

Thank you

2. Authors should not assign the processes to a precise stage, since experiments performed only at selected stages with intermediate stages being omitted.
a. Lines 125-126: using results of fig 3B where two stages are shown, '16-cell' and '64-cell', authors conclude that transcriptional wave is starting at 64-cell stage. Stage '32- cell' is not shown, thus authors must consider that the wave may start at '32-cell' stage as well.

We do have 32-cell stage figures showing only minimal EU incorporation. We will include these in a new supplemental figure. We had originally left them out because they did not provide any significant information.

Reviewer response:

Please do so.

Figures have been added (Figure 3B).

b. Lines 136-138: discusses that EU incorporation was delayed upon shAlkbh1, but they only show stage 512-cell and 64-cell. It would be beneficial to also include intermediate stages 128- and 256-cell and ideally even stages 16- and 32-cell. Additionally the two panels from Fig 2-A and EV2-A must be shown in one. Considering the conclusion authors made, that should be limited to only editorial edits.

We do have data from 32- and 128-cell stages (both showing only minimal incorporation) and can do 256 as well.

Reviewer response:

Please do so.

Additional experiments of shRNA *Alkbh1* injection and EU staining were performed. The data are presented in Figure EV2.

3. Authors conclusions are not fully supported with the data provided:

a. Line 139 says that results from EU incorporation suggest that 6mA levels follow EU patterns (linked to zygotic genome activation). This is just a hypothesis, moreover, Fig EV2C is confusing, the bars are only hypothetical. The effects of Alkbh1 on 6mA levels must be confirmed experimentally using LC/MS.

We show that upon Alkbh1 knockdown, 6mA level is elevated, confirmed by LC/MS (Figure 2C). Parallel knockdown experiment abolished EU incorporation at the same embryonic stage (Figure 4). Therefore, there is a temporal correlation between 6mA level and zygotic genome activation. This is shown functional by Alkbh1 knockdown. Figure EV2C is an expected scenario as indicated in the heading. It is does not discuss Alkbh1.

Reviewer response:

Considering, I overlooked the MS measurements presented on fig 2C, I agree that EU measurements and 6mA levels correlate the data is there, and the conclusion can be made.

Thank you

b. Lines 152-163: Authors performed the knock down of N6amt1 and Mettl4 and observe no zygotic genome activation by EU incorporation (fig EV2D), then they conclude that these enzymes do not deposit 6mA.

First, stage is not indicated; Second, for the shMettl4 sample, EU signal is present, while for the shN6AMT1 sample the EU signal is absent. The effects of each protein knockdowns are not identical on EU level, how authors arrive at the conclusion above is mysterious.
Authors should show EU labeling for different stages to clearly demonstrate when EU signal is absent and when it is presen. Also a stage at which ZGA starts in each treatment will be clearly identifiable. Something like from 16-cell stage - to a stage when all samples show EU signal. Moreover, authors must include shGFP or another non-targeting control.
To make any conclusion about the effect of the two above mentioned proteins on 6mA levels in DNA, authors must provide 6mA LC/MS measurements for all stages, which will be shown for EU labeling.

The reviewer had already raised this point in the first review round. Unfortunately, despite intending to tone-down the statement, we have eventually failed to do so. We will rephrase the statement to reflect the actual data that we have.

Reviewer response:

Please do so.

This was done.

c. Line 170-172 authors measure m6A levels in maternally provided RNA in the embryos; observing a slight decrease in m6A levels authors conclude that RNA is degraded between stages 2-cell and 16-cell, (fig5A), it is an overstatement. For instance, methyl groups from m6A could be removed RNA by Alkbh5 or Alkbh1 on otherwise intact RNA.

We will rephrase the statement.

Reviewer response:

Please do so.

This was done.

d. Fig. EV3E: Knock down of Adal shows decrease of EU incorporation at 64-cell stage. Again controls showing proper development are missing, as well as stages before and after 64 cell stage, till the stage where EU signal appears in all samples. Apart from that, 6mA levels in DNA need to be determined by LC/MS to confirm that Adal knock down indeed causes 6mA levels increase in DNA.

In the first revision cycle, we were requested by the reviewer to perform Adal1 knockdown and monitor a possible delay of zygotic transcription, which we have done. Providing a full analysis of Adal1 is, arguably, outside the scope of this study. The list of genes that potentially contribute to 6mA clearance is not exhausted. Adding the full analysis of Adal1 would only add incremental knowledge and would not change the main conclusion. It would, however, require additional six months of work. The Adal1 results are only briefly mentioned in the text. We suggest leaving it as such.

Reviewer response:

I think that Adal experiments provide additional evidence and strengthen the main hypothesis.

Also, I did not ask authors to identify which enzymes are involved in nucleotide demethylation. Instead, to use one of the potential major effectors as a tool to manipulate m6A/6mA nucleotide pools and subsequent 6mA introduction into the gDNA. ALKBH1, an analogous experiment, may affect both: m6A in RNA ,and 6mA in DNA, by direct demethylation of the methylated base within RNA or DNA. Adal enzymes, at least in mammals, only work on nucleotide level, thus a scenario when 6mA is erased from DNA directly can be excluded.

Additionally, manipulations with a protein from a different pathway minimise the possibility of indirect effects Alkbh1 manipulations may have on 6mA and zygotic genome activation.

I understand that redoing the experiment and including all the measurements is difficult, thus, requested m6A MS measurements indeed may be excluded. However I think providing EU staining for later stages (at least two within short developmental time between each other, where earlier stage shows no or weak EU signal and later stage stronger EU signals and respective Mock controls) can be performed.

We thank the reviewer for the suggestion to use the Adal1 experiments as evidence for RNA degradation. We have performed the additional requested experiments of shRNA to target *Adal1* and assessed EU incorporation at 128- and 256-cell embryos. The data are provided in Fig EV3E

4. The model shown on fig 4C is confusing, what does Alkbh1 do at stage 16? Why at stage 16 m6A is present and at stage 64 is almost absent except the two sites bound (protected?) by Alkbh1?

Model shown on fig 5d is also confusing and why Alkbh1 is absent here at stage 16, what is the difference between stage 16 on the models from fig 4C and 5D?
Moreover, authors should only present single model and show all they want clearly on that drawing, and explain all that explicitly in figure legend.
Model in fig 5D states that RNR is activated at 8-cell stage, how do authors know this? HU experiments that indicate that HU stops development at stage 8 is not related to the time when HU is activated. It just shows that dNTP pools reach critical levels.

We will provide a single, simpler model and rephrase the statement on HU.

Reviewer response:

Please do so.

We have removed the model from Figure 4C and placed a new model in Figure 5D. The text was modified accordingly.

***

Rev_Com_number: RC-2022-01616
New_manu_number: EMBOJ-2022-112934R
Corr_author: Frank
Title: Randomly incorporated genomic 6mA delays zygotic transcription initiation in Hydractinia

2nd Revision - Editorial Decision 12th Jun 2023

Dear Uri,

Thank you for submitting your revised manuscript to The EMBO Journal. I sincerely apologise for the protracted handling process of your manuscript due to the high number of submissions we are experiencing at the moment. Your manuscript has now been seen by reviewer #1, who finds that their main concerns have been addressed and now recommends publication of the manuscript. I will therefore be happy to accept the manuscript for publication in The EMBO Journal after the remaining comments by the reviewer and the following minor editorial issues are addressed:

1. Our publisher has done their pre-publication check on your manuscript. I have attached the file here. Please take a look at the word file and the comments regarding the figure legends and respond to the issues.
2. Dataset EV2 currently consists of multiple sequence files that are zipped together. Please consider uploading this folder as source data for the appropriate figure.
3. The source data for Appendix Figures currently include figure panels rather than the original, unmodified images of blots or immunofluorescence images. Please either submit such files or remove the modified figure panels from this folder.

Please feel free to contact me if you have any questions regarding this final editorial revision. Please use the link below to upload the revised files.

Thank you for the opportunity to consider your work for publication, and I look forward to receiving your revised manuscript.

With best wishes,

Ieva

Ieva Gailite, PhD
Senior Scientific Editor
The EMBO Journal
Meyerhofstrasse 1
D-69117 Heidelberg
Tel: +4962218891309
i.gailite@embojournal.org

Further information is available in our Guide For Authors: https://www.embopress.org/page/journal/14602075/authorguide

Revision to The EMBO Journal should be submitted online within 90 days, unless an extension has been requested and approved by the editor; please click on the link below to submit the revision online before 10th Sep 2023:

Link Not Available

Referee #1:

The authors have satisfyingly revised the ms and it can be published. 2 minor points:

1. Fig EV2-A please label all the stages on the figure.
2. Fig EV2-D was it stage 16 cells? please indicate that in the legend.

***
Rev_Com_number: RC-2022-01616
New_manu_number: EMBOJ-2022-112934R1
Corr_author: Frank
Title: Randomly incorporated genomic 6mA delays zygotic transcription initiation in a cnidarian

The authors addressed all minor editorial requests.

3rd Revision - Editorial Decision 16th Jun 2023

Dear Uri,

Thank you for addressing the most of the final editorial points. I am now pleased to inform you that your manuscript has been accepted for publication.

Before we forward your manuscript to our publishers, there are a couple of issues that need your further input. In particular, source data for the Appendix Figure S1A still includes a composite image of spliced-together dot blots. Please either send us a full, unedited image(s) of the original membrane(s) If these images are not available, let me know and I will remove the current, spliced image from the source data folder for this figure.

I will also look into the synopsis text in the next couple of days and let you know if any edits to the journal style are be needed.

Please note that it is EMBO Journal policy for the transcript of the editorial process (containing referee reports and your response letter) to be published as an online supplement to each paper. If you do NOT want this, you will need to inform the Editorial Office via email immediately. More information is available here: https://emboj.embopress.org/about#Transparent_Process

Your manuscript will be processed for publication in the journal by EMBO Press. Manuscripts in the PDF and electronic editions of The EMBO Journal will be copy edited, and you will be provided with page proofs prior to publication. Please note that supplementary information is not included in the proofs.

Please note that you will be contacted by Wiley Author Services to complete licensing and payment information. The 'Page Charges Authorization Form' is available here: https://www.embopress.org/pb-assets/embo-site/tej_apc.pdf

EMBO Press participates in many Publish and Read agreements that allow authors to publish Open Access with reduced/no publication charges. Check your eligibility: https://authorservices.wiley.com/author-resources/Journal-Authors/open-access/affiliation-policies-payments/index.html

Should you be planning a Press Release on your article, please get in contact with embojournal@wiley.com as early as possible, in order to coordinate publication and release dates.

If you have any questions, please do not hesitate to call or email the Editorial Office. Thank you for this contribution to The EMBO Journal and congratulations on a successful publication!

Best regards,

Ieva

Ieva Gailite, PhD
Senior Scientific Editor
The EMBO Journal
Meyerhofstrasse 1
D-69117 Heidelberg
Tel: +4962218891309
i.gailite@embojournal.org

***
Rev_Com_number: RC-2022-01616
New_manu_number: EMBOJ-2022-112934R2
Corr_author: Frank
Title: Randomly incorporated genomic 6mA delays zygotic transcription initiation in a cnidarian

EMBO Press Author Checklist

Corresponding Author Name: Uri Frank	
Journal Submitted to: EMBO Journal	
Manuscript Number: EMBOJ-2022-112934	

USEFUL LINKS FOR COMPLETING THIS FORM
The EMBO Journal - Author Guidelines
EMBO Reports - Author Guidelines
Molecular Systems Biology - Author Guidelines
EMBO Molecular Medicine - Author Guidelines

Reporting Checklist for Life Science Articles (updated January
This checklist is adapted from Materials Design Analysis Reporting (MDAR) Checklist for Authors. MDAR establishes a minimum set of requirements in transparent reporting in the life sciences (see Statement of Task: 10.31222/osf.io/9sm4x). Please follow the journal's guidelines in preparing your
Please note that a copy of this checklist will be published alongside your article.

Abridged guidelines for figures
1. Data
The data shown in figures should satisfy the following conditions:
- the data were obtained and processed according to the field's best practice and are presented to reflect the results of the experiments in an accurate and unbiased manner.
- ideally, figure panels should include only measurements that are directly comparable to each other and obtained with the same assay.
- plots include clearly labeled error bars for independent experiments and sample sizes. Unless justified, error bars should not be shown for technical
- if n<5, the individual data points from each experiment should be plotted. Any statistical test employed should be justified.
- Source Data should be included to report the data underlying figures according to the guidelines set out in the authorship guidelines on Data

2. Captions
Each figure caption should contain the following information, for each panel where they are relevant:
- a specification of the experimental system investigated (eg cell line, species name).
- the assay(s) and method(s) used to carry out the reported observations and measurements.
- an explicit mention of the biological and chemical entity(ies) that are being measured.
- an explicit mention of the biological and chemical entity(ies) that are altered/varied/perturbed in a controlled manner.
- the exact sample size (n) for each experimental group/condition, given as a number, not a range;
- a description of the sample collection allowing the reader to understand whether the samples represent technical or biological replicates (including how many animals, litters, cultures, etc.).
- a statement of how many times the experiment shown was independently replicated in the laboratory.
- definitions of statistical methods and measures:
  - common tests, such as t-test (please specify whether paired vs. unpaired), simple χ2 tests, Wilcoxon and Mann-Whitney tests, can be unambiguously identified by name only, but more complex techniques should be described in the methods section;
  - are tests one-sided or two-sided?
  - are there adjustments for multiple comparisons?
  - exact statistical test results, e.g., P values = x but not P values < x;
  - definition of 'center values' as median or average;
  - definition of error bars as s.d. or s.e.m.

Please complete ALL of the questions below.
Select "Not Applicable" only when the requested information is not relevant for your study.

Materials

Newly Created Materials	Information included in the manuscript?	In which section is the information available? (Reagents and Tools Table, Materials and Methods, Figures, Data Availability Section)
New materials and reagents need to be available; do any restrictions apply?	Yes	Materials and Methods

Antibodies	Information included in the manuscript?	In which section is the information available? (Reagents and Tools Table, Materials and Methods, Figures, Data Availability Section)
For **antibodies** provide the following information:<br>- Commercial antibodies: RRID (if possible) or supplier name, catalogue number and or/clone number<br>- Non-commercial: RRID or citation	Yes	Materials and Methods

DNA and RNA sequences	Information included in the manuscript?	In which section is the information available? (Reagents and Tools Table, Materials and Methods, Figures, Data Availability Section)
**Short novel DNA or RNA including primers, probes**: provide the sequences.	Yes	Appendix Table S1 and Data Availability

Cell materials	Information included in the manuscript?	In which section is the information available? (Reagents and Tools Table, Materials and Methods, Figures, Data Availability Section)
**Cell lines:** Provide species information, strain. Provide accession number in repository **OR** supplier name, catalog number, clone number, and/**OR** RRID.	Not Applicable	
**Primary cultures:** Provide species, strain, sex of origin, genetic modification status.	Not Applicable	
Report if the cell lines were recently **authenticated** (e.g., by STR profiling) and tested for mycoplasma contamination.	Not Applicable	

Experimental animals	Information included in the manuscript?	In which section is the information available? (Reagents and Tools Table, Materials and Methods, Figures, Data Availability Section)
**Laboratory animals or Model organisms:** Provide species, strain, sex, age, genetic modification status. Provide accession number in repository **OR** supplier name, catalog number, clone number, **OR** RRID.	Yes	Materials and Methods
**Animal observed in or captured from the field:** Provide species, sex, and age where possible.	Not Applicable	
Please detail **housing and husbandry conditions**.	Yes	Materials and Methods

Plants and microbes	Information included in the manuscript?	In which section is the information available? (Reagents and Tools Table, Materials and Methods, Figures, Data Availability Section)
**Plants:** provide species and strain, ecotype and cultivar where relevant, unique accession number if available, and source (including location for collected wild specimens).	Not Applicable	
**Microbes:** provide species and strain, unique accession number if available, and source.	Not Applicable	

Human research participants	Information included in the manuscript?	In which section is the information available? (Reagents and Tools Table, Materials and Methods, Figures, Data Availability Section)
If collected and within the bounds of privacy constraints report on age, sex and gender or ethnicity for all study participants.	Not Applicable	

Core facilities	Information included in the manuscript?	In which section is the information available? (Reagents and Tools Table, Materials and Methods, Figures, Data Availability Section)
If your work benefited from core facilities, was their service mentioned in the acknowledgments section?	Yes	Acknowledgments

Design

Study protocol	Information included in the manuscript?	In which section is the information available? (Reagents and Tools Table, Materials and Methods, Figures, Data Availability Section)

If study protocol has been **pre-registered, provide DOI in the manuscript**. For clinical trials, provide the trial registration number **OR** cite DOI.	Not Applicable	
Report the **clinical trial registration number** (at ClinicalTrials.gov or equivalent), where applicable.	Not Applicable	

Laboratory protocol	Information included in the manuscript?	In which section is the information available? (Reagents and Tools Table, Materials and Methods, Figures, Data Availability Section)
Provide DOI OR other citation details if **external detailed step-by-step protocols** are available.	Not Applicable	

Experimental study design and statistics	Information included in the manuscript?	In which section is the information available? (Reagents and Tools Table, Materials and Methods, Figures, Data Availability Section)
Include a statement about **sample size** estimate even if no statistical methods were used.	Yes	Figures and Materials and Methods
Were any steps taken to minimize the effects of subjective bias when allocating animals/samples to treatment (e.g. **randomization procedure**)? If yes, have they been described?	Not Applicable	
Include a statement about **blinding** even if no blinding was done.	Not Applicable	All Hydractinia embryos look similar to the naked eye
Describe **inclusion/exclusion criteria** if samples or animals were excluded from the analysis. Were the criteria pre-established?<br><br>If sample or data points were omitted from analysis, report if this was due to attrition or intentional exclusion and provide justification.	Not Applicable	
For every figure, are **statistical tests** justified as appropriate? Do the data meet the assumptions of the tests (e.g., normal distribution)? Describe any methods used to assess it. Is there an estimate of variation within each group of data? Is the variance similar between the groups that are being statistically compared?	Yes	Source Data

Sample definition and in-laboratory replication	Information included in the manuscript?	In which section is the information available? (Reagents and Tools Table, Materials and Methods, Figures, Data Availability Section)
In the figure legends: state number of times the experiment was **replicated** in laboratory.	Yes	Figures, Materials and Methods, and Source Data
In the figure legends: define whether data describe **technical or biological replicates**.	Yes	In the Source Data

Ethics

Ethics	Information included in the manuscript?	In which section is the information available? (Reagents and Tools Table, Materials and Methods, Figures, Data Availability Section)
Studies involving **human participants**: State details of **authority granting ethics approval** (IRB or equivalent committee(s), provide reference number for approval.	Not Applicable	
Studies involving **human participants**: Include a statement confirming that **informed consent** was obtained from all subjects and that the experiments conformed to the principles set out in the WMA Declaration of Helsinki and the Department of Health and Human Services Belmont Report.	Not Applicable	
Studies involving **human participants:** For publication of **patient photos**, include a statement confirming that consent to publish was obtained.	Not Applicable	
Studies involving experimental **animals**: State details of **authority granting ethics approval** (IRB or equivalent committee(s), provide reference number for approval. Include a statement of compliance with ethical regulations.	Not Applicable	
Studies involving **specimen and field samples:** State if relevant **permits** obtained, provide details of authority approving study; if none were required, explain why.	Not Applicable	

Dual Use Research of Concern (DURC)	Information included in the manuscript?	In which section is the information available? (Reagents and Tools Table, Materials and Methods, Figures, Data Availability Section)
Could your study fall under dual use research restrictions? Please check biosecurity documents and list of **select agents and toxins** (CDC): https://www.selectagents.gov/sat/list.htm	Not Applicable	
If you used a select agent, is the security level of the lab appropriate and reported in the manuscript?	Not Applicable	
If a study is subject to dual use research of concern regulations, is the name of the **authority granting approval and reference number** for the regulatory approval provided in the manuscript?	Not Applicable	

Reporting

The MDAR framework recommends adoption of discipline-specific guidelines, established and endorsed through community initiatives. Journals have their own policy about requiring specific guidelines and recommendations to complement MDAR.

Adherence to community standards	Information included in the manuscript?	In which section is the information available? (Reagents and Tools Table, Materials and Methods, Figures, Data Availability Section)
State if relevant guidelines or checklists (e.g., **ICMJE, MIBBI, ARRIVE, PRISMA**) have been followed or provided.	Not Applicable	
For **tumor marker prognostic studies,** we recommend that you follow the **REMARK** reporting guidelines (see link list at top right). See author guidelines, under 'Reporting Guidelines'. Please confirm you have followed these guidelines.	Not Applicable	
For **phase II and III randomized controlled trials**, please refer to the **CONSORT** flow diagram (see link list at top right) and submit the CONSORT checklist (see link list at top right) with your submission. See author guidelines, under 'Reporting Guidelines'. Please confirm you have submitted this list.	Not Applicable	

Data Availability

Data availability	Information included in the manuscript?	In which section is the information available? (Reagents and Tools Table, Materials and Methods, Figures, Data Availability Section)
Have **primary datasets** been deposited according to the journal's guidelines (see 'Data Deposition' section) and the respective accession numbers provided in the Data Availability Section?	Yes	Data Availability and Source Data
Were **human clinical and genomic datasets** deposited in a public access-controlled repository in accordance to ethical obligations to the patients and to the applicable consent agreement?	Not Applicable	
Are **computational models** that are central and integral to a study available without restrictions in a machine-readable form? Were the relevant accession numbers or links provided?	Not Applicable	
If publicly available data were reused, provide the respective **data citations in the reference list**.	Not Applicable	
